# A Dual-Mechanism Targeted Cross-Convergence Prodrug Activation Strategy Enabled by Biological Stimulus Amplification toward Precision Cancer Therapy

**DOI:** 10.34133/bmr.0328

**Published:** 2026-05-20

**Authors:** Zeyu Wang, Yajie Xing, Dong Wang, Yumeng Niu, Fang Chen, Kun Fan, Xin Meng, Mi Li, Dandan Xia, Lan Wu, Haiyan Xu, Qi Guan, Weige Zhang

**Affiliations:** ^1^ Key Laboratory of Structure-Based Drug Design and Discovery, Ministry of Education, School of Pharmaceutical Engineering, Shenyang Pharmaceutical University, Shenyang 110016, China.; ^2^ School of Pharmacy, Shenyang Pharmaceutical University, Shenyang 110016, China.; ^3^ Department of Geratology, The First Affiliated Hospital, China Medical University, Shenyang 110001, China.

## Abstract

Current prodrug strategies in clinical practice still face several challenges, such as insufficient targeting specificity, substantial systemic toxicity, and complex administration protocols, which urgently need to be resolved. Here, we present a dual-mechanism targeted cross-convergence activation strategy, which couples tumor-targeting prodrug delivery with biological stimulus amplification to achieve precise and highly efficient drug release. The system comprises 2 components: (a) **BTW2** nanoparticles, a reactive oxygen species (ROS)-responsive prodrug conjugated to biotin for tumor-selective accumulation via the enhanced permeability and retention effect and biotin-receptor-mediated uptake, and (b) IR-808, a heptamethine dye that targets tumors through organic-anion-transporting polypeptides and amplifies intracellular ROS. Upon cross-convergence at tumor sites, IR-808 elevates ROS levels, triggering thioketal linker cleavage in **BTW2** nanoparticles to release **W436**, a potent tubulin inhibitor. The released **W436** further amplifies ROS, establishing a self-reinforcing positive feedback loop. This strategy enables precision therapy for primary and metastatic cancers via spatiotemporally controlled, self-reinforcing drug release.

## Introduction

Cancer is one of the primary contributors to global mortality and remains a formidable challenge for medical researchers [1]. Although chemotherapy is one of the most commonly used and effective approaches for the treatment of cancer, its systemic toxicity may cause severe side effects that seriously affect patients’ quality of life (QOL) and also compromise therapeutic efficacy [2]. The construction of stimulus-responsive activable prodrugs has emerged as a promising strategy for targeted cancer therapy to overcome these drawbacks [3–5].

At present, there are 3 main categories of prodrug activation strategies. (a) Endogenous stimuli, as the most commonly approved activation strategy, rely on unique tumor microenvironment factors (e.g., pH, enzymes, and redox state) for prodrug activation. However, the subtle differences between diseased and normal tissues result in insufficient specificity, often leading to uncontrolled and inefficient drug release [6–8]. Furthermore, due to the heterogeneity of patients and cancer types, the broad-spectrum clinical application of endogenous-responsive prodrugs is still a major challenge [9,10]. (b) Exogenous stimuli (e.g., light, heat, and ultrasound) allow remote prodrug activation [11,12] but are generally restricted to localized tumors and cannot effectively treat distant metastases, which cause the majority of cancer-related deaths [13–16]. Additionally, extensive irradiation may damage nearby healthy tissues [17,18]. (c) Bio-orthogonal reaction-based strategies can overcome tumor heterogeneity and improve circulation stability and allow for on-demand activation within tumors [4,19–21]. Yet few reactions meet the requirements for both fast reaction kinetics and traceless payload release with high efficiency. Furthermore, as the prodrug is immediately activated upon encounter between the 2 components, they must be administered separately in a stepwise manner to prevent premature activation [22,23]. This staggered administration mode would not only bring great operational inconvenience for clinical application but might also reduce the therapeutic effect due to the difficulty of components reaching the target site at the optimal ratio and the premature metabolism of the early-arrival component [24]. Particularly, these problems would further be amplified in vivo as a result of the complicated internal environment [25]. In summary, while existing prodrug activation strategies have been extensively investigated in both the laboratory and clinic, they lack precise control over the activation location and sustained drug release at the target site. There remains a pressing need for innovative strategies that can amplify the difference between tumor and normal cells to enhance targeted therapy.

Here, we propose a dual-mechanism cross-convergence prodrug activation strategy that orchestrates 2 distinct tumor-targeting mechanisms, each being applied to one respective component, ensuring their exclusive encounter at the tumor site for spatiotemporally controlled drug release. Specifically, this approach involves 2 components, one of which is a small-molecule prodrug and the other is a targeting amplifier as the trigger that can sustainably enhance a specific biological stimulus, resulting in the activation of the prodrug accumulated at the tumor site. This “cross-convergence” design establishes an indirect, condition-dependent method for prodrug activation whereby the prodrug and the trigger are biologically inert when separate, with activation occurring only as the 2 components cross-converge at the tumor and a specific biological stimulus is amplified. Compared to endogenous stimulus-based strategies, which often rely on modest differentials such as a pH shift of 0.5 to 1.0 units or a 10-fold change in glutathione concentration [26,27], our strategy additionally amplifies a specific stimulus on top of such existing differentials, thereby creating a more pronounced differential and improving selectivity. Unlike spatially constrained exogenous stimuli such as light (with tissue penetration <1 cm) or ultrasound (confined to focal zones) [28,29], the targeted components can reach metastatic lesions via systemic circulation, offering a potential solution for treating metastatic tumors. Moreover, this “cross-convergence” mechanism is fundamentally distinct from bio-orthogonal approaches. Rather than relying on the direct covalent conjugation of bio-orthogonal functional groups (with reaction rates up to 10^6^ M^−1^s^−1^) that risks premature activation in circulation [30], our strategy is based on an indirect effect between 2 cross-convergence components within tumor cells, thereby effectively preventing premature activation.

**Fig. 1.**
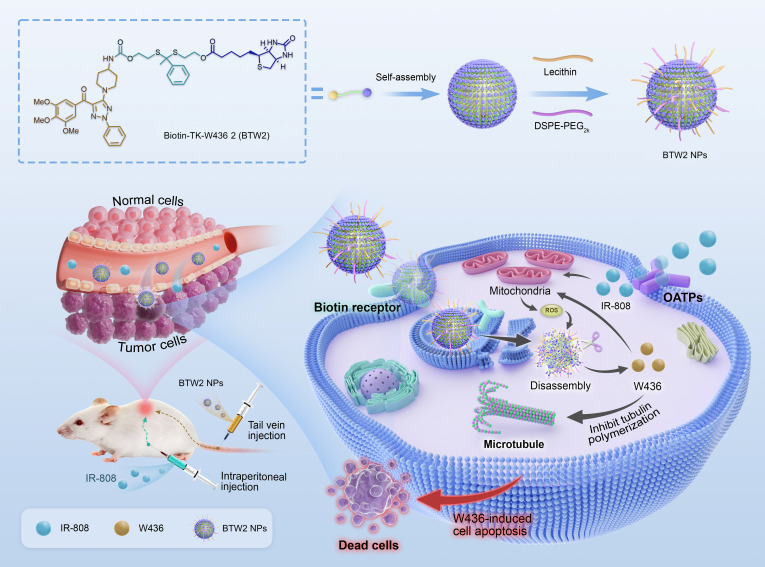
Mechanistic insights into IR-808-promoted activation of BTW2 nanoparticles (NPs) for enhanced antitumor efficacy. DSPE-PEG_2k_, 1,2-distearoyl-*sn*-glycero-3-phosphoethanolamine-*N*-[methoxy(polyethyleneglycol)-2000]; ROS, reactive oxygen species; OATPs, organic-anion-transporting polypeptides.

As proof of concept, the designed biotin–thioketal–colchicine binding site inhibitor (CBSI) conjugate (Biotin-TK-**W436** 2, **BTW2**), as a tumor-targeting small-molecule prodrug, was effectively targeted to the tumor site via self-assembled nanoparticles (NPs) (**BTW2** NPs), leveraging the high expression of biotin receptors on the cell membrane and the enhanced permeability and retention (EPR) effect. This design not only addresses the poor solubility of the prodrug but also incorporates a thioketal (TK) linker that is only cleaved by high levels of reactive oxygen species (ROS). Furthermore, we selected the tumor-targeting heptamethine cyanine dye IR-808 as the trigger, which accumulates in tumors and produces high intracellular ROS to cleave the TK linker in **BTW2** NPs upon their cross-convergence. As a result, this releases the active antitumor agent **W436**, thereby inducing tumor cell apoptosis. Notably, **W436** itself can further elevate ROS levels in tumor cells, establishing a positive feedback loop that promotes additional prodrug activation. The necessity for both components to converge at the tumor site provides a dual layer of targeting specificity (Fig. 1). In vivo antitumor studies demonstrated that the **BTW2** NPs + IR-808 combination group achieved a markedly higher tumor inhibition rate (85.4%) compared to **BTW2** NPs alone (53.2%) while exhibiting markedly reduced systemic toxicity relative to the **W436** group. Collectively, this work provides a new prodrug activation strategy that involves distinct tumor-targeting mechanisms and allows the convergence of prodrug and trigger inside tumor. This approach thereby facilitates on-target and on-demand prodrug liberation with enhanced anticancer efficacy and minimized adverse effects.

## Materials and Methods

All reagents and solvents were purchased from commercial sources and used without purification. Reactions were monitored by analytical thin-layer chromatography on silica gel plates (0.25-mm layer, Qingdao Haiyang Chemical Co. Ltd.) under ultraviolet light (wavelengths: 365 and 254 nm). ^1^H (400 MHz) and ^13^C nuclear magnetic resonance (NMR) (100 MHz) spectra were recorded on a Bruker AVANCE 400 using dimethyl sulfoxide-*d*_6_ (DMSO-*d*_6_) or CDCl_3_ as solvent at room temperature. Mass spectrometry (MS) was performed by Agilent Co. Ltd. on an Agilent 1100-sl mass spectrometer equipped with an electrospray ionization ESI source. High-resolution accurate mass determinations (high-resolution mass spectrometry [HRMS]) were performed on an Agilent 6530 Accurate-Mass Q-TOF LC/MS (Agilent Co. Ltd.) equipped with ESI. High-performance liquid chromatography (HPLC) analysis was performed using an HPLC-LC-20AT system (Shimadzu) equipped with an SPD-20A UV detector and a C18 analytical column (4.6 × 250 mm, 0.45 μm). The analysis was conducted with 20% methanol (MeOH) in water as the mobile phase at a flow rate of 1.0 ml min^−1^. The structures and synthetic numbering of the following compounds are detailed in Schemes S1 to S3.

### Synthesis of 3,4,5-trimethoxybenzoate (2)

Sulfuric acid (4 ml) was added dropwise to a stirred solution of 3,4,5-trimethoxybenzoic acid (**1**) (10.0 g, 46.6 mmol) in MeOH (100 ml). The reaction mixture was refluxed for 8 h. At the end of the reaction, the mixture was concentrated under reduced pressure. The crude product was dissolved with water (20 ml) and extracted with ethyl acetate (AcOEt) (20 ml × 4). The combined organic layers were washed with brine, dried over anhydrous Na_2_SO_4_, filtered, and concentrated to give **2** as a white solid. Yield: 94.1%; ^1^H NMR (400 MHz, CDCl_3_) *δ* 7.22 (s, 2H), 3.83 (s, 12H). ^13^C NMR (100 MHz, CDCl_3_) *δ* 165.7, 151.9 (2C), 141.2, 124.1, 105.8 (2C), 59.9, 55.2 (2C), 51.2. MS (ESI): *m*/*z* calcd. for C_11_H_14_O_5_ [M + H]^+^: 227.1; found: 227.1 (Fig. S1).

### Synthesis of 3-oxo-3-(3,4,5-trimethoxyphenyl) prop-anenitrile (3)

According to previously published methods, 3,4,5-trimethoxybenzoic acid (10.0 g, 44.2 mmol) and NaH (88.4 mmol, 3.04 g of 60% dispersion in mineral oil) were dissolved in acetonitrile (ACN; 50 ml). After refluxing for 6 h, the reaction was complete. The solvent was removed by means of a rotary evaporator. The residue was dissolved in 50 ml of water, and the aqueous solution was acidified to pH 2 with 2 mol/l HCl solution (100 ml). The precipitate was filtered to give **3** as a yellow solid. Yield: 87.3%; ^1^H NMR (400 MHz, CDCl_3_) *δ* 7.05 (s, 2H), 4.04 (s, 2H), 3.85 (s, 3H), 3.83 (s, 6H). ^13^C NMR (100 MHz, CDCl_3_) *δ* 185.3, 152.3 (2C), 142.9, 128.4, 113.2, 105.1 (2C), 60.0, 55.4 (2C), 28.3. MS (ESI): *m*/*z* calcd. for C_12_H_13_NO_4_ [M + H]^+^: 236.1; found: 236.1 (Fig. S2).

### Synthesis of 3-oxo-3-(3,4,5-trimethoxyphenyl)-2-(phenylhydrazono) propanenitrile (4)

Sodium nitrite (88 mg, 1.3 mmol) was added to a solution of aniline (166 mg, 1.2 mmol) in 6 M HCl (5 ml) at 0 °C, and the reaction mixture was stirred for 30 min to give a solution of the diazonium salt. The resulting solution of the diazonium salt was added dropwise to a solution of 3-oxo-3-(3,4,5-trimethoxyphenyl)propane nitrile (1.5 g, 6.4 mmol) in ethanol mixture (10 ml) with sodium acetate trihydrate (0.95 g, 11.6 mmol) at 0 °C. The mixture was then stirred at room temperature for 1 h, and the resulting solid was collected by filtration and washed with ice water and ethanol to give **4** as a yellow solid. Yield: 75.7%; ^1^H NMR (400 MHz, DMSO-*d*_6_) *δ* 12.33 (s, 1H), 7.48 (d, *J* = 7.80 Hz, 2H), 7.41 (t, *J* = 7.40 Hz, 3H), 7.30 (s, 2H), 7.17 (t, *J* = 7.20 Hz, 1H), 3.83 (s, 6H), 3.80 (s, 3H). ^13^C NMR (100 MHz, DMSO-*d*_6_) *δ* 190.9, 157.5 (2C), 147.3, 146.6, 136.4, 134.7 (2C), 130.2, 121.5 (2C), 118.4, 116.7, 113.1 (2C), 65.4, 61.2 (2C). MS (ESI): *m*/*z* calcd. for C_18_H_17_N_3_O_4_ [M + H]^+^: 340.1; found: 340.1 (Fig. S3).

### Synthesis of 5-(4-*N-*Boc-piperidine-yl)-2-phenyl-4-(3,4,5-trimethyloxybenzoyl)-1,2,3-triazol (5)

Compound **4** (5.0 g, 22.1 mmol), *tert*-butylpiperidin-4-ylcarbamate, and Cu(AcO)_2_ (0.8 g, 4.0 mmol) were suspended in ACN (50 ml). The reaction mixture was refluxed for 5 h in an open-air atmosphere. At the end of the reaction, the mixture was filtered. The solvent was removed under reduced pressure, and **5** was recrystallized from ethanol as a yellow solid. Yield: 74.9%; ^1^H NMR (400 MHz, CDCl_3_) *δ* 8.02 (d, *J* = 7.60 Hz 2H), 7.52 (s, 2H), 7.46 (t, *J* = 7.48 Hz, 2H), 7.34 (t, 7.36 Hz, 1H), 3.95 (s, 3H), 3.92 (s, 6H), 3.87 (t, *J* = 13.6 Hz, 2H), 3.68 (s 1H), 3.03 (t, *J* = 11.28 Hz, 2H), 2.06 (d, *J* = 10.04 Hz, 2H), 1.64 (d, *J* = 15.16 Hz, 2H), 1.46 (s, 9H). ^13^C NMR (100 MHz, CDCl_3_) *δ* 184.5, 157.1, 154.2, 151.7 (2C), 141.5, 138.3, 133.0, 131.8, 128.3 (2C), 126.7, 117.5 (2C), 107.1 (2C), 78.4, 60.0, 55.3 (3C), 47.5, 31.1, 27.4 (3C). MS (ESI): *m*/*z* calcd. for C_28_H_35_N_5_O_6_ [M + H]^+^: 538.3; found: 538.1 (Fig. S4).

### Synthesis of 5-(4-amino-piperidine-yl)-2-phenyl-4-(3,4,5-trimethyloxybenzoyl)-1,2,3-triazole (W436)

Trifluoroacetic acid (10 ml) was added to a solution of compound **5** (5.0 g, 11.4 mmol) in dichloromethane (DCM) (10 ml) at room temperature. At the end of the reaction, the pH was adjusted to 10 with 1 mol/l Na_2_CO_3_ solution. The solution was then extracted with DCM (20 ml × 4). The combined organic layers were washed with brine, dried over anhydrous Na_2_SO_4_, filtered, and concentrated to give **W436** as a yellow solid. Yield: 96.3%; ^1^H NMR (400 MHz, DMSO-*d*_6_) *δ* 7.97 (d, *J* = 6.84 Hz, 2H), 7.58 (s, 2H), 7.46 (s, 3H), 3.86 (s, 9H), 3.79 (s, 2H), 3.12 (s, 1H), 2.97 (t, *J* = 10.8 Hz, 2H), 1.95 (m, 2H), 1.63 (m, 2H), 1.22 (s, 2H). ^13^C NMR (100 MHz, DMSO-*d*_6_) *δ* 185.3, 157.6, 152.9 (2C), 142.5, 139.2, 134.0, 132.9, 130.3 (2C), 128.4, 118.7 (2C), 108.3 (2C), 60.7, 56.5 (2C), 47.9, 47.7 (2C), 31.0 (2C). HRMS (ESI): *m*/*z* calcd. for C_23_H_27_N_5_O_4_ [M + Na]^+^: 460.1961; found: 460.1994 (Fig. S5). Purity: 99.2% by HPLC (Fig. S6).

### General procedure for the preparation of compounds 7a to 7b

Trifluoroacetic acid was added dropwise to a mixture of thioglycolic acid and ketone. The reaction mixture was stirred at room temperature for 1 h, and the resulting solid was collected by filtration and washed with *n*-hexane and ice water.

#### 2,2′-(Propane-2,2-diylbis(sulfanediyl))diacetic acid (7a)

Compound **7a** was obtained from thioglycolic acid and acetone (**6a**) as a white solid. Yield: 98.6%; ^1^H NMR (400 MHz, DMSO-*d*_6_) *δ* 3.37 (s, 4H), 1.56 (s, 6H). ^13^C NMR (100 MHz, DMSO-*d*_6_) *δ* 171.8 (2C), 56.7, 33.3 (2C), 30.6 (2C). MS (ESI): *m*/*z* calcd. for C_7_H_12_O_4_S_2_ [M–H]^−^: 223.0; found: 222.8 (Fig. S7).

#### 2,2′-((1-Phenylethane-1,1-diyl)bis(sulfanediyl))diacetic acid (7b)

Compound **7b** was obtained from thioglycolic acid and acetophenone (**6b**) as a white solid. Yield: 94.6%; ^1^H NMR (400 MHz, DMSO-*d*_6_) *δ* 9.10 (s, 2H), 7.69 (d, *J* = 7.60 Hz, 2H), 7.40 (t, *J* = 7.32 Hz, 2H), 7.32 (t, *J* = 7.24 Hz, 1H), 3.33 (q, *J* = 32.12 Hz, 4H), 1.99 (s, 3H). ^13^C NMR (100 MHz, DMSO-*d*_6_) *δ* 171.3 (2C), 142.6, 128.8 (2C), 128.2, 61.4, 34.0 (2C), 29.7. MS (ESI): *m*/*z* calcd. for C_12_H_14_O_4_S_2_ [M–H]^−^: 285.0; found: 284.7 (Fig. S8).

#### 2,2′-(Cyclohexane-1,1-diylbis(sulfanediyl))diacetic acid (7c)

Compound **7c** was obtained from thioglycolic acid and cyclohexanone (**6c**) as a white solid. Yield: 92.3%; ^1^H NMR (400 MHz, DMSO-*d*_6_) *δ* 12.51 (s, 2H), 3.34 (s, 4H), 1.79 (t, *J* = 5.20 Hz, 4H), 1.57 (m, 4H), 1.41 (m, 2H). ^13^C NMR (100 MHz, DMSO-*d*_6_) *δ* 171.9 (2C), 62.8, 37.5 (2C), 31.7 (2C), 25.3, 22.5 (2C). MS (ESI): *m*/*z* calcd. for C_10_H_16_O_4_S_2_ [M–H]^−^: 263.0; found: 262.8 (Fig. S9).

### General procedure for the preparation of compounds 8a to 8c

Sodium borohydride (5.0 g, 0.132 mol) and the corresponding acid were suspended in dry tetrahydrofuran (THF). The reaction mixture was cooled to 0 °C, after which iodine (20.0 g, 0.057 mol) in dry THF (100 ml) was added slowly through a funnel over 1 h. The reaction mixture was then heated to reflux for 24 h. After cooling the mixture to room temperature, MeOH was added dropwise until the mixture became clear and the solvent was removed under reduced pressure. The residue was further dissolved in NaOH solution (25%, 200 ml) and extracted with AcOEt (5 × 100 ml). The combined organic layers were washed with brine, dried over anhydrous Na_2_SO_4_, filtered, and concentrated to give the crude product, which was purified by column chromatography on silica gel with light petroleum/AcOEt (v/v = 1:1).

#### 2,2′-(Propane-2,2-diylbis(sulfanediyl))bis(ethan-1-ol) (8a)

Compound **8a** was obtained from **7a** as a colorless oil. Yield: 52.4%; ^1^H NMR (400 MHz, CDCl_3_) *δ* 4.78 (s, 2H), 3.53 (t, *J* = 4.76 Hz, 4H), 2.66 (t, *J* = 4.76 Hz, 4H), 1.53 (s, 6H). ^13^C NMR (100 MHz, CDCl_3_) *δ* 60.3 (2C), 54.9, 32.6 (2C), 30.2 (2C). MS (ESI): *m*/*z* calcd. for C_7_H_16_O_2_S_2_ [M + Na]^+^: 219.1; found: 219.0 (Fig. S10).

#### 2,2′-((1-Phenylethane-1,1-diyl)bis(sulfanediyl))bis(ethan-1-ol) (8b)

Compound **8b** was obtained from **7b** as a colorless oil. Yield: 61.3%; ^1^H NMR (400 MHz, CDCl_3_) *δ* 7.49 (m, 1H), 7.35 (m, 2H), 7.27 (m, 2H), 3.67 (m, 4H), 2.77 (t, *J* = 6.24 Hz, 4H), 2.05 (s, 3H). ^13^C NMR (100 MHz, CDCl_3_) *δ* 142.0, 123.4 (2C), 122.5, 120.1 (2C), 66.0 (2C), 59.3, 29.7, 27.6 (2C). MS (ESI): *m*/*z* calcd. for C_12_H_18_O_2_S_2_ [M + Na]^+^: 281.2; found: 281.1 (Fig. S11).

#### 2,2′-(Cyclohexane-1,1-diylbis(sulfanediyl))bis(ethan-1-ol) (8c)

Compound **8c** was obtained from **7c** as a colorless oil. Yield: 54.6%; ^1^H NMR (400 MHz, CDCl_3_) *δ* 4.55 (s, 2H), 3.51 (t, *J* = 7.00 Hz, 4H), 2.63 (t, *J* = 7.20 Hz, 4H), 1.77 (t, *J* = 5.56 Hz, 4H), 1.56 (m, 4H), 1.40 (m, 4H). ^13^C NMR (100 MHz, CDCl_3_) *δ* 57.0, 56.6 (2C), 33.4 (2C), 26.8 (2C), 20.7, 17.6 (2C). MS (ESI): *m*/*z* calcd. for C_10_H_20_O_2_S_2_ [M + Na]^+^: 259.1; found: 259.1 (Fig. S12).

### General procedure for the preparation of compounds 9a to 9c

To a solution of compounds ****8**a** to****8**c** (1.0 g, 6.5 mmol) and triethylamine (1.0 ml, 6.5 mmol) in THF (10 ml), 4-nitrophenyl carbonochloridate (1.2 g, 5.8 mmol) was added at 0 °C. The reaction mixture was then stirred at room temperature for 8 h. The resulting white precipitate was removed by filtration, and the filtrate was concentrated under reduced pressure. The residue was charged with 50 ml of DCM and washed sequentially with saturated NaHCO_3_ solution (5 × 50 ml) and brine. The organic layers were dried over anhydrous Na_2_SO_4_, filtered, and concentrated to give the crude product, which was purified by column chromatography on silica gel with light petroleum/AcOEt (v/v = 4:1).

#### 2-((2-((2-Hydroxyethyl)thio)propan-2-yl)thio)ethyl (4-nitrophenyl) carbonate (9a)

Compound **9a** was obtained from **8a** and 4-nitrophenyl carbonochloridate as a colorless oil. Yield: 41.0%; ^1^H NMR (400 MHz, CDCl_3_) *δ* 8.19 (d, *J* = 9.20 Hz, 2H), 7.32 (d, *J* = 9.20 Hz, 2H), 4.37 (t, *J* = 7.20 Hz, 2H), 3.71 (t, *J* = 6.40 Hz, 2H), 2.93 (t, *J* = 7.20 Hz, 2H), 2.77 (t, *J* = 6.00 Hz, 2H), 1.56 (s, 6H). ^13^C NMR (100 MHz, CDCl_3_) *δ* 155.4, 152.3, 145.4, 125.3 (2C), 121.8 (2C), 68.0, 61.5, 56.4, 33.5, 31.0 (2C), 28.8. MS (ESI): *m*/*z* calcd. for C_14_H_19_NO_6_S_2_ [M + Na]^+^: 384.1; found: 384.0 (Fig. S13).

#### 2-((1-((2-Hydroxyethyl)thio)-1-phenylethyl)thio)ethyl (4-nitrophenyl) carbonate (9b)

Compound **9b** was obtained from **8b** and 4-nitrophenyl carbonochloridate as a colorless oil. Yield: 45.7%; ^1^H NMR (400 MHz, CDCl_3_) *δ* 8.18 (d, *J* = 9.20 Hz, 2H), 7.40 (d, *J* = 7.60 Hz, 2H), 7.30 (m, 2H), 7.25 (m, 2H), 7.15 (m, 1H), 4.30 (t, *J* = 6.40 Hz, 2H), 3.58 (t, *J* = 6.00 Hz, 2H), 2.82 (t, *J* = 6.80 Hz, 2H), 2.69 (t, *J* = 6.00 Hz, 2H), 1.81 (s, 3H). ^13^C NMR (100 MHz, CDCl_3_) *δ* 154.6, 151.5, 146.0, 144.7, 127.4 (2C), 126.4, 124.5 (2C), 124.1 (2C), 121.0 (2C), 69.9, 69.4, 63.3, 33.7, 31.6, 22.2. MS (ESI): *m*/*z* calcd. for C_19_H_21_NO_6_S_2_ [M + Na]^+^: 446.1; found: 446.0 (Fig. S14).

#### 2-((1-((2-Hydroxyethyl)thio)cyclohexyl)thio)ethyl (4-nitrophenyl) carbonate (9c)

Compound **9c** was obtained from **8c** and 4-nitrophenyl carbonochloridate as a colorless oil. Yield: 40.7%; ^1^H NMR (400 MHz, CDCl_3_) *δ* 8.21 (d, *J* = 9.2 Hz, 2H), 7.32 (d, *J* = 9.2 Hz, 2H), 4.37 (t, *J* = 6.8 Hz, 2H), 3.72 (t, *J* = 6.0 Hz, 2H), 2.93, (t, *J* = 6.8 Hz, 2H), 2.77 (t, *J* = 6.0 Hz, 2H), 1.80 (m, 4H), 1.58 (m, 4H), 1.40 (m, 2H). ^13^C NMR (100 MHz, CDCl_3_) *δ* 155.5, 152.3, 145.4, 125.3 (2C), 121.8 (2C), 68.2, 62.4, 61.6, 38.2 (2C), 32.1, 27.3, 25.4, 22.4 (2C). MS (ESI): *m*/*z* calcd. for C_17_H_23_NO_6_S_2_ [M + Na]^+^: 424.1; found: 424.0 (Fig. S15).

### General procedure for the preparation of compounds 10a to 10c

A solution of **W436** (1.5 g, 4.1 mmol) in THF was added dropwise to a solution of compounds **9a** to **9c** in THF. The reaction mixture was then stirred at room temperature for 6 h. The solvent was then removed under reduced pressure. The residue obtained was dissolved in 15 ml of DCM and washed with saturated NaHCO_3_ solution (3 × 25 ml) and brine (3 × 20 ml). The organic layers were dried over anhydrous Na_2_SO_4_, filtered, and concentrated to give the crude product, which was purified by column chromatography on silica gel with DCM/MeOH (v/v = 40:1).

#### 10a

Compound **10a** was obtained from **9a** as a yellow solid. Yield: 82.2%; ^1^H NMR (400 MHz, CDCl_3_) *δ* 8.02 (d, *J* = 8.20 Hz, 2H), 7.53 (s, 2H), 7.47 (t, *J* = 7.60 Hz, 2H), 7.35 (t, *J* = 7.44 Hz, 1H), 5.06 (d, *J* = 7.56 Hz, 1H), 4.24 (t, *J* = 6.24 Hz, 2H), 3.96 (s, 3H), 3.93 (s, 6H), 3.88 (m, 2H), 3.81 (t, *J* = 6.00 Hz, 2H), 3.72 (m, 1H), 3.04 (t, *J* = 11.28 Hz, 2H), 2.92 (t, *J* = 6.56 Hz, 2H), 2.88 (t, *J* = 5.96 Hz, 2H), 2.08 (dd, *J*_1_ = 12.68 Hz, *J*_2_ = 2.76 Hz, 2H), 1.68 (m, 2H), 1.63 (s, 6H). ^13^C NMR (100 MHz, CDCl_3_) *δ* 185.68, 158.13, 152.74 (2C), 139.29, 133.98, 132.77, 129.39 (2C), 127.83, 126.20 (2C), 118.54 (2C), 108.07 (2C), 63.51, 61.29, 61.03, 56.29 (2C), 63.51, 61.29, 61.04 (2C), 56.29 (3C), 48.41 (2C), 33.67 (2C), 31.88, 31.08 (2C), 29.69. HRMS calcd. for C_31_H_41_N_5_NaO_7_S_2_ [M + Na]^+^: 682.2345; found: 682.2357 (Fig. S16).

#### 10b

Compound **10b** was obtained from **9b** as a yellow solid. Yield: 85.9%; ^1^H NMR (400 MHz, CDCl_3_) *δ* 7.95 (d, *J* = 7.88 Hz, 2H), 7.63 (d, *J* = 7.64 Hz, 2H), 7.45 (s, 2H), 7.39 (t, *J* = 7.64 Hz, 2H), 7.26 (m, 3H), 7.17 (m, 1H), 4.96 (s, 1H), 4.06 (t, *J* = 5.80 Hz, 2H), 3.88 (s, 3H), 3.85 (s, 6H), 3.79 (m, 2H), 3.61 (m, 5H), 2.96 (t, *J* = 11.36 Hz, 2H), 2.74 (t, *J* = 6.36 Hz, 2H), 2.69 (t, *J* = 6.24 Hz, 2H), 2.00 (m, 2H), 1.95 (s, 3H). ^13^C NMR (100 MHz, CDCl_3_) *δ* 185.50, 158.11, 152.72 (2C), 143.31, 142.45, 139.28, 133.97, 132.77, 129.37, 128.38 (2C), 127.63, 126.89, 118.51 (2C), 108.04 (2C), 63.27, 61.33, 61.07, 60.99 (2C), 55.27 (3C), 34.29 (2C), 31.86, 30.63, 30.40. HRMS calcd. for C_36_H_43_N_5_NaO_7_S_2_ [M + Na]^+^: 744.2502; found: 744.2508 (Fig. S17).

#### 10c

Compound **10c** was obtained from **9c** as a yellow solid. Yield: 78.5%; ^1^H NMR (400 MHz, CDCl_3_) *δ* 8.00 (d, *J* = 5.56 Hz, 2H), 7.51 (s, 2H), 7.45 (t, *J* = 7.68 Hz, 2H), 7.33 (t, *J* = 7.40 Hz, 1H), 5.15 (s, 1H), 4.20 (t, *J* = 5.80 Hz, 1H), 3.94 (s, 3H), 3.90 (s, 6H), 3.88 (m, 2H), 3.76 (t, 6.16 Hz, 2H), 3.72 (s, 1H), 3.03 (t, *J* = 11.36 Hz, 2H), 2.82 (t, *J* = 6.16 Hz, 2H), 2.05 (m, 2H), 1.90 (m, 2H), 1.84 (m, 4H), 1.63 (m, 6H), 1.43 (m, 2H). ^13^C NMR (100 MHz, CDCl_3_) *δ* 184.5, 161.7, 151.7 (2C), 141.5, 138.3, 133.0, 131.8, 128.4 (2C), 126.7, 125.1, 117.5 (2C), 107.1 (2C), 61.1, 60.0, 55.3 (3C), 49.6, 48.1, 47.4, 37.1 (2C), 35.6, 32.8, 30.9, 23.9, 21.4 (2C). HRMS calcd. for C_34_H_45_N_5_NaO_7_S_2_ [M + Na]^+^: 722.2658; found: 722.2650 (Fig. S18).

### General procedure for the preparation of compounds BTW1 to BTW3

Biotin (366 mg, 1.5 mmol), *N*,*N*′-dicyclohexylcarbodiimide (371 mg, 1.8 mmol), 4-dimethylaminopyridine (12.2 mg, 0.1 mmol), and compounds **10a** to **10c** were dissolved in DMF. The reaction mixture was stirred at room temperature for 24 h. After the reaction was complete, the reaction mixture was filled with 50 ml of DCM and washed sequentially with water (5 × 50 ml) and brine. The organic layers were dried over anhydrous Na_2_SO_4_, filtered, and concentrated to give the crude product, which was purified by column chromatography on silica gel with DCM/MeOH (v/v = 20:1).

#### BTW1

Compound **BTW1** was obtained from **10a** as a yellow solid. Yield: 61.1%; ^1^H NMR (400 MHz, CDCl_3_) *δ* 7.98 (d, *J* = 8.16 Hz, 2H), 7.49 (s, 2H), 7.44 (t, *J* = 7.58 Hz, 2H), 7.31 (t, *J* = 7.30 Hz, 1H), 6.01 (s, 1H), 5.64 (s, 1H), 4.44 (t, *J* = 7.01 Hz, 1H), 4.26 (t, *J* = 6.43 Hz, 1H), 4.23 (t, *J* = 6.72 Hz, 2H), 4.20 (m, 2H), 3.92 (s, 3H), 3.90 (s, 6H), 3.86 (s, 2H), 3.10 (m, 1H), 3.01 (t, *J* = 11.69 Hz, 2H), 2.84 (m, 4H), 2.69 (d, *J* = 13.15 Hz, 1H), 2.31 (t, *J* = 7.60 Hz, 2H), 2.04 (d, *J* = 12.56 Hz, 2H), 1.67 (m, 2H), 1.64 (m, 4H), 1.58 (s, 6H), 1.42 (m, 2H). ^13^C NMR (100 MHz, CDCl_3_) *δ* 185.48, 173.49, 163.82, 158.10, 155.51, 152.71 (2C), 142.44, 139.29, 133.95, 132.79, 129.26 (2C), 127.73, 118.49 (2C), 108.02 (2C), 63.50, 63.41, 60.96, 60.91 (2C), 60.13, 56.42, 56.27 (3C), 55.51, 53.49, 48.43, 48.10, 40.54, 33.81, 31.86, 30.95 (2C), 29.62, 29.08, 28.37, 28.23, 24.75. HRMS calcd. for C_41_H_55_N_7_NaO_9_S_3_ [M + Na]^+^: 908.3121; found: 908.3118 (Fig. S19). Purity: 96.9% by HPLC (Fig. S20).

#### BTW2

Compound **BTW2** was obtained from **10b** as a yellow solid. Yield: 63.4%; ^1^H NMR (400 MHz, CDCl_3_) *δ* 8.01 (d, *J* = 7.84 Hz, 2H), 7.70 (d, *J* = 7.84 Hz, 2H), 7.51 (s, *J* = 8.1, 2H), 7.46 (t, *J* = 8.15 Hz, 2H), 7.34 (t, *J* = 8.15 Hz, 2H), 7.24 (s, 1H), 5.73 (s, 1H), 5.06 (t, *J* = 6.64 Hz, 1H), 4.45 (t, *J* = 6.64 Hz, 1H), 4.28 (d, *J* = 3.58 Hz, 1H), 4.14 (m, 4H), 3.95 (s, 3H), 3.92 (s, 6H), 3.87 (s, 2H), 3.12 (m, 1H), 2.33 (m, 2H), 2.87 (m, 1H), 2.78 (m, 4H), 2.70 (d, *J* = 12.54 Hz, 1H), 2.31 (t, *J* = 7.16 Hz, 2H), 2.03 to 2.06 (m, 2H), 2.01 (s, 3H), 1.71 (s, 2H), 1.67 (d, *J* = 6.14 Hz, 3H), 1.63 (s, 2H), 1.43 (d, *J* = 7.68 Hz, 2H). ^13^C NMR (100 MHz, CDCl_3_) *δ* 185.52, 173.39, 158.12, 155.42, 152.74 (2C), 143.08, 142.47, 139.32, 133.98, 132.81, 129.74, 129.38 (2C), 128.44 (2C), 127.75 (2C), 126.93 (2C), 118.52 (2C), 108.03 (2C), 63.28, 61.95, 61.57, 61.00 (2C), 60.10, 56.29 (3C), 55.41, 53.46, 50.80, 48.44, 48.09, 40.55, 33.79, 31.90, 29.92, 29.70, 28.33, 24.73. HRMS calcd. for C_46_H_57_N_7_NaO_9_S_3_ [M + Na]^+^: 970.3278; found: 970.3276 (Fig. S21). Purity: 98.8% by HPLC (Fig. S22).

#### BTW3

Compound **BTW3** was obtained from **10c** as a yellow solid. Yield: 67.9%; ^1^H NMR (400 MHz, CDCl_3_) *δ* 8.00 (d, *J* = 7.84 Hz, 2H), 7.50 (s, 2H), 7.45 (t, *J* = 7.84 Hz, 2H), 7.32 (t, *J* = 7.24 Hz, 1H), 5.84 (s, 1H), 5.49 (s, 1H), 4.45 (t, *J* = 5.43 Hz, 1H), 4.27 (t, *J* = 4.83 Hz, 1H), 4.21 (t, *J* = 7.24 Hz, 4H), 3.93 (s, 3H), 3.90 (s, 6H), 3.86 (s, 2H), 3.12 (m, 1H), 3.02 (t, *J* = 11.46 Hz, 2H), 2.84 (t, *J* = 4.84 Hz, 4H), 2.70 (m, 1H), 2.32 (t, *J* = 7.24 Hz, 2H), 2.04 (d, *J* = 10.86 Hz, 2H), 1.82 (t, *J* = 5.12 Hz, 4H), 3.68 (m, 2H), 1.66 (m, 4H), 1.63 (m, 4H), 1.57 (m, 6H), 1.42 (m, 4H). ^13^C NMR (100 MHz, CDCl_3_) *δ* 183.83, 171.83, 162.03, 156.43, 153.87, 151.04 (2C), 140.77, 137.61, 132.28, 131.11, 127.68 (2C), 126.05, 116.82 (2C), 106.34 (2C), 61.92, 60.74, 60.28, 59.30, 58.54, 54.59 (3C), 53.79, 51.79, 49.89, 48.79, 46.76, 46. 40, 38.85, 36.36, 32.14, 30.19, 27.99, 26.68, 26.54, 26.35, 25.82, 23,80, 23.07, 20.70. HRMS calcd. for C_44_H_59_N_7_NaO_9_S_3_ [M + Na]^+^: 948.3434; found: 948.3438 (Fig. S23). Purity: 98.4% by HPLC (Fig. S24).

### Synthesis of compounds BW

Compound **W436** (200 mg, 0.56 mmol), biotin (151 mg, 0.62 mmol), *O*-(7-azabenzotriazol-1-yl)-*N*,*N*,*N*′,*N*′-tetramethyluronium hexafluorophosphate (HATU) (213 mg, 0.56 mmol), and triethylamine (113 mg, 1.12 mmol) were dissolved in DMF. The reaction mixture was stirred at room temperature for 12 h. When the reaction was complete, the reaction mixture was filled with 50 ml of DCM and washed sequentially with water (5 × 50 ml) and brine. The organic layers were dried over anhydrous Na_2_SO_4_, filtered, and concentrated to give the crude product, which was purified by column chromatography on silica gel with DCM/MeOH (v/v = 20:1). Yellow solid; yield: 81.0%; ^1^H NMR (400 MHz, CDCl_3_) *δ* 8.01 (d, *J* = 7.60 Hz, 2H), 7.51 (s, 2H), 7.46 (t, *J* = 7.56 Hz, 2H), 7.33 (t, *J* = 7.40 Hz, 1H), 4.51 (t, *J* = 6.96 Hz, 1H), 4.32 (t, *J* = 4.52 Hz. 1H), 3.95 (s, 3H), 3.92 (s, 6H), 3.91 (m, 2H), 3.13 (m, 1H), 3.01 (t, *J* = 11.68 Hz, 2H), 2.89 (m, 1H), 2.73 (d, *J* = 12.76 Hz, 1H), 2.22 (t, *J* = 7.40 Hz, 2H), 2.02 (m, 2H), 1.68 (m, 2H), 1.45 (m, 2H). ^13^C NMR (100 MHz, CDCl_3_) *δ* 184.6, 171.7, 157.1, 151.8 (2C), 141.6, 138.3, 133.0, 131.8, 128.4 (3C), 126.7, 117.5 (2C), 107.1 (2C), 60.9, 60.0, 59.3, 54.6 (2C), 49.7, 47.6, 45.3, 39.5, 35.1, 30.6 (2C), 27.2, 27.1, 24.7. HRMS calcd. for C_33_H_41_N_7_NaO_6_S [M + Na]^+^: 686.2737; found: 686.2741 (Fig. S25). Purity: 97.5% by HPLC (Fig. S26).

### Cell culture

#### Cell lines

The SGC-7901 (human gastric adenocarcinoma), A549 (human lung carcinoma), and MCF-7 (mastadenoma) cell lines were cultured in Dulbecco’s modified Eagle medium (DMEM) and RPMI 1640 medium containing 10% (v/v) heat-inactivated fetal bovine serum (FBS), 100 U/ml streptomycin, and 100 U/ml penicillin at 37 °C in a humidified atmosphere of 95% air and 5% CO_2_. All cell lines tested were purchased from the American Type Culture Collection (Manassas, VA).

### Antiproliferative activity assay

The antiproliferative activities of target compounds **W436**, **BTW1** to **BTW3**, **BW**, IR-808, and colchicine were determined by a standard 3-(4,5-dimethylthiazol-2-yl)-2,5-diphenyltetrazolium bromide (MTT) assay. Briefly, cells were seeded into 96-well plates at a density of 4 × 10^3^/well and incubated at 37 °C with 5% CO_2_ for 24 h. Triplicate wells were then treated with test compounds at different concentrations. After 72 h, the medium was removed and replaced with 20 μl of MTT (5 mg/ml in phosphate-buffered saline [PBS]). After 4 h of incubation, the medium containing MTT was removed and 150 μl of DMSO was added to each well. The optical density was read using a microplate reader at 492 nm. The 50% inhibitory concentration (IC_50_) was defined as the concentration that reduced the absorbance of the untreated wells by 50% of that of the vehicle in the MTT assay.

### Tubulin polymerization assay

The effect of compounds **W436** and colchicine on the polymerization of tubulin was determined by using a tubulin polymerization assay kit (Cytoskeleton, Cat. No. BK011P) according to the manufacturer’s protocol. Briefly, tubulin was resuspended in ice-cold G-PEM (glycerol-PIPES-EGTA-MgCl_2_) buffer (80 mM piperazine-*N*,*N*′-bis(2-ethanesulfonic acid) [PIPES], 2 mM MgCl_2_, 0.5 mM ethylene glycol tetraacetic acid [EGTA], 1 mM guanosine-5′-triphosphate, and 15% [v/v] glycerol) and added to the wells of a 96-well plate containing the indicated concentration of **W436**, colchicine, or vehicle. Samples were mixed well, and tubulin assembly was monitored (the emission wavelength was 450 nm; the excitation wavelength was 360 nm) at 1-min intervals for 90 min at 37 °C using an Infinite F500 microplate reader (Tecan, Switzerland).

### Immunofluorescence assay

Immunofluorescence studies were performed to detect microtubule-associated tubulin protein after exposure to colchicine and **W436**. MCF-7 cells were seeded into 12-well plates and grown for 24 h. Cells were then treated with vehicle control (0.1% DMSO), colchicine, and **W436** for 24 h. Control and treated cells were fixed with 4% formaldehyde in PBS for 30 min, washed twice with PBS, and permeabilized with 0.2% (v/v) Triton X-100 in PBS for 30 min. The cells were then blocked with 5% bovine serum albumin (BSA) in PBS for 30 min. The primary α-tubulin antibody (Santa Cruz, CA) was diluted (1:100) with 2% BSA in PBS and incubated at 4 °C overnight. The cells were then washed with PBS to remove unbound primary antibody and incubated with fluorescein isothiocyanate-conjugated anti-mouse secondary antibody diluted (1:100) with 2% BSA in PBS for 3 h at 37 °C. The cells were washed with PBS to remove unbound secondary antibody, the nucleus was stained with 4′,6-diamidino-2-phenylindole dihydrochloride, and immunofluorescence was detected by confocal microscopy.

### Cell cycle analysis

MCF-7 cells (1 × 10^5^ cells) were incubated with a concentration of twice the IC_50_ of colchicine, **W436**, or 0.05% DMSO for the indicated times. The cells were collected by centrifugation, washed with PBS, and fixed in ice-cold 70% ethanol. The fixed cells were collected by centrifugation and resuspended in 535 ml of buffer containing 10 ml of RNase and 25 ml of propidium iodide. After incubation at 37 °C for 30 min in the dark, samples were analyzed by FACScan flow cytometry (Becton Dickinson, Franklin Lakes, NJ, USA).

### Stability of prodrugs

Prodrugs **BTW1** to **BTW3** were dissolved in ACN to prepare 10 mM stock solutions. The solution (0.1 ml) was added separately to 5 ml of cell culture medium. The mixture was gently stirred at 37 °C for 72 h while the mixture was analyzed by HPLC.

### In vitro ROS generation

MCF-7 cells were seeded into 6-well plates (5 × 10^5^ cells/well) and cultured in DMEM with 10% FBS (2 ml/well) at 37 °C/5% CO_2_ for 24 h. After replacing the medium with fresh DMEM/10% FBS, cells were treated with IR-808 or **W436** at indicated concentrations for 24 h. The medium was then replaced with DMEM containing 10 μM 2′,7′-dichlorodihydrofluorescein diacetate for 30 min. Cells were washed 3 times with ice-cold PBS and analyzed by (a) flow cytometry and (b) confocal laser scanning microscopy to detect dichlorofluorescein fluorescence (Ex: 488 nm; Em: 500 to 540 nm), reflecting intracellular ROS levels.

### Western blotting assay

After treating MCF-7 cells with different concentrations of IR-808, total protein was extracted using a commercial kit. Samples were mixed with 4× loading buffer and denatured at 100 °C for 10 min. Proteins were separated by sodium dodecyl sulfate–polyacrylamide gel electrophoresis and transferred onto nitrocellulose membranes. Membranes were blocked with 5% skim milk and then incubated overnight at 4 °C with primary antibodies. After five 5-min Tris-buffered saline with Tween 20 (TBST) washes, membranes were incubated with secondary antibodies (room temperature, 1.5 h). Following additional TBST washes, protein bands were detected by enhanced chemiluminescence using a chemiluminescence imager.

### MMP assay

The mitochondrial membrane potential (MMP) was evaluated by JC-1 staining. Cells were divided into 4 groups: 1, control; 2, *N*-acetyl-l-cysteine (NAC); 3, **W436**; and 4, NAC + **W436**. After 1 h pretreatment with 5 mM NAC (groups 2 and 4), all groups except control were exposed to **W436** for 24 h. Cells were then stained with JC-1 (37 °C, 30 min, dark), and fluorescence images were acquired by a fluorescence microscope.

### Molecular docking

Molecular modeling studies were performed with the published x-ray structures of tubulin (PDB: 5LYJ) using Maestro (version 11.5.011). The protein structures were prepared using the Protein Preparation module with default settings. The ligands were prepared for docking using the Ligprep module at pH 7 ± 0.5. The receptor lattices were generated using the recommended parameters. Docking was performed without constraints. All figures were generated using PyMOL (version 2.3.1).

### In vitro drug release

To evaluate the **W436** release profiles, the prodrug **BTW2** (1.0 mg) or **BW** (1.0 mg) was incubated with 50 mM H_2_O_2_ in 5 ml of PBS (pH 7.4) (20% ACN [v/v]) containing CuCl_2_ (1.0 μM) at 37 °C. Subsequently, 0.5 ml of this solution was collected at specific time points. The released **6** was simultaneously determined by HPLC.

### Liver microsomal stability assay

Thirty microliters of 1.5 μM spiking solution from **BTW2** containing 0.75 mg/ml microsome solution was added to the assay plates designated for different time points (0, 5, 15, 30, and 45 min). For 0 min, 150 μl of ACN was added to the wells before adding 15 μl of NADPH stock solution (6 mM). For the other time points, 15 μl of nicotinamide adenine dinucleotide phosphate (NADPH) stock solution (6 mM) was added to the wells to start the reaction and timing. At 5, 15, 30, and 45 min, 150 μl of ACN was added to the wells of the corresponding plates to stop the reaction. After quenching, the plates were shaken for 10 min (600 rpm) and centrifuged at 6,000 rpm for 15 min; 80 μl of the supernatant from each well was transferred to a 96-well sample plate containing 140 μl of pure water for liquid chromatography (LC)–MS analysis.

### Targeting mechanism assay

MCF-7 cells were seeded into a 96-well plate at a concentration of 4 × 10^3^ cells per well and cultured at 37 °C with 5% CO_2_ for 24 h. Cells were then treated with **BTW2** (1 μM) and incubated for a further 72 h. For NAC pretreatment, cells were treated with 5 mM NAC for 1 h at 37 °C. For biotin treatment, biotin (10 μM) was added simultaneously. MTT (5 mg/ml in PBS, 20 μl) was added to each well, and the plate was kept at 37 °C for a further 4 h. Optical density was read using a microplate reader at 492 nm. Cell viability was expressed as a percentage of the relative absorbance compared to the control group.

### In vitro IR-808 triggered drug release

Briefly, MCF-7 cells were seeded into 96-well plates at a density of 4 × 10^3^/well. After 24 h, one group of cells was treated with **BTW2** at different concentrations. The other group was treated with **BTW2** in combination with IR-808 (10 μM). After 72 h, 20 μl of MTT (5 mg/ml in PBS) was added and incubated for 4 h. The optical density was detected using a microplate reader at 492 nm. Cell viability was expressed as a percentage of relative absorbance compared to the control group.

MCF-7 cells were seeded into 6-well plates at a density of 1 × 10^6^/well. Cells were incubated with IR-808 (10 μM), **W436** (50 nM + 10 μM), **W436** + IR-808 (50 nM + 10 μM), **BTW2** (1 μM), or **BTW2** + IR-808 (1 μM + 10 μM) for 36 h. Cells were collected by centrifugation, washed with PBS, and fixed in ice-cold 70% ethanol. The fixed cells were collected by centrifugation and resuspended in 535 ml of buffer containing 10 ml of RNase and 25 ml of propidium iodide. After incubation at 37 °C for 30 min in the dark, samples were analyzed by FACScan flow cytometry (Becton Dickinson, Franklin Lakes, NJ, USA).

MCF-7 cells were seeded into 12-well plates at a density of 1 × 10^5^/well and cultured for 24 h. The cells were then incubated with **BTW2** (5 μM) or **BTW2** + IR-808 (0.5 μM + 10 μM) for 12 or 36 h (*n* = 3). After incubation, the cells and drug-containing medium were collected together using cell scrapers, and the cells were also ruptured by freezing and thawing. The **W436** released was measured simultaneously by LC–MS after protein precipitation.

### Preparation of BTW2 NPs

Prodrug **BTW2** (6 mg), 1,2-distearoyl-*sn*-glycero-3-phosphoethanolamine-*N*-[methoxy(polyethyleneglycol)-2000] (DSPE-PEG_2k_) (1.2 mg), and lecithin (0.6 mg) were dissolved in ethanol (1 ml), and the solution was added dropwise into deionized water (4 ml) under stirring (1,000 rpm). Then, the ethanol was removed by a rotary evaporator under vacuum, and the prepared prodrug NPs were stored at 4 °C. The size and zeta potential of all prodrug NPs were measured using Zetasizer (Nano-ZS ZEN3700).

### In vivo biodistribution of BTW2 NPs

Animal experiments were conducted according to the *Guide for the Care and Use of Laboratory Animals* issued by the Institutional Animal Ethical Care Committee of Shenyang Pharmaceutical University (Application No. SYXK 2021-0009). 4T1 cells (5 × 10^6^) were injected subcutaneously into the right back of female BALB/c mice. When the tumor volume reached approximately 300 mm^3^, free 1,1-dioctadecyl-3,3,3,3-tetramethylindotricarbocyanine iodide (DiR) or DiR-labeled NPs (1 mg kg^−1^, DiR equivalent) (*n* = 3) were injected intravenously into the 4T1 tumor-bearing BALB/c mice. IR-808 (1 mg kg^−1^) (*n* = 3) was injected intraperitoneally into the mice. At 2, 6, 12, 24, and 48 h after injection, mice were anesthetized and observed using the IVIS Lumina III Small Animal Imaging System (PerkinElmer, USA). Mice were sacrificed 48 h after injection, and organs including the heart, liver, spleen, lung, and kidney and tumor were harvested to determine fluorescence intensity.

### In vivo antitumor efficacy

4T1 cells (5 × 10^6^) were injected subcutaneously into the right back of female BALB/c mice. 4T1 tumor-bearing mice with a tumor size of approximately 100 mm^3^ were used to evaluate the antitumor efficacy of different groups. Briefly, mice were randomly divided into 6 groups (*n* = 5), and saline, IR-808, **BW**, **BTW2** NPs, **W436**, and IR-808 + **BTW2** NPs (10 mg kg^−1^, IR-808 equivalent; 5 mg kg^−1^, **W436** equivalent) were injected intravenously into the tail vein 7 times every 2 d. Body weight and tumor volumes were recorded every 2 d (calculated volume = 0.5 × length × width^2^). On day 14, the mice were sacrificed and the major organs (heart, liver, spleen, lung, and kidney) and tumors were dissected. The tumors in each group were individually weighed, and histopathological analysis of major organs was performed using hematoxylin and eosin (H&E) staining. The tumor samples were stained with terminal deoxynucleotidyl transferase dUTP nick end labeling (TUNEL) and Ki67.

### Antitumor metastasis efficacy

The antitumor metastasis efficacy in vivo was investigated using 4T1-Luc mouse models of lung metastasis derived from breast cancer. To establish the model, 4T1-Luc cells were injected into the tail vein of the mice at a density of 5 × 10^5^ cells per mouse. On day 3 after tumor metastasis, the mice were randomly divided into 6 groups. Mice with tumor metastasis were treated with saline, IR-808, **BW**, **BTW2** NPs, **W436**, and IR-808 + **BTW2** NPs (10 mg kg^−1^, IR-808 equivalent; 5 mg kg^−1^, **W436** equivalent) by tail vein injection with an administration interval of 2 d, and lung metastatic tumor growth was monitored at each time point using bioluminescence imaging. During the pharmacodynamic test, the survival status of the surviving mice in each group was recorded daily and the body weights were recorded every 2 d. On day 14, the mice were sacrificed and their lung tissues were collected and placed in Bouin’s fixative to count metastatic foci on the surface. The lung metastasis inhibition rate was calculated as follows: Lung metastasis inhibition rate (%) = (Number_Model_ − Number_Treated_)/Number_Model_ × 100%, where Number_Model_ and Number_Treated_ are the number of metastatic foci in the model control group and the treatment groups, respectively.

### Statistical analysis

Data are presented as means ± SD. Comparison between groups was performed using one-way analysis of variance and the Student *t* test, and *P* < 0.05 was considered statistically significant.

## Results and Discussion

### Identification of a pretargeted trigger for regulating the endogenous stimulus intensity in vivo

Insufficient responsiveness can hinder effective or complete prodrug activation and drug release due to the low biological stimulus concentrations typically found at the tumor site, ultimately limiting the therapeutic efficacy of the stimulus-responsive prodrugs [7]. In order to achieve selectivity and efficient reactivity of drug release, we initiated our studies by constructing a tumor microenvironment oxidative stress amplifier as the trigger equipped with an excellent tumor-targeting property and an effective prodrug activation capacity. Such an amplifier would be capable of selectively amplifying the prodrug activation process once it has accumulated in tumor sites, achieving the generation of numerous bioactive drugs in situ under the tumor microenvironment for highly selective antitumor therapy.

**Fig. 2.**
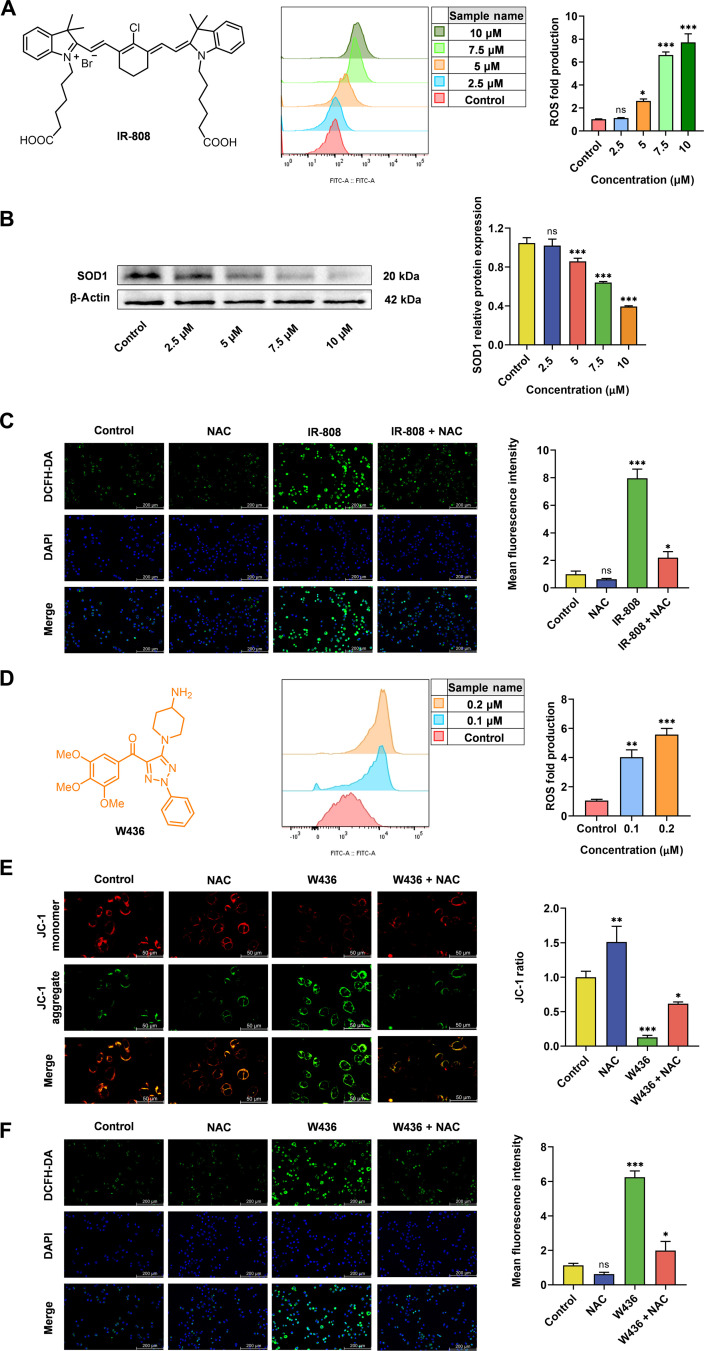
Application of the cross-convergence prodrug activation strategy in the activation of ROS-responsive prodrugs. (A) Twenty-four hours after treating with various doses of IR-808, the ROS production in MCF-7 cells was measured by flow cytometry (*n* = 3). (B) MCF-7 cells were treated with various doses of IR-808 for 24 h, and proteins were harvested for detection of superoxide dismutase 1 (SOD1) by western blotting analysis. Quantitative results are shown on the right. (C) MCF-7 cells treated with 10.0 μM IR-808 for 24 h were imaged by fluorescence microscopy. Quantification of mean fluorescence intensity is shown on the right (*n* = 3). (D) Twenty-four hours after treating with various doses of W436, the ROS production in MCF-7 cells was measured by flow cytometry (*n* = 3). (E) MCF-7 cells pretreated with or without 5 mM *N*-acetyl-l-cysteine (NAC) for 1 h prior to 24 h W436 exposure were stained with JC-1. Mitochondrial membrane potential (MMP) was assessed by fluorescence microscopy. Quantification of JC-1 red/green fluorescence ratio is shown on the right (*n* = 3). (F) MCF-7 cells treated with 0.2 μM W436 for 24 h were imaged by fluorescence microscopy. Quantification of mean fluorescence intensity are shown on the right (*n* = 3). Statistical significance: *P < 0.05, **P < 0.01, and ***P < 0.001 vs. control; ns, not significant (*P* ≥ 0.05). FITC, fluorescein isothiocyanate; DAPI, 4′,6-diamidino-2-phenylindole dihydrochloride; DCFH-DA, 2′,7′-dichlorodihydrofluorescein diacetate.

IR-808, a heptamethine indocyanine dye, preferentially accumulated in cancer cells as mediated by organic-anion-transporting polypeptides overexpressed in a variety of cancer cells [31], providing the potential for highly specific cancer diagnosis and imaging-guided treatment [32–34]. Prior studies established that IR-808 analogs elevate ROS via superoxide dismutase 1 (SOD1) suppression [35,36]. Here, we demonstrate the IR-808 itself induces sustained ROS production in cancer cells through analogous SOD1 inhibition independent of external stimuli (Fig. 2B). Flow cytometry and fluorescence microscopy analyses demonstrated that treating MCF-7 cells with 10 μM IR-808 for 24 h significantly increased ROS production (7.7-fold vs. control), which was effectively reversed by the ROS scavenger NAC (Fig. 2A and C). IR-808 demonstrated favorable biocompatibility profiles, with cellular viability assays revealing no notable adverse effects on the proliferation of multiple human cell lines following 72-h exposure at 50.0 μM (Fig. S27). Additionally, our studies in vivo showed that IR-808 could accumulate effectively in tumor tissue for a long time without damaging major organs. These results indicate the IR-808 is an ideal tumor-targeting trigger that continuously replenishes the intratumoral ROS pool for oxidative stress amplification once accumulated into tumor sites. In addition, the fluorescence property of IR-808 would realize the real-time imaging of tumors and metastases, assess treatment effectiveness, and guide the rational use of prodrugs. Our findings not only provide a paradigm for the development of tumor-targeting triggers for regulating the endogenous stimulus intensity in vivo but also present a new ROS enhancement strategy for prodrug release efficiency and selectivity and enhancing therapeutic efficacy

### Design and synthesis of the biotin–thioketal–CBSI conjugates (Biotin-TK-CBSI)

Having confirmed that the tumor-targeting trigger potently enhances intracellular endogenous stimuli, our study focused on the rational design of targeted prodrugs. Among various targeted approaches, small-molecule drug conjugates (a class of prodrugs linking tumor-targeting ligands to cytotoxic payloads via cleavable linkers) have gained considerable attention over the past several decades [37–39]. In the conjugated form, monomethyl auristatin E, as a potent tubulin inhibitor, is a validated cytotoxic payload in clinical settings, since the delivery platform overcomes the lack of tumor specificity of chemotherapeutics administered without the targeting ligand [40,41]. However, other tubulin inhibitors such as CBSIs are primarily underexplored. We previously demonstrated that **W436** exhibited robust antitumor activity by destabilizing tubulin polymerization through targeting the colchicine-binding site (Figs. S28 to S30). Additionally, **W436** enhanced the ROS levels in Hep3B and HepG2 cells and effectively inhibited their migration and invasion [42]. In this work, we demonstrate that **W436** elevates intracellular ROS levels by 5.8-fold at 0.2 μM in MCF-7 cells, mechanistically linked to MMP dissipation (Fig. 2D to F). Molecular docking studies suggested that the piperidin-4-amine moiety of **W436** occupies the hydrophilic cavity of the colchicine-binding site, forming 2 key hydrogen bonds with α-Gly246 and α-Ala247 (Fig. S31). Therefore, the amine group of **W436** is a key functional group that can be readily modified to effectively and temporarily mask its biological activity, thereby serving as a valuable linkage site for prodrug design. Thus, **W436** serves as a potent payload in this prodrug design, elevating intracellular ROS levels upon release and thereby establishing a positive feedback loop that enhances prodrug activation in cancer cells.

The minimization of off-target side effects and the targeted delivery of anticancer agents can be accomplished by anchoring a tumor-targeting moiety in a prodrug strategy. To this end, we selected biotin as a tumor-targeting ligand based on its high binding affinity to biotin receptors (sodium-dependent multivitamin transporter [SMVT]) that are overexpressed in malignancies. In breast cancer, SMVT expression is elevated 5- to 20-fold compared to that in normal mammary epithelial cells, driven by increased metabolic demand for biotin-dependent carboxylases (e.g., acetyl-CoA carboxylase) during rapid proliferation. Preclinical studies demonstrate that biotin-conjugated prodrugs achieve 8- to 12-fold higher tumor uptake than nontargeted counterparts [43].

**Fig. 3.**
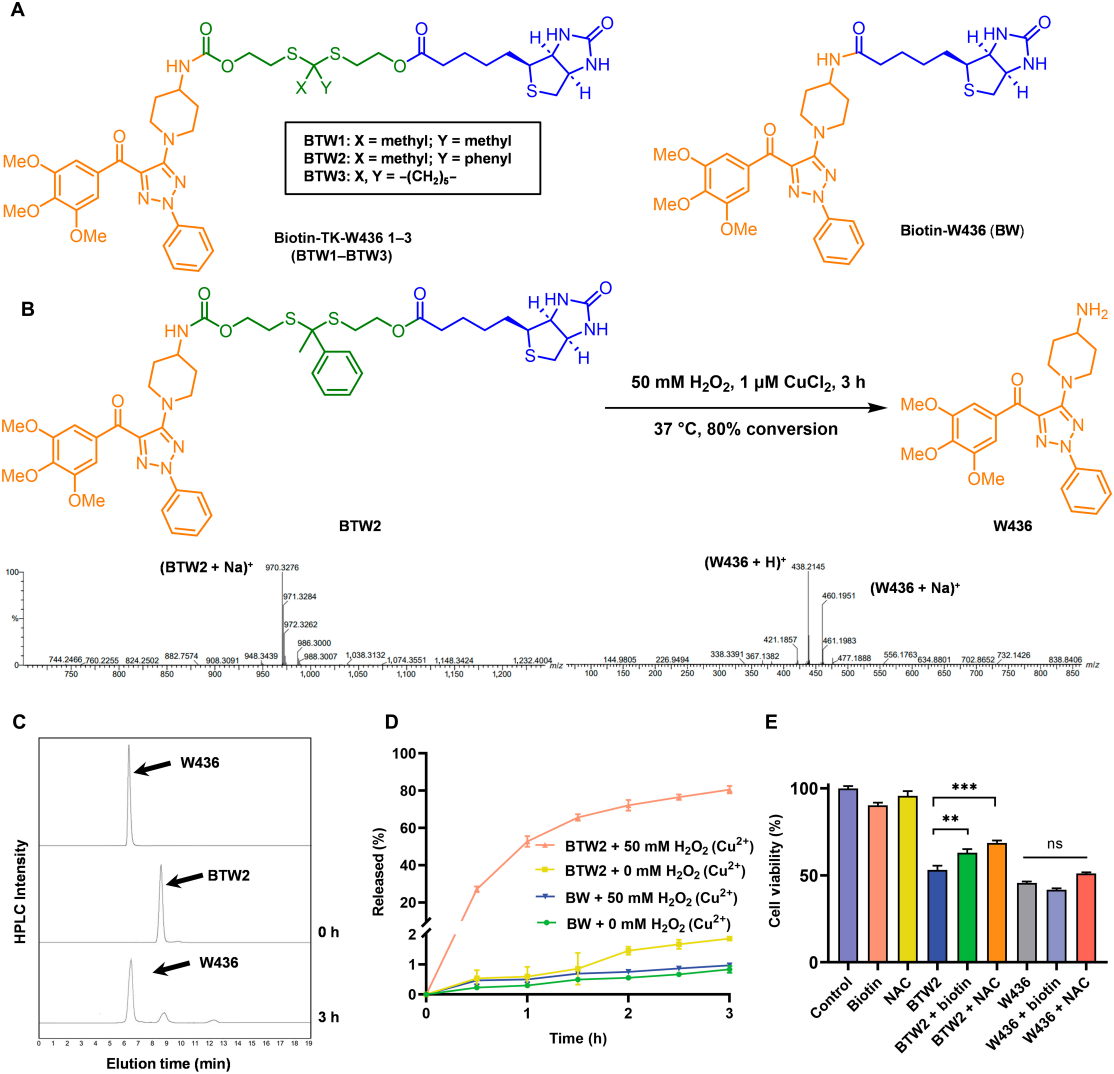
Characterization of the ROS-responsive prodrug BTW2. (A) The structures of BTW1 to BTW3 and BW. (B) Electrospray ionization (ESI)–mass spectrometry (MS) of BTW2 when incubated with 50.0 mM H_2_O_2_ and 1.0 μM CuCl_2_ for 1 h and the drug release mechanism. (C) High-performance liquid chromatography (HPLC) determination of BTW2 incubated with H_2_O_2_ (50.0 mM) in the presence of CuCl_2_ (1.0 μM). (D) Cumulative release of W436 from BTW2 and BW in the presence of CuCl_2_ (1.0 μM) with or without H_2_O_2_ (*n* = 3). (E) In vitro cytotoxicity of biotin, NAC, BTW2, BTW2 + biotin, BTW2 + NAC, W436, W436 + biotin, and W436 + NAC in MCF-7 cells (*n* = 3). The 3-(4,5-dimethylthiazol-2-yl)-2,5-diphenyltetrazolium bromide (MTT) assay showed cell viability after treatment with various formulations for 72 h. Statistical significance: **P < 0.01 and ***(*P* < 0.001); ns, not significant (*P* ≥ 0.05).

We subsequently focused our attention on the self-immolative linkers that connect biotin (targeting ligand) and **W436** (payload). Acetone-based dithioketal cleavable linkers have been extensively utilized as ROS-responsive components in polymeric prodrugs [44,45]. However, they have rarely been applied in the design of small-molecule ROS-responsive prodrugs, as excessive sensitivity can lead to premature activation and uncontrolled release of active drugs in vivo, ultimately resulting in unavoidable systemic toxicity. We surmised that the ketone unit of TK plays a crucial role in balancing the stability and responsiveness of prodrugs, and to our knowledge, no previous reports have addressed this aspect. To investigate the effects of different ketone units in TKs, in the design of biotin-coupled conjugated prodrugs Biotin-TK-**W436** 1 to 3 (**BTW1** to **BTW3**), we employ a hybridization strategy that integrates key features of our previously disclosed **W436**-based CBSI and biotin (Fig. 3A). In particular, the different TK-containing spacers with ROS sensitivity were then introduced to the amino group of the **W436** scaffold to sterically restrict access to the colchicine-binding pocket with a suitable linker. Moreover, we provide a simple method to synthesize these conjugates. Notably, **W436** was prepared according to our previous work (Scheme S1). The synthetic route of the conjugates **BTW1** to **BTW3** is shown in Scheme S2. Additionally, we also synthesized Biotin-**W436** (**BW**), a direct covalent conjugate of W436 and biotin, which could not be degraded by ROS, to serve as a negative control (Scheme S3). Finally, the selectivity profiles of these prodrugs were evaluated by comparing their cytotoxic effects between cancer cells and normal endothelial cells (human umbilical vein endothelial cells [HUVECs]) using the MTT assay. The results demonstrated that BTW2 exhibited distinct selectivity, with IC50 values of 1.18 ± 0.08 μM in MCF-7 cells and 20.79 ± 2.48 μM in HUVECs (Table S1), respectively. This 17.6-fold differential cytotoxicity of **BTW2** between cancer and normal cells, combined with its superior chemical stability (Fig. S32), indicates a favorable therapeutic window and reduced off-target effects, suggesting that **BTW2** warrants further investigation.

### Activation, tumor targeting, and metabolic stability of BTW2

To elucidate the activation mechanism of **BTW2**, we employed HPLC analysis to monitor its chemical transformation following ROS stimulation. ROS were generated by the Fenton reaction between H_2_O_2_ and Cu^2+^, which can produce highly reactive hydroxyl radicals (·OH) [46]. As shown in Fig. 3B to D, in the absence of ROS, the release of **W436** from **BTW2** is negligible (<2% cumulative release), which is beneficial to avoid side effects caused by the release of **W436** in normal tissues. Notably, more than 80.0% of **W436** was released from **BTW2** within 3 h in the presence of ROS. In contrast, minimal release of compound **W436** was observed from **BW** within the same timeframe, regardless of the presence of ROS. These findings conclusively establish the essentiality of both ROS presence and the TK-based responsive moiety for prodrug activation.

The effects of biotin on the antitumor activity of **BTW2** were tested to investigate the role of the biotin receptor in the transport of the prodrug. As shown in Fig. 3E, the cell viability of **BTW2** (1.0 μM) in the absence of biotin was only about 53.1%. When the cells were pretreated with biotin (1.5 μM), cell viability was increased to approximately 63.0%. These results indicate that excess biotin effectively blocks **BTW2** from binding to the biotin receptor, thereby preventing cellular uptake of **BTW2**. In contrast, the cell viability of **W436** (0.1 μM) was barely affected by biotin, indicating that the biotin receptor was not the primary factor in the uptake of **W436** by cells. In addition, biotin (1.5 μM) had no inhibitory effect on the growth of MCF-7 when applied alone, indicating that biotin had no effect on the function of the cells. These results confirmed that biotin-receptor-mediated endocytosis serves as the primary mechanism for **BTW2** internalization.

The study also examined the impact of NAC on **BTW2** (1.0 μM) cytotoxicity to further explore the role of ROS in **BTW2** release. When NAC (5.0 mM) was present, the cell viability of MCF-7 cells increased from 53.1% to 68.6%. Additionally, NAC (5.0 mM) exhibited minimal toxicity toward MCF-7 cells. Conversely, the cytotoxicity of **W436** (0.1 μM) did not show significant differences with or without NAC treatment, indicating that NAC does not influence the pharmacological effects of **W436** (Fig. 3E). The findings demonstrated that the activation of **BTW2** by ROS led to the release of **W436**.

In addition, the in vitro metabolic stability of **BTW2** was examined by measuring their half-lives upon incubation with mouse liver microsomes in the presence of an NADPH regeneration system. **BTW2** exhibited satisfying stability against mouse liver microsomes (*T*_1/2_ = 68.89 min and CL_int_ = 79.22 ml/min/kg) (Table S2).

### Validation of the dual-mechanism targeted cross-convergence prodrug activation strategy in vitro

**Fig. 4.**
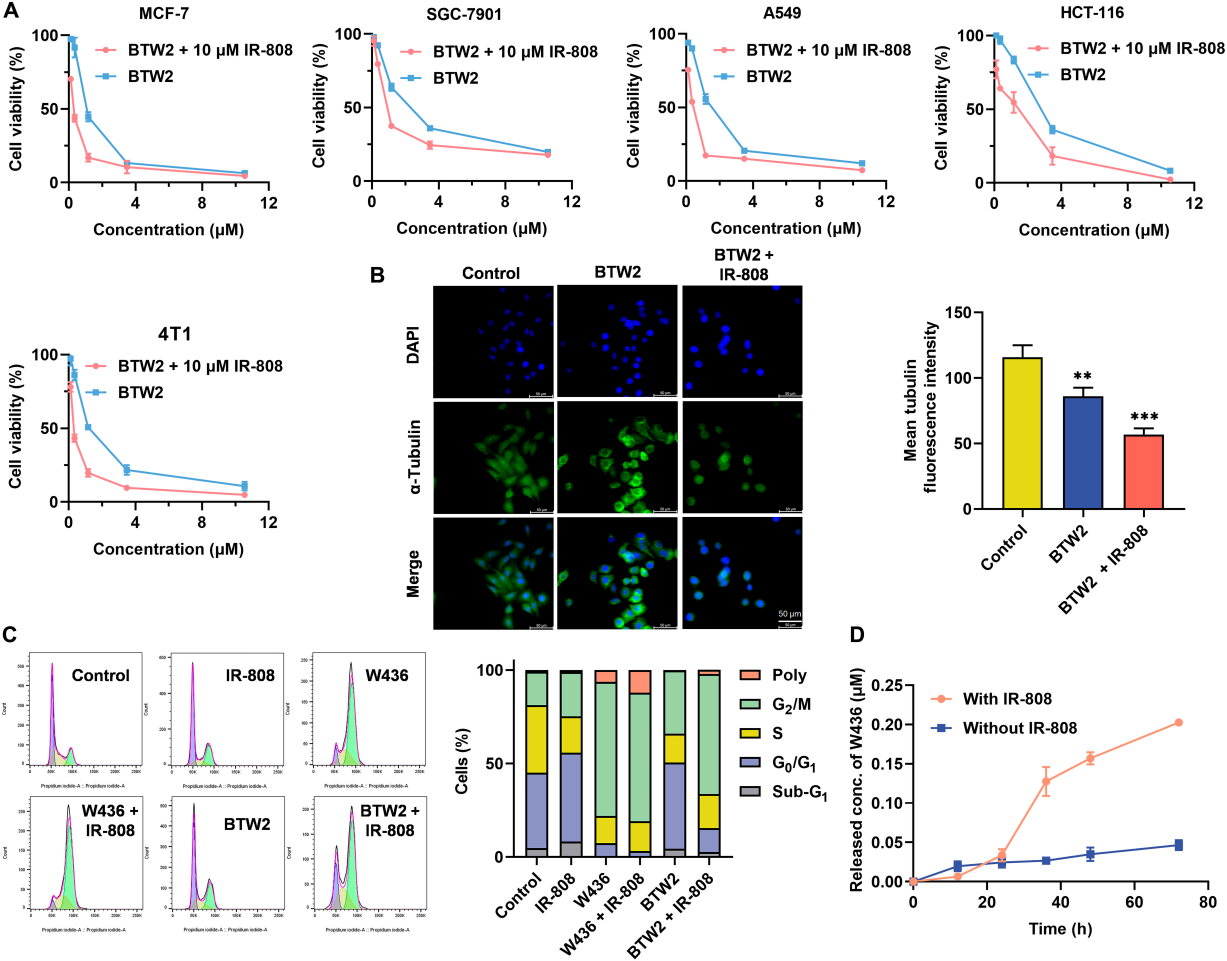
Validation of the cross-convergence prodrug activation strategy in vitro. (A) Dose-dependent cytotoxic effects of BTW2, either alone or in combination with IR-808, on 5 tumor cell lines. (*n* = 3). The MTT assay showed cell viability after treatment with various formulations for 72 h. (B) Different drug formulations interfered with microtubule polymerization in MCF-7 cells (0.5 μM, W436 equivalents; 10.0 μM, IR-808 equivalents). Microtubules are shown in green, and DNA is shown in blue. (C) The cell cycle of MCF-7 cancer cells after various treatments (0.5 μM, W436 equivalents; 10.0 μM, IR-808 equivalents). (D) Intracellular BTW2 release in MCF-7 cells treated with or without IR-808 (0.25 μM, W436 equivalents; 10.0 μM, IR-808 equivalents) (*n* = 3). Statistical significance: **P < 0.01 and ***P < 0.001 vs. control.

The antiproliferative effects of **BTW2** in combination with IR-808 were evaluated in 5 tumor cell lines using the MTT assay. The experimental results indicate that combination with IR-808 markedly enhanced the cytotoxic activity of **BTW2** across all tested cell lines. The most notable effect was observed in MCF-7 cells, where the IC_50_ value of **BTW2** decreased from 1.18 to 0.27 μM in combination with 10.0 μM IR-808, representing a 4.4-fold increase in potency. These findings strongly suggest that IR-808-mediated ROS generation plays a crucial role in in facilitating the activation of **BTW2** (Fig. 4A).

Subsequently, immunofluorescence staining experiments were performed to more intuitively observe the changes in microtubule morphology and distribution in MCF-7 cells treated with **BTW2** (0.5 μM) alone or in combination with IR-808 (10.0 μM). Microtubules in the cells of the control group were arranged in an orderly and uniform manner. Although the presence of multinucleated cells was observed in cells treated with **BTW2** alone, there was no obvious microtubule disruption. The cells in the combined group lost their normal shape, microtubules were destroyed, and it was difficult to observe the normal cytoskeleton (Fig. 4B).

In order to further confirm the ability of IR-808 to promote the activation of **BTW2**, we conducted an investigation into the impact of **BTW2** (with or without IR-808) on cell cycle progression. MCF-7 cells treated with various formulations for 36 h were subjected to flow cytometry analysis. Interestingly, while **BTW2** (0.5 μM) alone had minimal effect on cell cycle regulation, it caused a pronounced cell cycle arrest at the G_2_/M phase when combined with IR-808 (increasing from 33.7% to 64.0%). In contrast, there was little change in the cell cycle when **W436** was applied alone or in combination with IR-808. Furthermore, IR-808 itself did not affect the cell cycle (Fig. 4C).

Finally, we evaluated the concentration of released **W436** in MCF-7 cells. The results indicated no appreciable difference in the released concentration of **W436** within 24 h, with or without IR-808. However, at 36 h, the presence of IR-808 (10.0 μM) led to a notable increase in the released concentration of **W436**, reaching 0.13 μM, approximately 10 times higher than that without IR-808. By 72 h, almost all of the **W436** had been released from **BTW2** in the presence of IR-808 (Fig. 4D). The aforementioned experiments collectively demonstrated that IR-808 activates **BTW2** via ROS amplification to induce cytotoxicity while confirming the in vitro efficacy of this targeted prodrug activation strategy.

### Biodistribution and convergence of BTW2 NPs and IR-808 in vivo

The poor aqueous solubility of **BTW2** poses a considerable challenge for administration. To overcome this, we employed a nanomedicine approach that enhances prodrug solubility, enables tumor-targeting delivery via the EPR effect, and permits real-time in vivo tracking of NP biodistribution through fluorescent labeling. Accordingly, **BTW2** NPs were engineered via a one-step nanoprecipitation protocol. **BTW2** was found to self-assemble into NPs (**BTW2** NPs) in deionized water. DSPE-PEG2k (20% [w/w]) and lecithin (10% [w/w]) were added to increase the stability of the nanoassemblies. Dynamic light scattering was utilized to determine the particle size and zeta potential. The average diameter of **BTW2** NPs was around 133 nm, and the zeta potential was approximately −26 mV (Fig. S33). Because the prodrugs functioned as dual-acting agents (integrating self-delivery nanocarriers and intrinsic therapeutics), the **BTW2** NPs showed a higher drug-loading efficiency (23.9% [w/w]) than the commonly encapsulated nanoformulation (usually <10%) [47]. In addition, the **BTW2** NPs demonstrated favorable colloidal stability, as evidenced by minimal size and polydispersity index changes after 48-h incubation in serum at 37 °C (Fig. S34), and excellent long-term storage stability at 4 °C, with only modest variations over 14 d (Fig. S35).

**Fig. 5.**
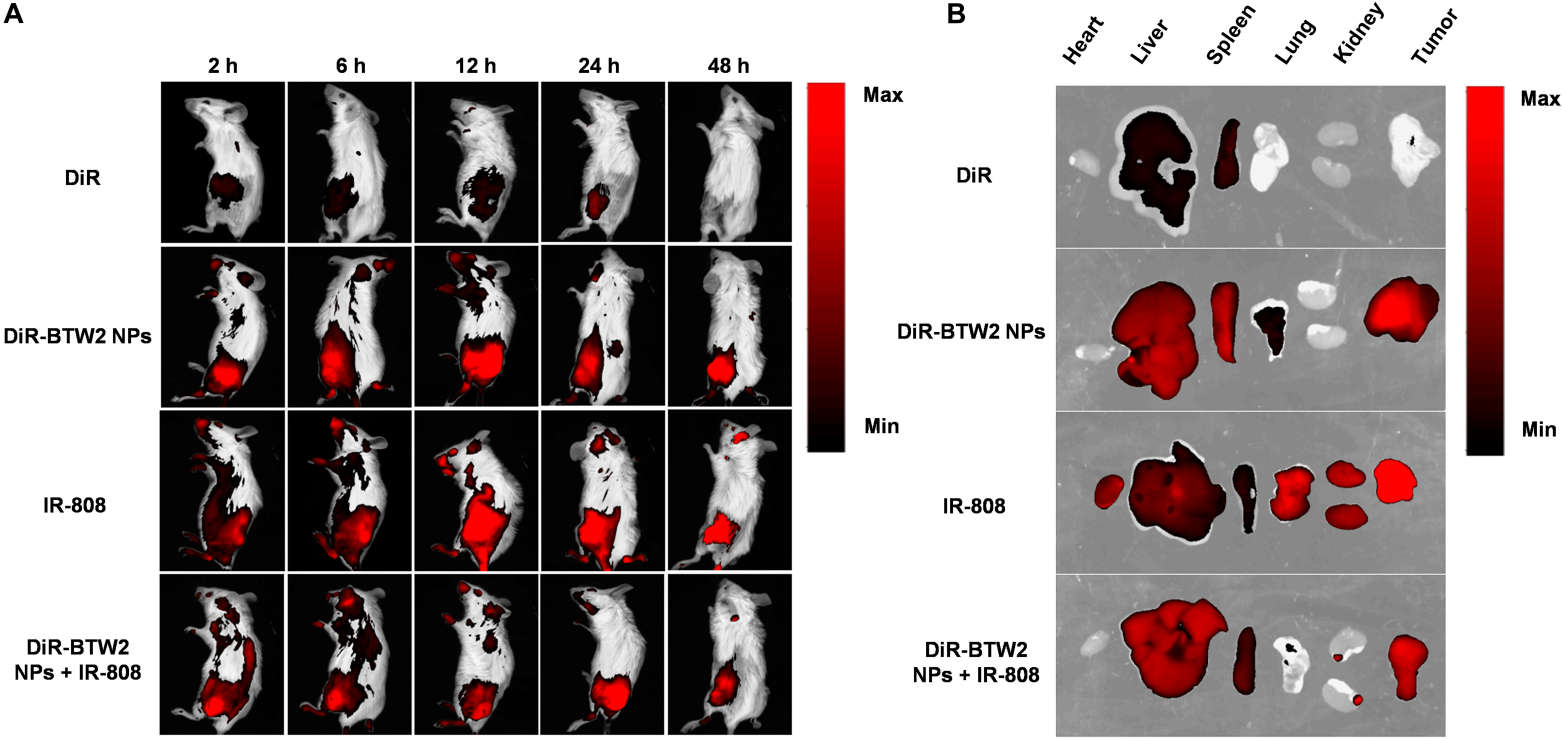
Biodistribution of different formulations in 4T1 tumor-bearing mice. (A) In vivo near-infrared (NIR) fluorescence images of the heterotopic 4T1 tumor-bearing mice at 2, 6, 12, 24, and 48 h after intravenous injection of free 1,1-dioctadecyl-3,3,3,3-tetramethylindotricarbocyanine iodide (DiR), DiR-BTW2 prodrug NPs, IR-808, or DiR-BTW2 NPs + IR-808 (1 mg kg^−1^, DiR equivalent; 1 mg kg^−1^, IR-808 equivalent). (B) NIR fluorescence images of dissected organs and xenograft tumor.

Next, we investigated the biodistribution and tumor accumulation of the **BTW2** NPs or IR-808 in heterotopic 4T1 tumor-bearing mice. The biodistribution of **BTW2** NPs was investigated by a fluorescence-labeling method. The DiR-labeled **BTW2** NPs (DiR-**BTW2** NPs) were prepared following the same protocol as described above for **BTW2** NPs except with the addition of DiR together with **BTW2** (**BTW2**:DiR = 10:1 w/w). After intravenous injection, free DiR was quickly eliminated from the body, with high fluorescence observed in the liver and spleen, but minimal fluorescence detected in the tumor. In contrast, after treatment with DiR-**BTW2** NPs, tumors exhibited clear and strong fluorescence signals, suggesting efficient accumulation of **BTW2** NPs at tumor sites due to the EPR effect. Similarly, 48 h post intraperitoneal injection of IR-808, tumors displayed bright fluorescence signals, indicating the tumor-targeting and long-acting accumulation properties of IR-808 (Fig. 5A and B and Fig. S36).

When IR-808 and DiR-**BTW2** NPs were cross-converged, a reduction in tumor fluorescence signal was observed, potentially attributed to the mutual influence of the 2 fluorescent dyes. To further validate this finding, we conducted in vitro experiments by mixing IR-808 and DiR solutions, which demonstrated consistent attenuation of fluorescence signals as observed in our in vivo studies (Fig. S37). This observation provides additional evidence supporting the spatiotemporal convergence of IR-808 and **BTW2** NPs at tumor sites.

### Antitumor effects of the dual-mechanism cross-convergence prodrug activation strategy in vivo

**Fig. 6.**
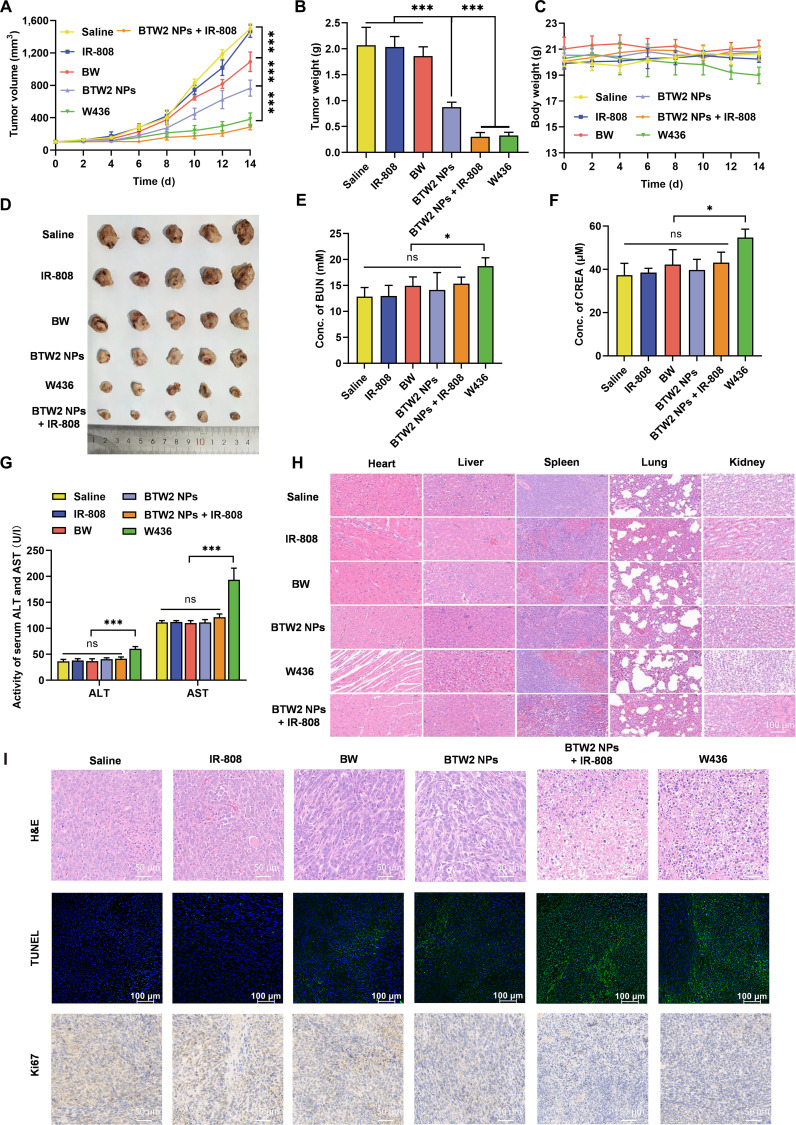
Antitumor effects of the cross-convergence prodrug activation strategy in vivo. (A) The tumor growth profiles and (B) tumor weights after various treatments (10.0 mg kg^−1^, IR-808 equivalent; 5.0 mg kg^−1^, W436 equivalent) (*n* = 5). (C) The body weight of mice changes upon treatment with different formulations (*n* = 5). (D) Tumor photos. (E and F) Hepatic function parameters of mice after treatments. (G) Renal function parameters of mice after treatments. (H) Hematoxylin and eosin (H&E) staining of major organs of heterotopic 4T1 tumor-bearing mice after being treated with different formulations. (I) H&E, terminal deoxynucleotidyl transferase dUTP nick end labeling (TUNEL), and Ki67 staining of tumors after mice received treatments. Statistical significance: *P < 0.05 and ***P < 0.001; ns, not significant (*P* ≥ 0.05). BUN, blood urea nitrogen; CREA, creatinine; ALT, alanine aminotransferase; AST, aspartate aminotransferase.

After successfully demonstrating the spatiotemporal convergence of IR-808 and **BTW2** NPs at the tumor site, the in vivo tumor inhibition effects were studied using heterotopic 4T1 tumor-bearing mice. With the exception of IR-808, which was administered via intraperitoneal injection, the remaining agents were administered through tail vein injection. As illustrated in Fig. 6A and B, the saline and IR-808 groups experienced rapid tumor growth, and tumor growth was slightly inhibited in the **BW** group with an inhibition rate of 10.1%. Although **BTW2** NPs were able to accumulate at tumor sites, the tumor inhibition effect in this group was not ideal, with a tumor inhibition rate of only 53.2%, which was potentially due to insufficient ROS levels. Notably, the combination of **BTW2** NPs with IR-808 resulted in an increased tumor inhibition rate of 85.4%, even more than that of parent drug **W436** (82.4%) due to the elevated level of ROS induced by IR-808 in tumor cells. H&E staining of tumor tissues from the **BTW2** NPs + IR-808 group revealed pronounced treatment-induced alterations, characterized by reduced cellularity in viable regions and degenerative features in the remaining cells, including cytoplasmic vacuolization and nuclear pyknosis. Correspondingly, the TUNEL assay demonstrated markedly increased apoptosis in this combination group, as evidenced by enhanced green fluorescence compared to that of all control groups. Consistent with this pro-apoptotic effect, immunohistochemical analysis further showed a marked down-regulation of Ki67 protein expression, confirming the suppression of tumor cell proliferation (Fig. 6I).

Body weight changes were monitored to assess treatment safety. While other groups remained stable, mice receiving **W436** showed notable weight loss during treatment (Fig. 6C), indicating potential systemic toxicity. Consistent with this, serum analysis revealed elevated levels of renal biomarkers (blood urea nitrogen and creatinine) and hepatic enzymes (alanine aminotransferase and aspartate aminotransferase) in the **W436** group versus the saline group, confirming nephro- and hepatotoxicity. In contrast, the **BTW2** NPs and **BTW2** NPs + IR-808 groups showed no such elevations (Fig. 6E to G). Histopathological evaluation by H&E staining further supported these findings: **W436** caused severe damage to the heart (myocardial necrosis) and liver (hepatocyte vacuolization), whereas **BTW2**-based treatments induced no observable alterations in major organs (Fig. 6H). Collectively, the cross-convergence prodrug activation strategy achieved potent antitumor efficacy while minimizing systemic toxicity in vivo.

### Antimetastatic effects of the dual-mechanism cross-convergence prodrug activation strategy in vivo

**Fig. 7.**
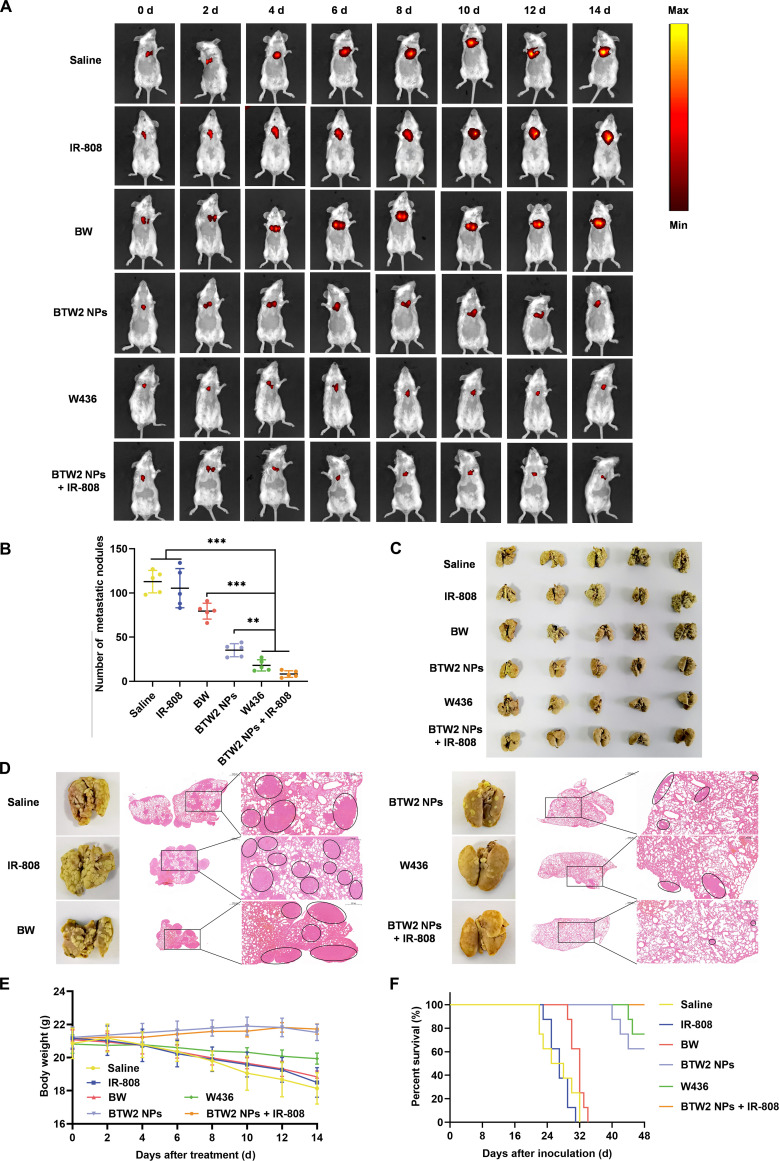
Antimetastatic effects of the cross-convergence prodrug activation strategy in vivo. (A) Bioluminescence images of lung metastatic tumor growth in mice during treatment. (B) The number of surface lung metastases (10.0 mg kg^−1^, IR-808 equivalent; 5.0 mg kg^−1^, W436 equivalent) (*n* = 5). (C) Changes in body weight of mice during treatment of the lung metastasis model (*n* = 5). (D) Images of whole lungs of mice. (E) H&E staining of the lungs. (F) Survival ratios (%) of mice with lung metastasis of breast cancer after various treatments. Statistical significance: **P < 0.01 and ***P < 0.001.

Distant metastasis is a common feature of malignant tumors and is a major cause of refractory tumors [48]. Therefore, we investigated the antimetastatic effects of the cross-convergence prodrug activation strategy. Given that 4T1-Luc cells can spontaneously induce the development of highly metastatic breast cancer when injected into BALB/c mice, we employed tail vein injection of 4T1 cells to establish a model for tumor blood metastasis. During the treatment, lung metastatic tumor growth was monitored at each time point using bioluminescence imaging. Metastatic lesions progressed markedly in the saline, IR-808, and **BW** groups; while **BTW2** NPs modestly slowed tumor progression, a gradual deterioration was still observed. In contrast, metastatic growth was effectively suppressed in both the **W436** and **BTW2** + IR-808 groups (Fig. 7A). The experimental results show that IR-808 and **BW** barely reduced the number of nodules in the lungs. **BTW2** NPs slightly reduced the number of metastatic nodules, with an average number of 38 nodules per lung. When **BTW2** NPs were combined with IR-808, the average number of metastatic nodules per lung was further reduced to 7, which was only 6.0% of that of the saline group (Fig. 7B and C). We further analyzed metastatic nodules in H&E-stained lungs harvested from mice, which were identified as cell clusters with darkly stained nuclei (Fig. 7D). Metastatic foci were clearly detectable in the saline, IR-808, and **BW** groups, indicating that the combination of **BTW2** NPs and IR-808 had an obvious antimetastatic ability.

In terms of body weight, the mice in the saline group, IR-808 group, and **BW** group exhibited pronounced weight loss, which may be associated with the exacerbation of lung metastasis. The mice treated with **W436** experienced a slight weight loss, potentially attributable to the toxicity of **W436** itself. In contrast, mice treated with **BTW2** NPs and **BTW2** NPs + IR-808 displayed a stable increase in body weight, indicating good biosafety (Fig. 7E).

The ultimate goal of chemotherapy is to enhance the QOL and extend the survival of cancer survivors [49]. Therefore, the mice’s survival rate in each group was recorded and utilized as parameters for evaluating QOL. Mice in the saline, IR-808, and **BW** groups began to die around the 24th day after modeling, and the mice completely died around the 32nd day, indicating severe damage to the body by the tumor cells. Mice treated with **BTW2** NPs and **W436** also experienced a small number of deaths during the experiment. It is worth noting that no mice died in the **BTW2** NPs + IR-808 group during the 46-d experiment (Fig. 7F), which markedly prolonged the life of mice with tumor metastasis, suggesting that **BTW2** NPs + IR-808 had the lowest physical toxicity and the best effect in the treatment of tumor metastasis. The above experimental results have convincingly demonstrated the viability of the cross-convergence prodrug activation strategy for the treatment of metastatic tumors.

## Conclusion

**Fig. 8.**
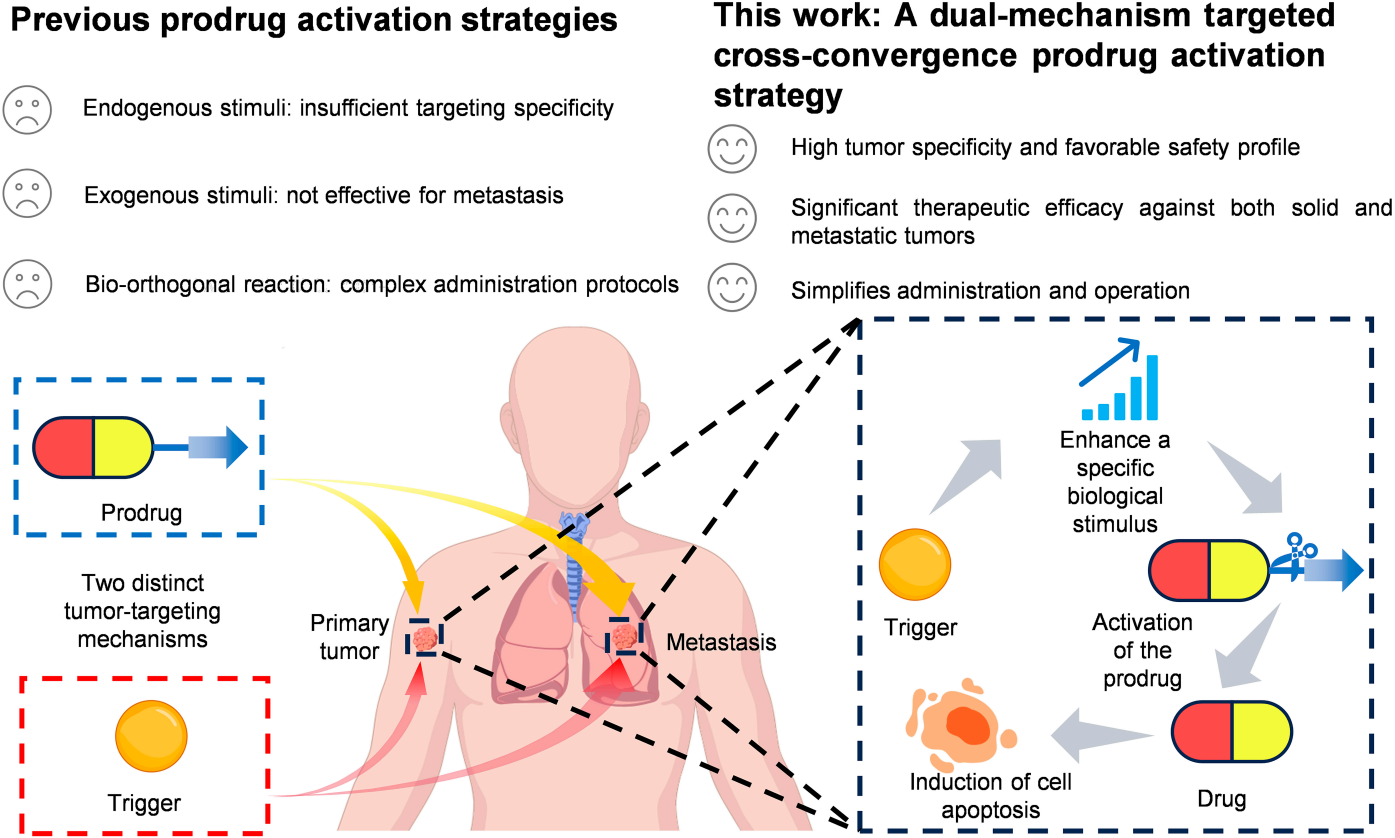
Schematic illustration of the dual-mechanism targeted cross-convergence prodrug activation strategy.

In summary, our study introduces a groundbreaking dual-mechanism targeted cross-convergence prodrug activation platform that synergistically integrates tumor-targeting stimulus amplification with self-reinforcing drug release, offering a novel paradigm for precision cancer therapy (Fig. 8). The cornerstone of our strategy lies in the orchestrated interplay between 2 key components: (a) **BTW2** NPs, a ROS-responsive prodrug engineered for dual tumor accumulation via the EPR effect and biotin-receptor-mediated targeting, and (b) IR-808, a heptamethine dye that selectively enriches in tumors through organic-anion-transporting polypeptides and amplifies intracellular ROS. This design ensures that prodrug activation is confined to tumor sites, thereby minimizing off-target toxicity and enhancing therapeutic precision. The activation of **BTW2** NPs is initiated by IR-808, which elevates tumor ROS levels by 7.7-fold, triggering the release of the potent tubulin inhibitor **W436**. Once released, **W436** exerts its therapeutic effect by inhibiting tubulin activity and further amplifies intracellular ROS levels, forming a self-reinforcing positive feedback loop to enhance therapeutic specificity. The strategy exhibited remarkable efficacy in both localized and metastatic tumor models, achieving an 85.4% inhibition rate in 4T1 subcutaneous tumors and reducing pulmonary nodules by 94.0% in a metastatic lung model while maintaining 100% survival at 46 d compared to a median survival of 25 d in the saline group. These findings underscore our strategy’s ability to address unmet needs in metastatic cancer therapy, effectively overcoming the limitations of current approaches.

## Data Availability

All data that support the findings of this study are available from the corresponding authors upon reasonable request.

## Supplementary Materials

Supplementary 1 Schemes S1 to S3 Figs. S1 to S37 Tables S1 and S2
