# Supplementary material for: A Dual-Mechanism Targeted Cross-Convergence Prodrug Activation Strategy Enabled by Biological Stimulus Amplification toward Precision Cancer Therapy
Source: Biomater Res. 2026 May 20;30:0328. doi: 10.34133/bmr.0328 (PMC13187504; doi:10.34133/bmr.0328)
Supplement: Supplementary 1 — Schemes S1 to S3 Figs. S1 to S37 Tables S1 and S2 [file bmr.0328.f1.docx]

Supplementary Information

A Dual-mechanism Targeted Cross-convergence Prodrug Activation Strategy Enabled by Biological Stimulus Amplification towards Precision Cancer Therapy

Zeyu Wang, Yajie Xing, Dong Wang, Yumeng Niu, Fang Chen, Kun Fan, Xin Meng, Mi Li, Dandan Xia, Lan Wu, Haiyan Xu, Qi Guan, Weige Zhang

**Contents**

Schemes S1 to S3

Figs. S1 to S37

Tables S1 to S2

**Scheme S1.** Reagents and conditions: (a) MeOH, H_2_SO_4_, reflux, 6 h; (b) CH_3_CN, NaH (70%), THF, reflux, 3 h; (c) NaOAc, benzene diazonium chloride, EtOH, 0℃, 1 h; (d) 4-*N*-Boc-Aminopiperidine, Cu(OAc)_2_, Air, CH_3_CN, reflux, 5 h; (e) TFA/CH_2_Cl_2_, r.t., 30 min.

**Scheme S2.** Reagents and conditions: (a) Thioglycolic acid, r.t., neat, 6 h; (b) NaBH_4_, I_2_, THF, reflux, 24 h; (c) 4-nitrophenyl carbonochloridate, Et_3_N, THF, 0℃, 8 h; (d) **13**, THF, r.t., 12 h; (e) Biotin, DCC, DMAP, r.t., 24 h.

**Scheme S3.** Reagents and conditions: (a) HATU, DMF, DIPEA, 40℃, 8 h.


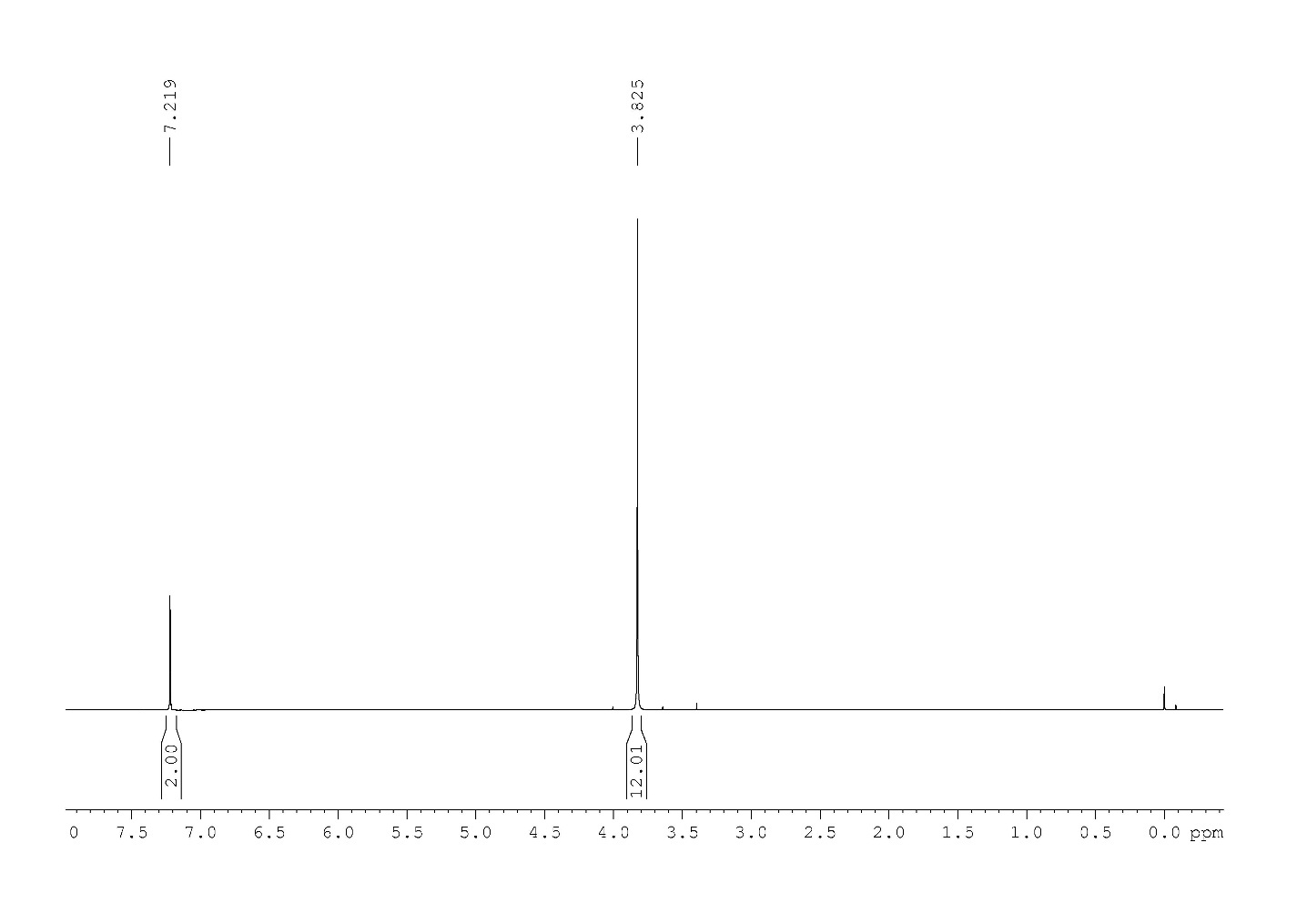

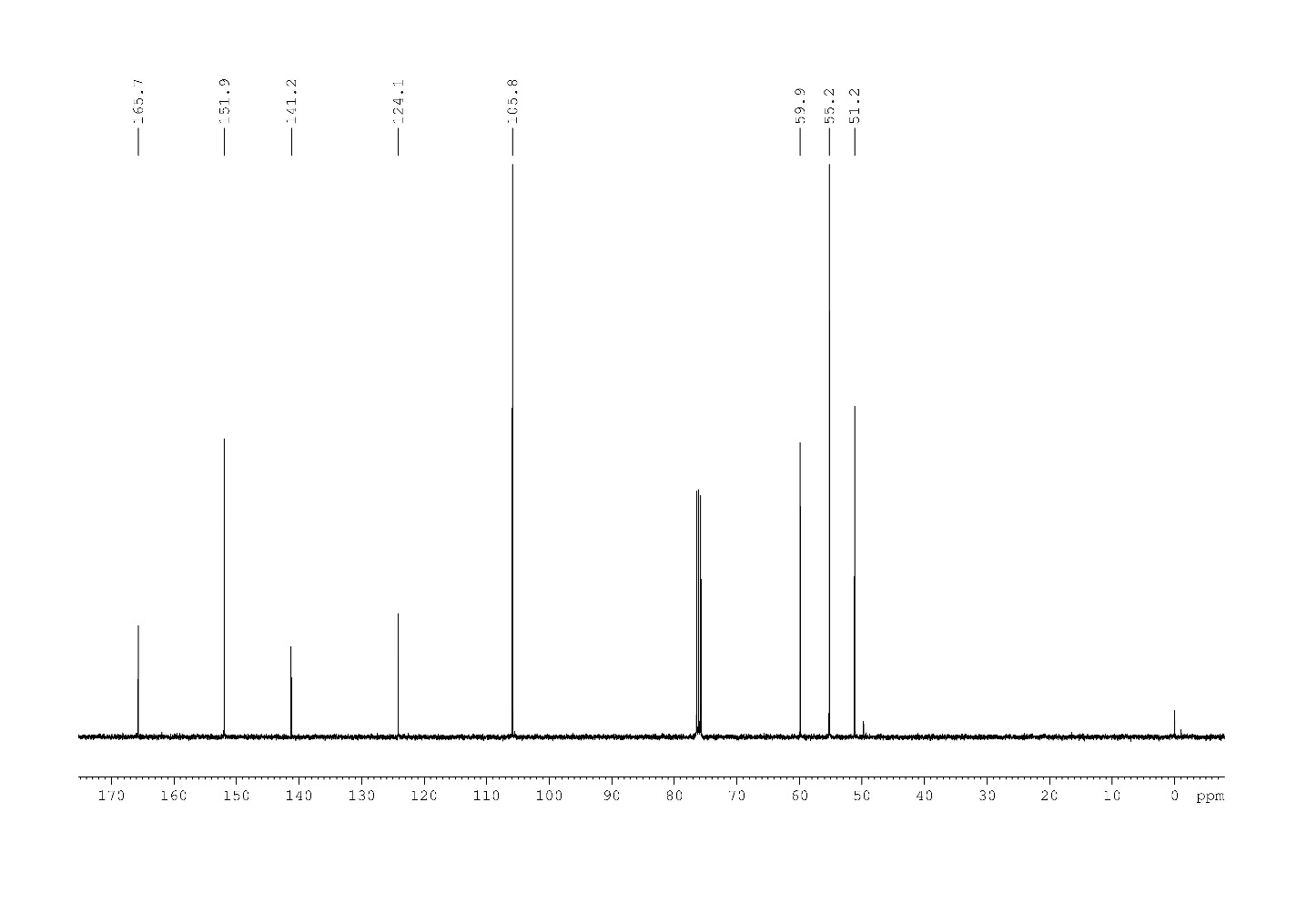

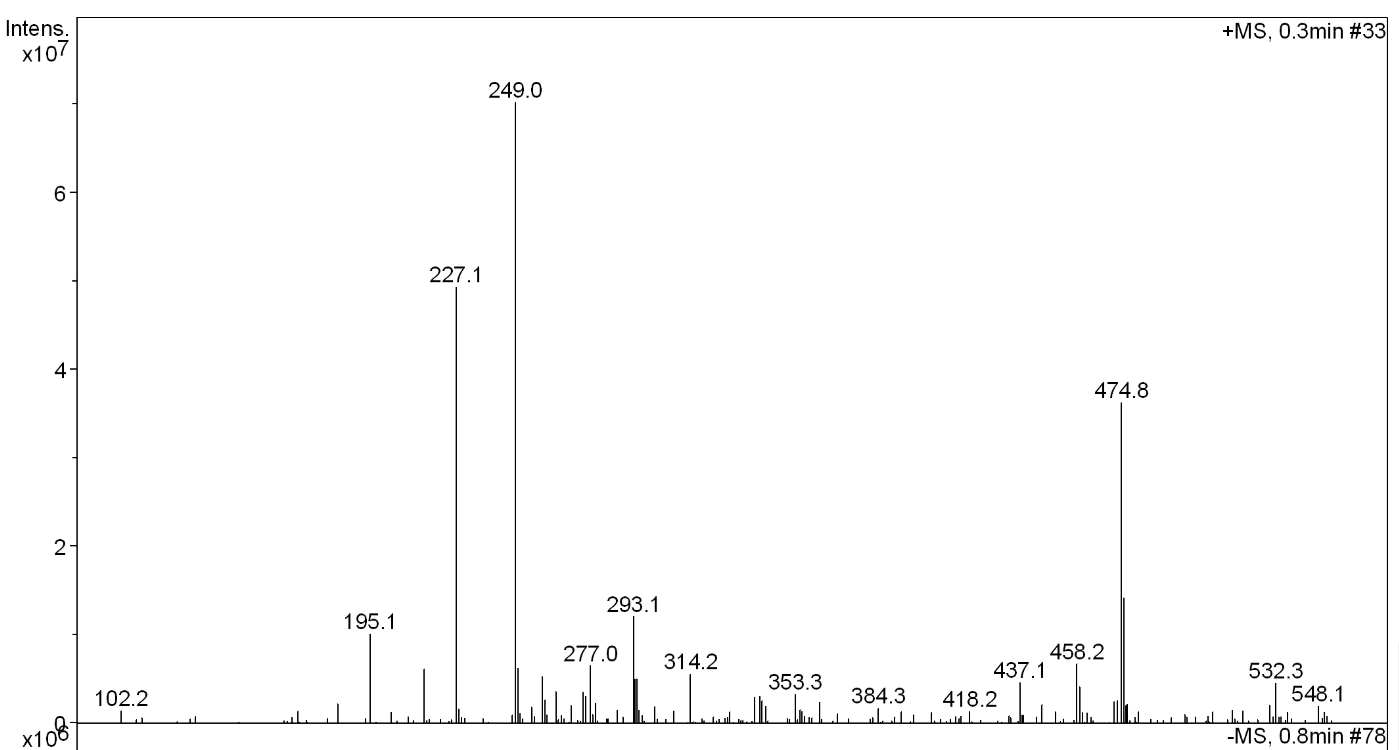


[M+Na]^+^

[M+H]^+^

**Fig. S1.** ¹H NMR spectrum (CDCl₃), ¹³C NMR spectrum (CDCl₃), and MS spectrum (ESI) of compound **2**.


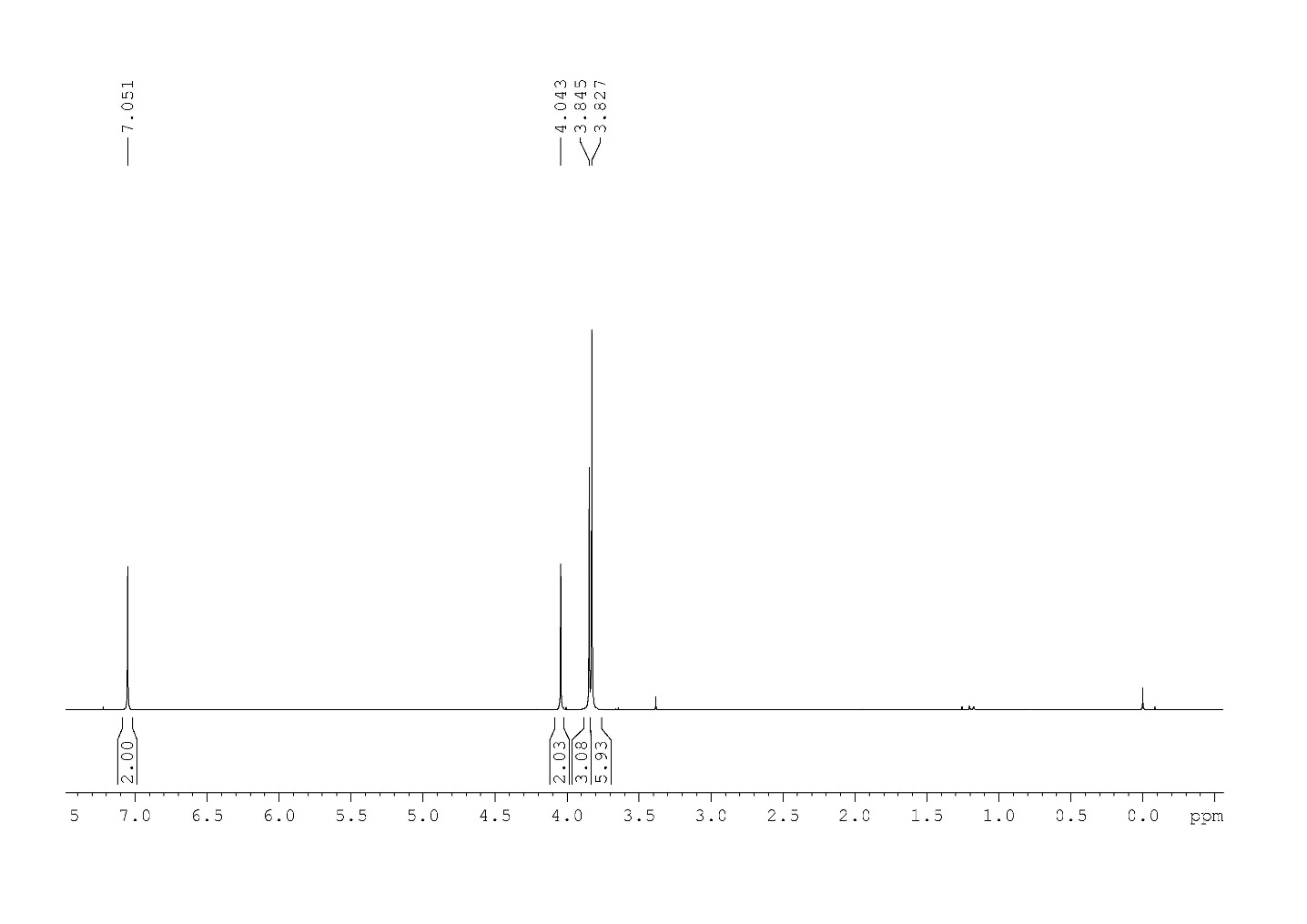

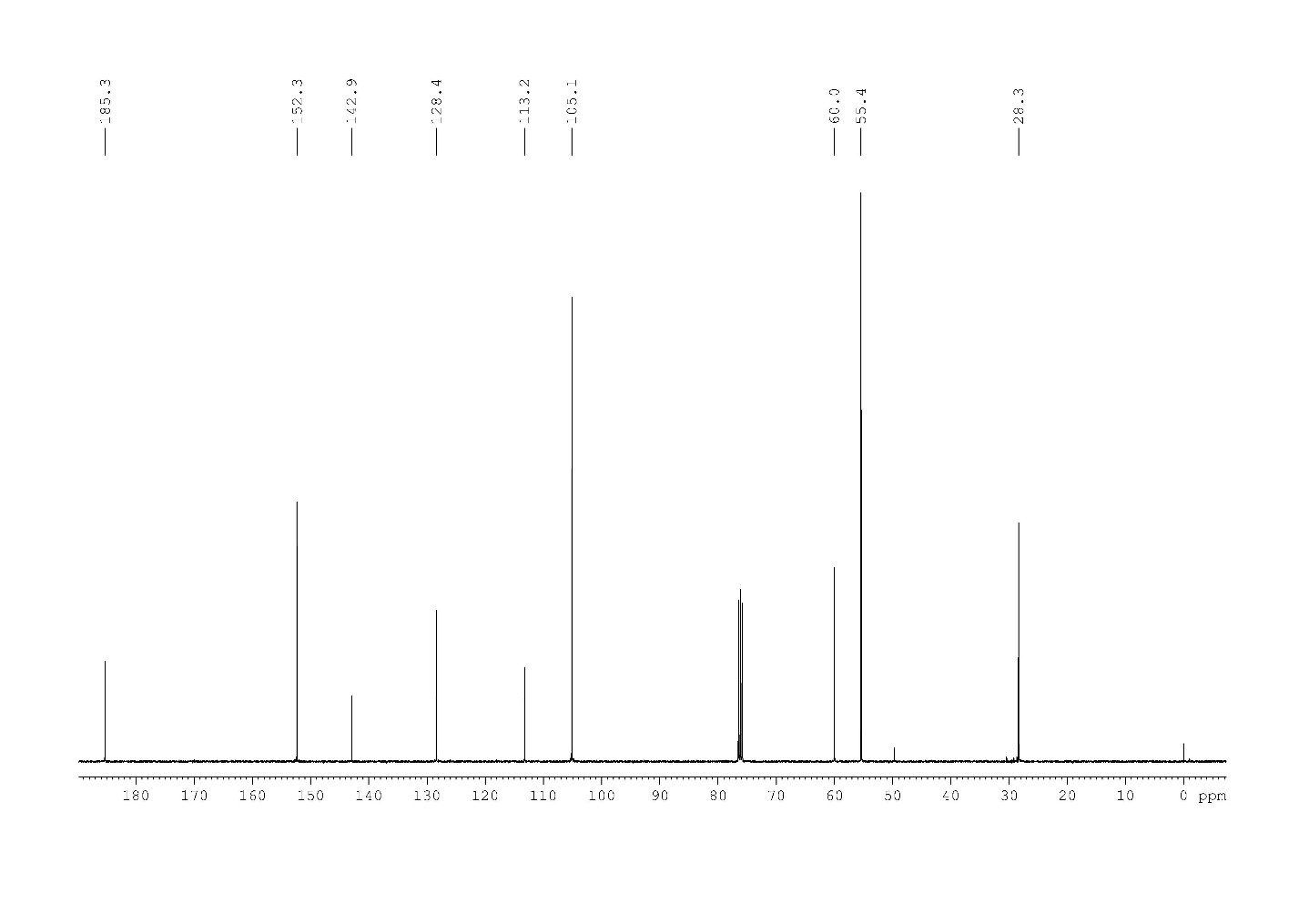

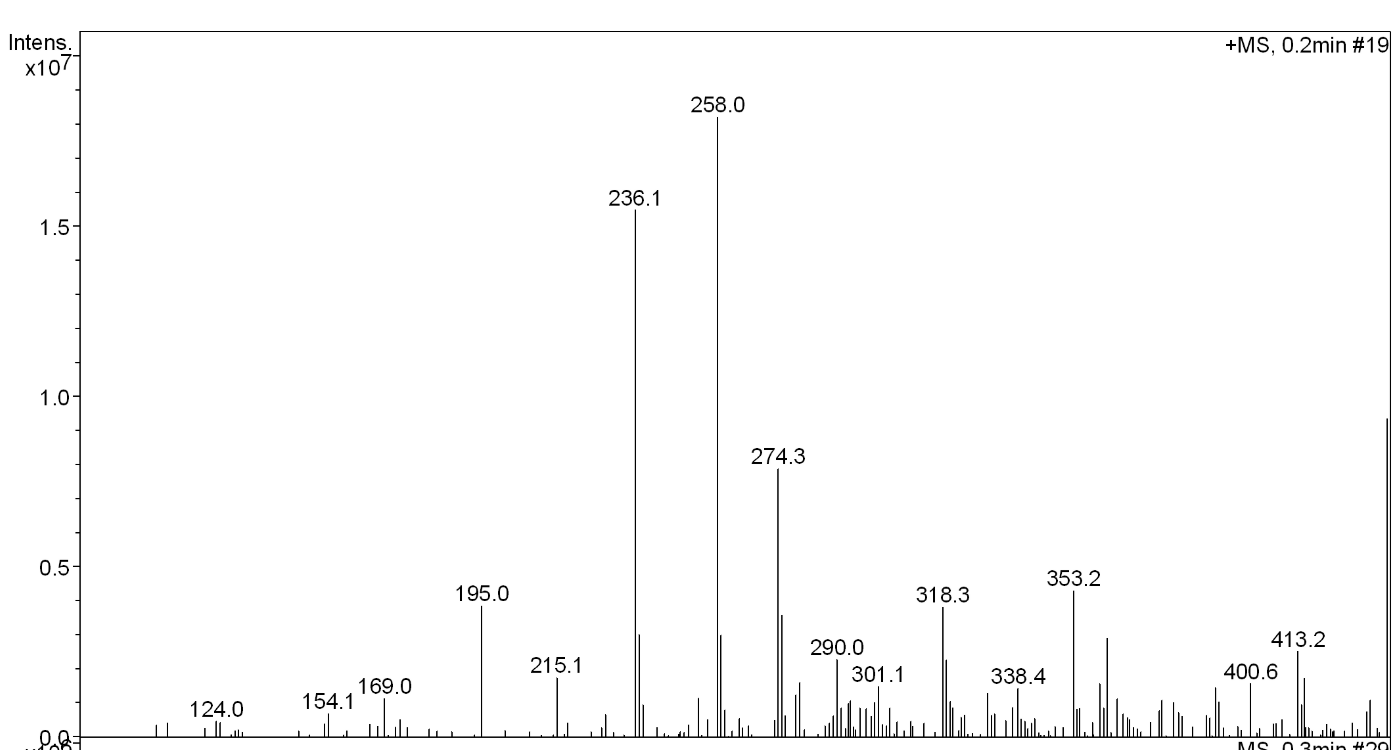


[M+H]^+^

[M+Na]^+^

**Fig. S2.** ¹H NMR spectrum (CDCl₃), ¹³C NMR spectrum (CDCl₃), and MS spectrum (ESI) of compound **3**.


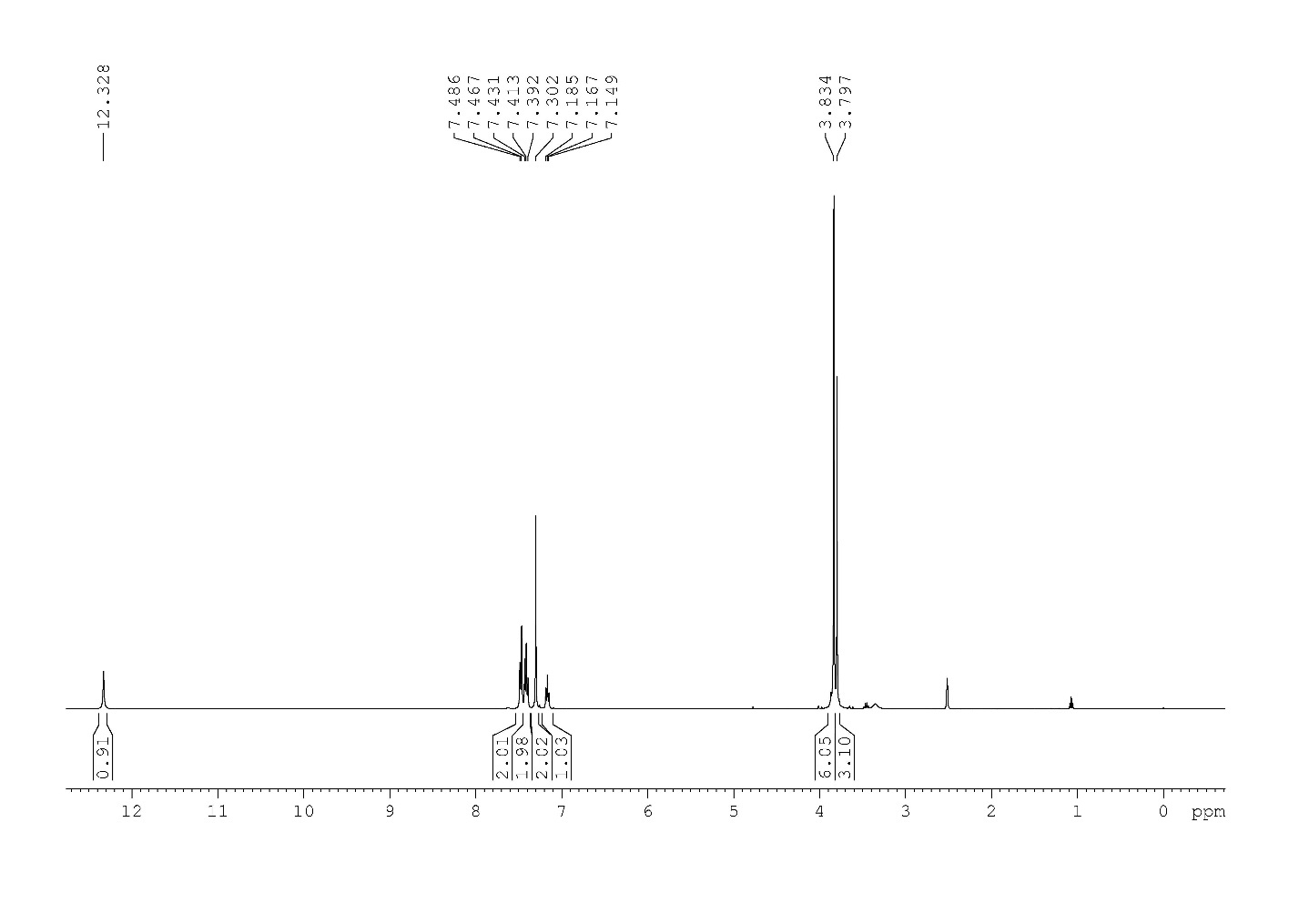

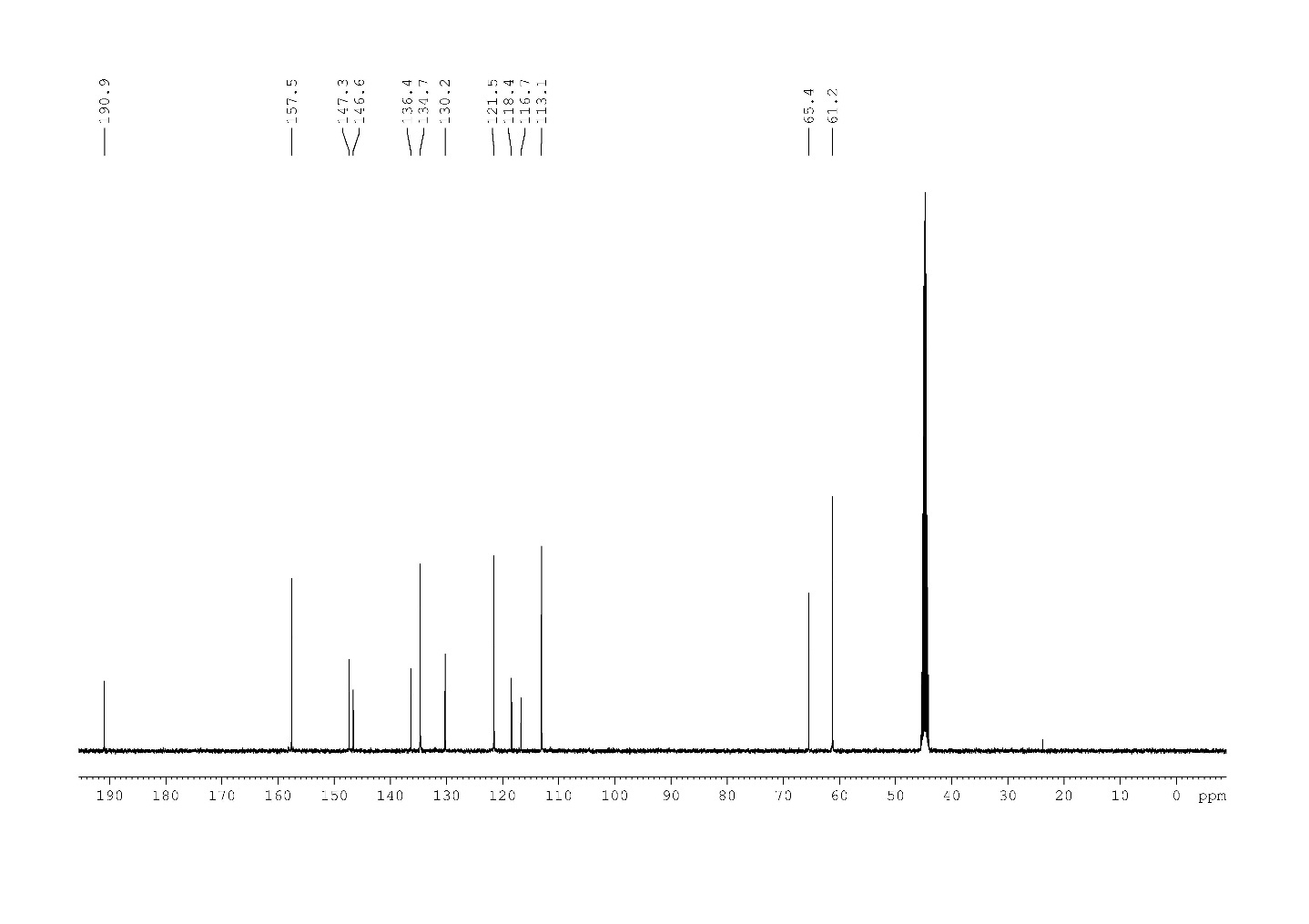

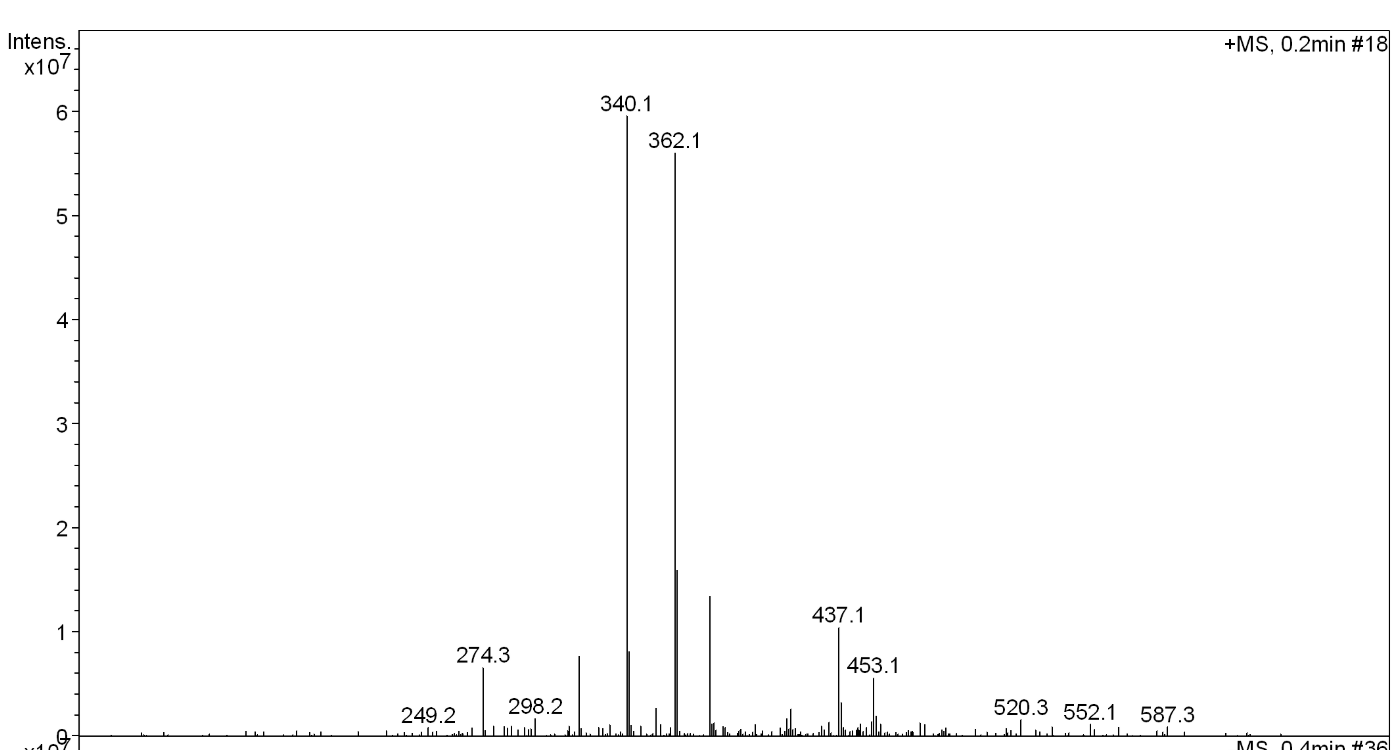


[M+Na]^+^

[M+H]^+^

**Fig. S3.** ¹H NMR spectrum (CDCl₃), ¹³C NMR spectrum (CDCl₃), and MS spectrum (ESI) of compound **4**.


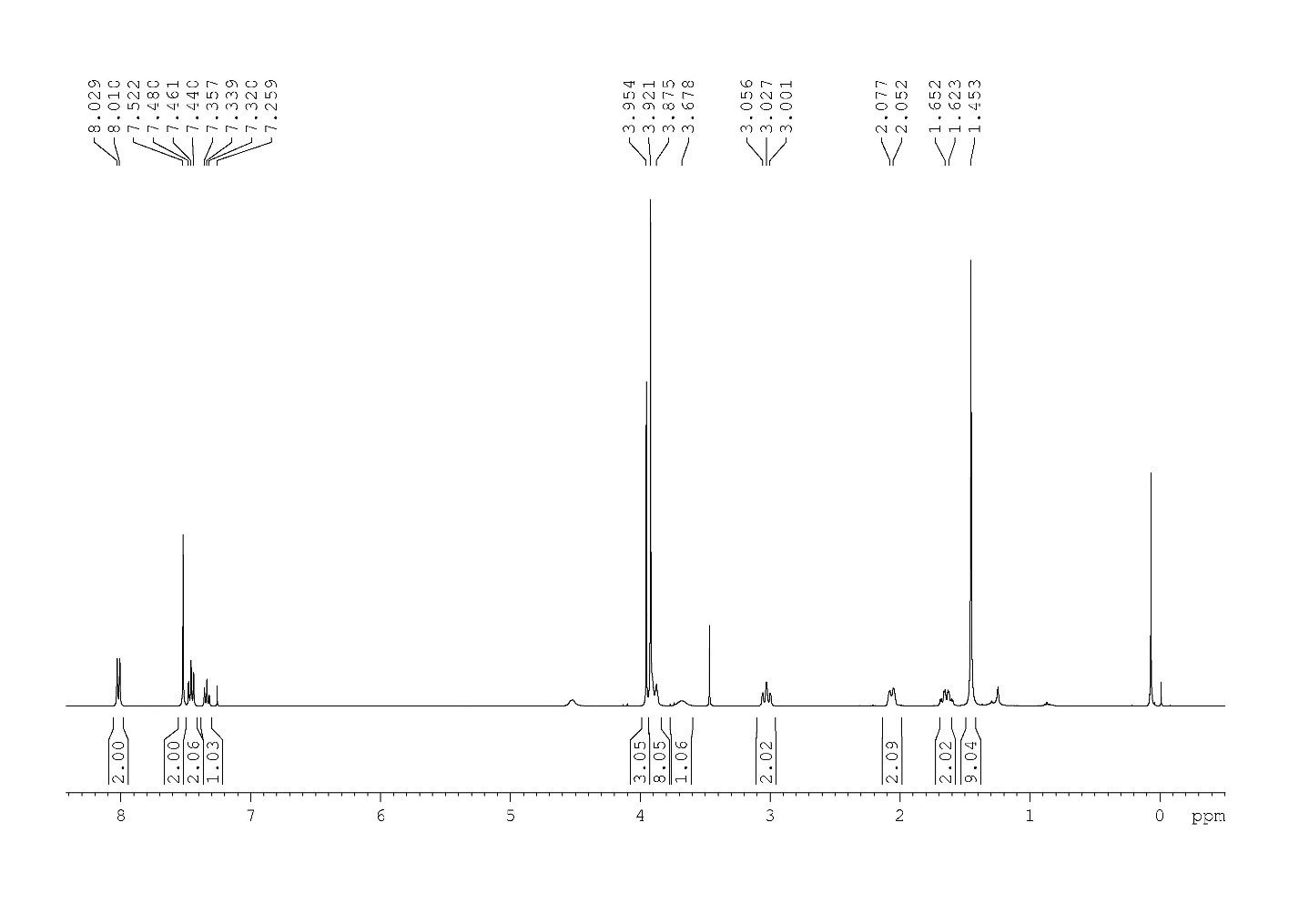

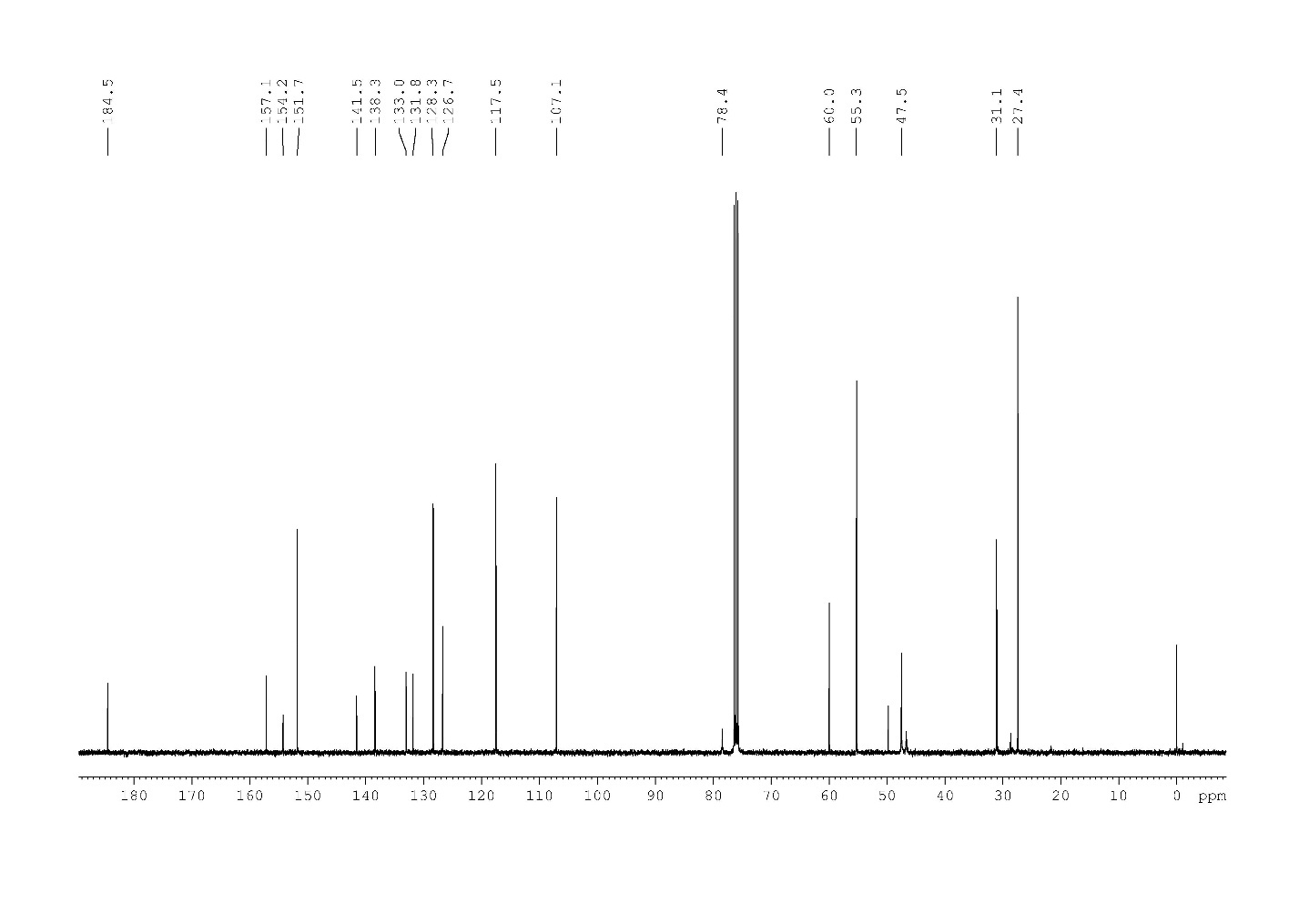

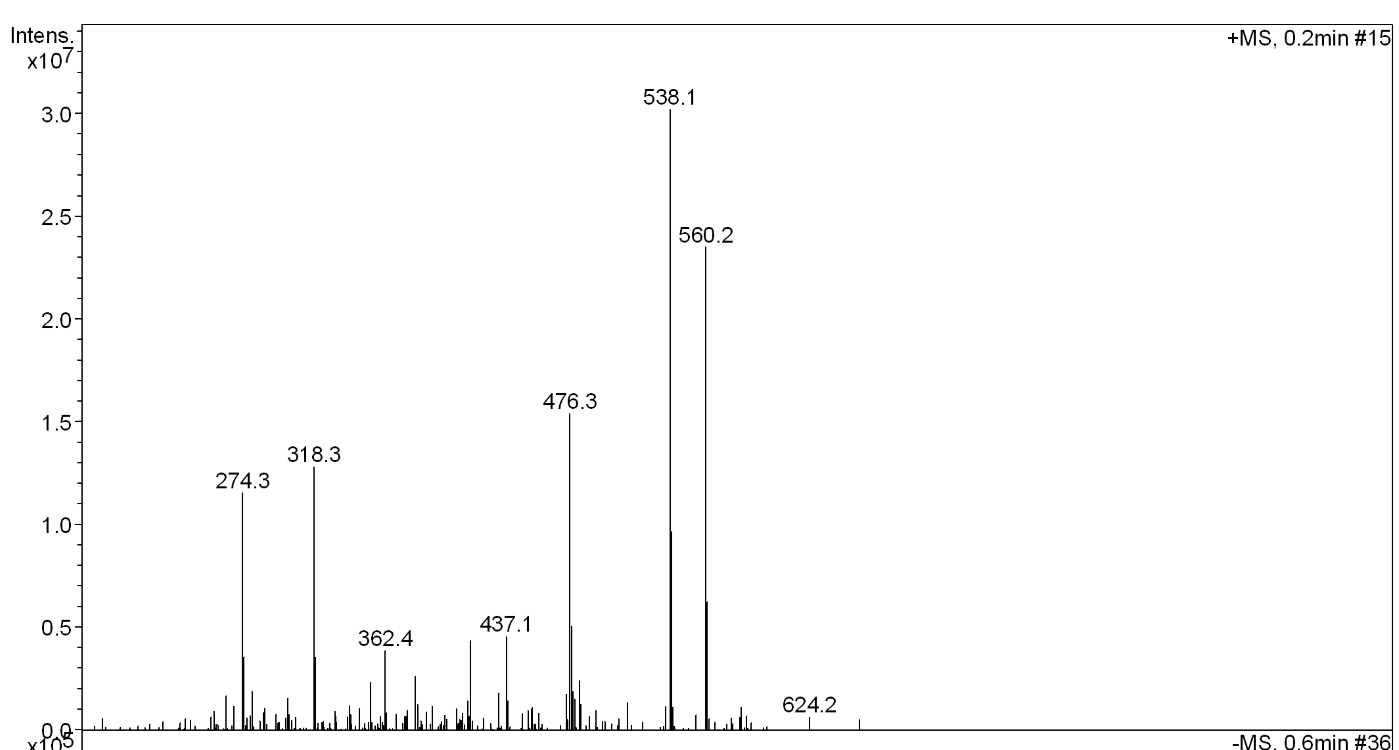


[M+H]^+^

[M+Na]^+^

**Fig. S4.** ¹H NMR spectrum (CDCl₃), ¹³C NMR spectrum (CDCl₃), and MS spectrum (ESI) of compound **5**.


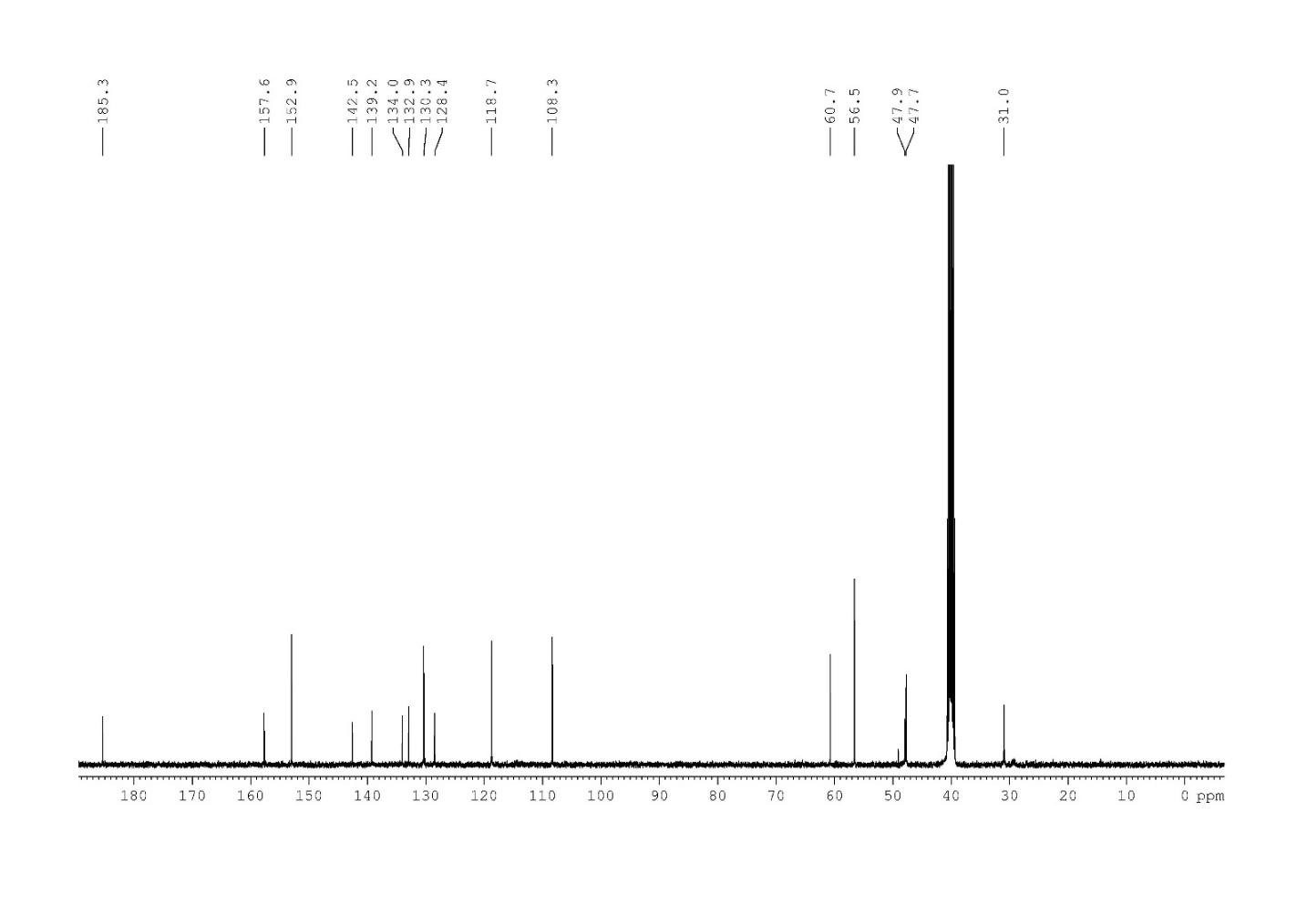

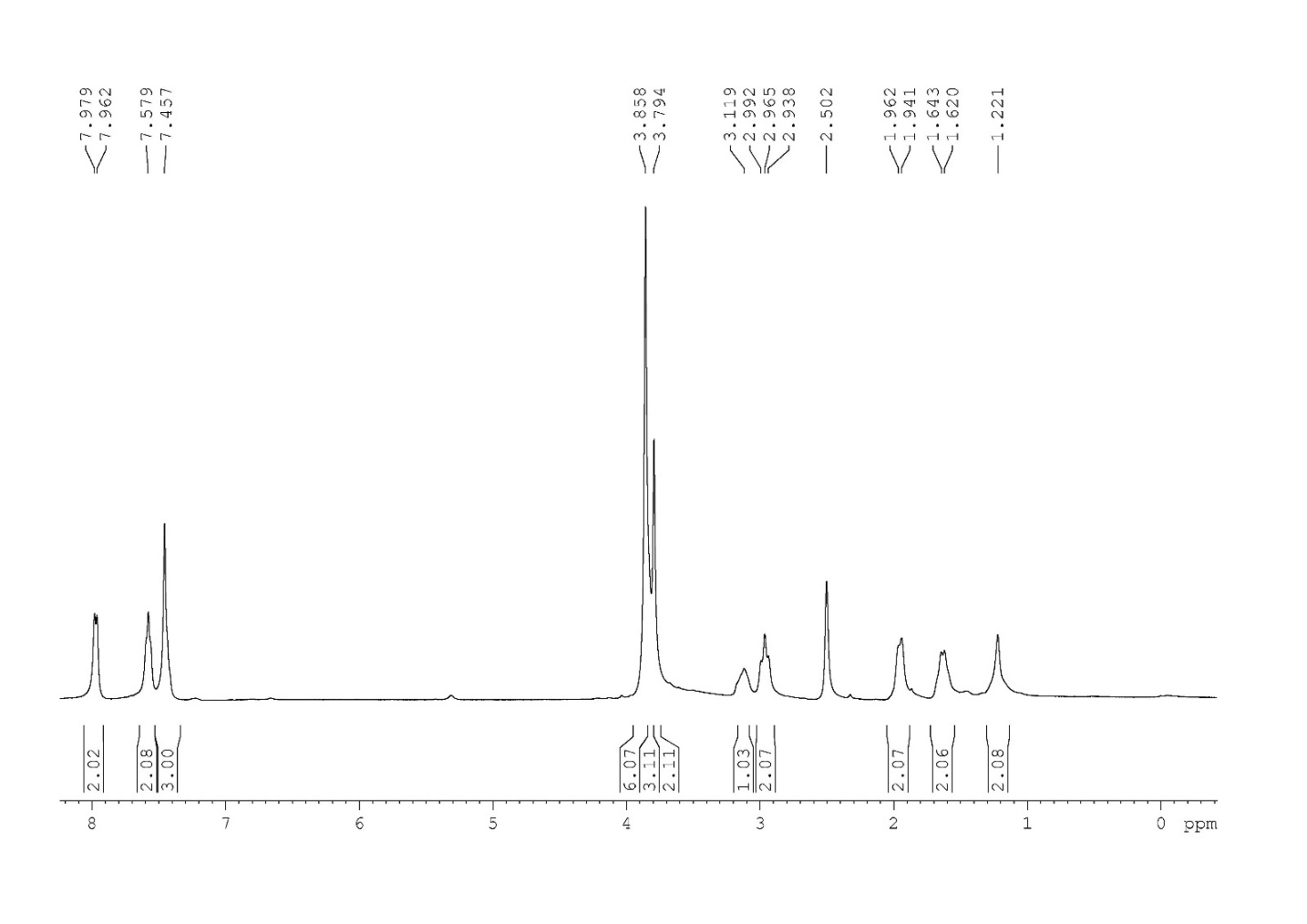


**
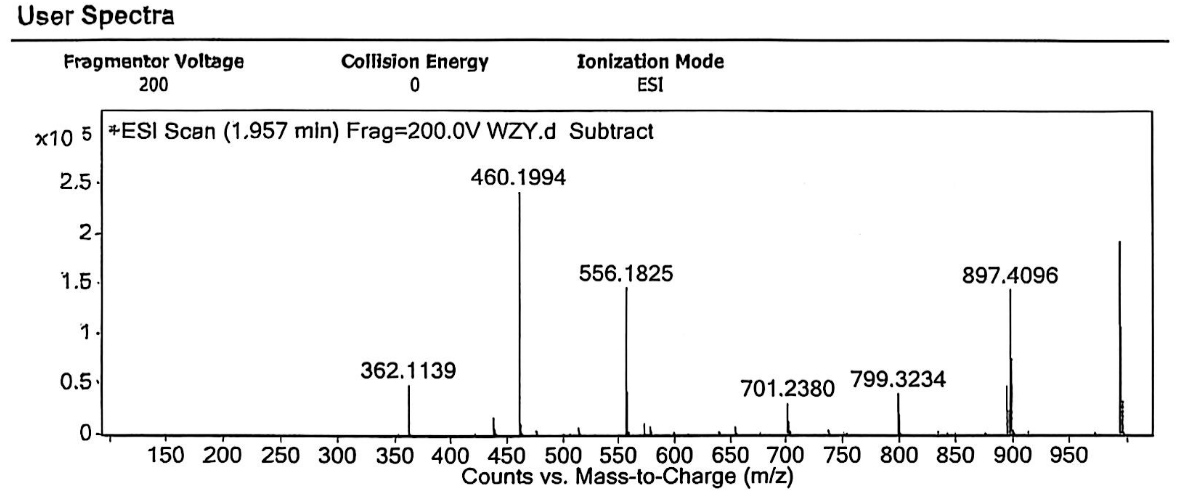
Fig. S5.** ¹H NMR spectrum (DMSO-*d*_6_), ¹³C NMR spectrum (DMSO-*d*_6_), and HRMS spectrum (ESI) of **W436**.

[M+Na]^+^

**
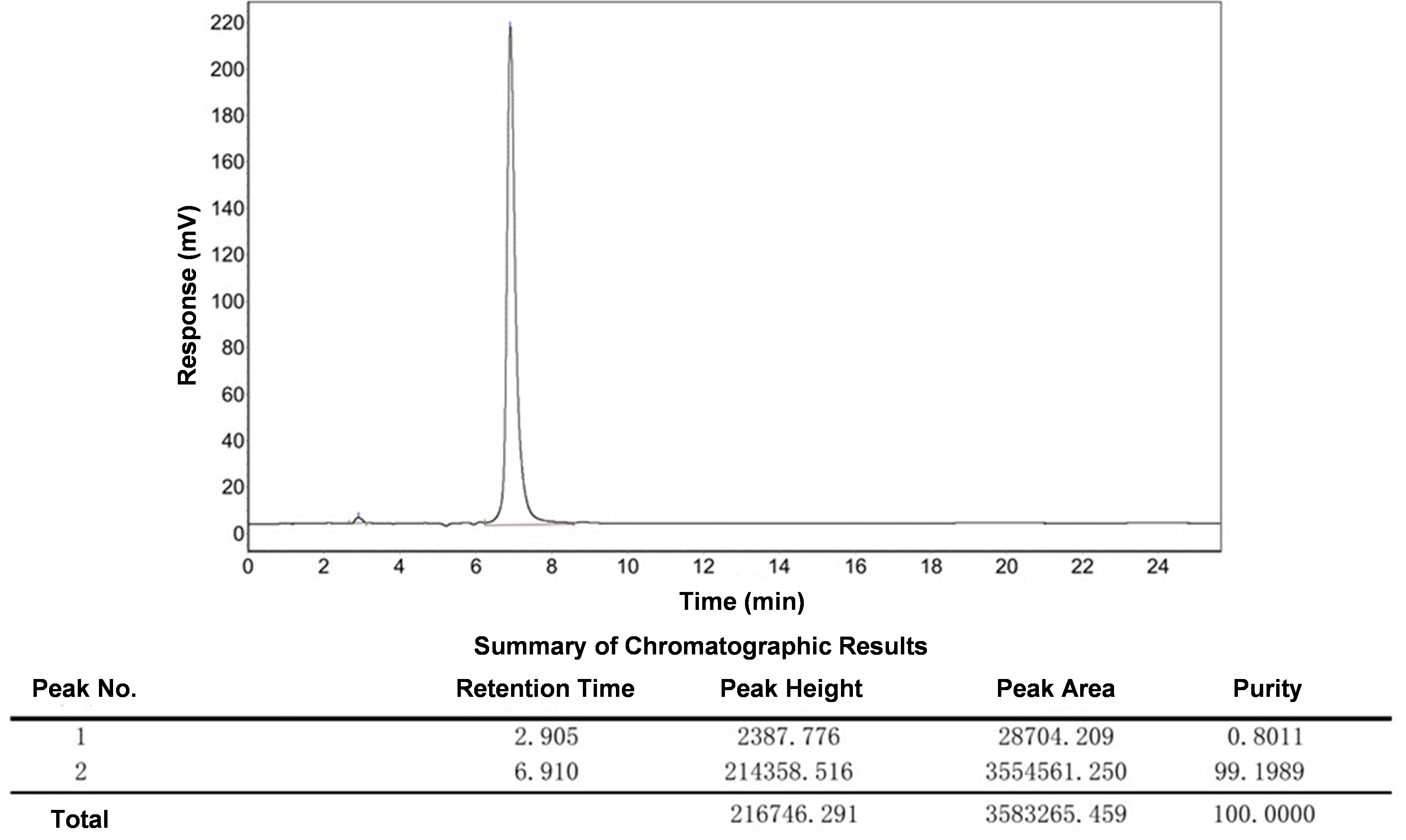
**

**Fig. S6.** HPLC chromatograms of **W436** for purity analysis (detection at 254 nm).


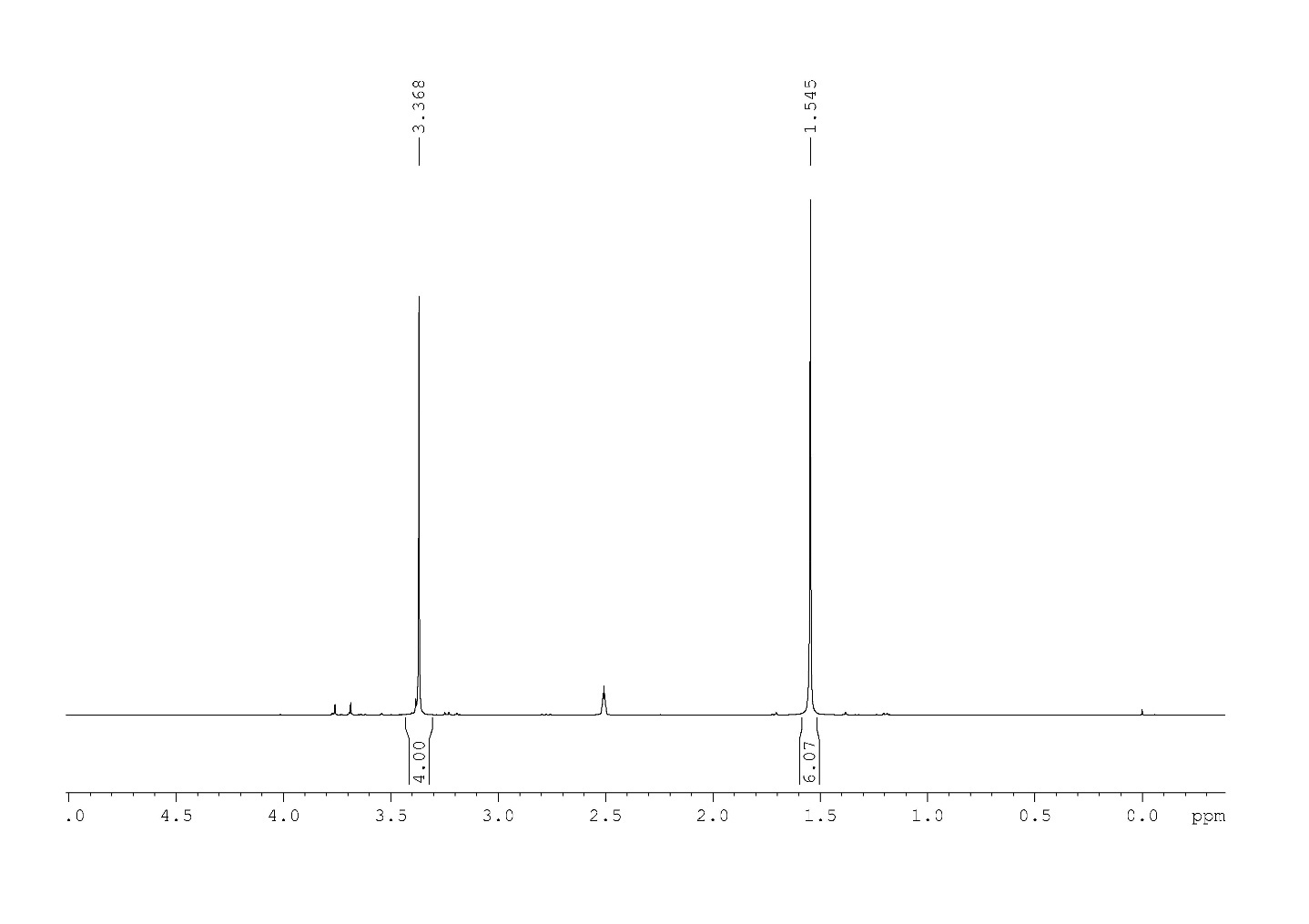


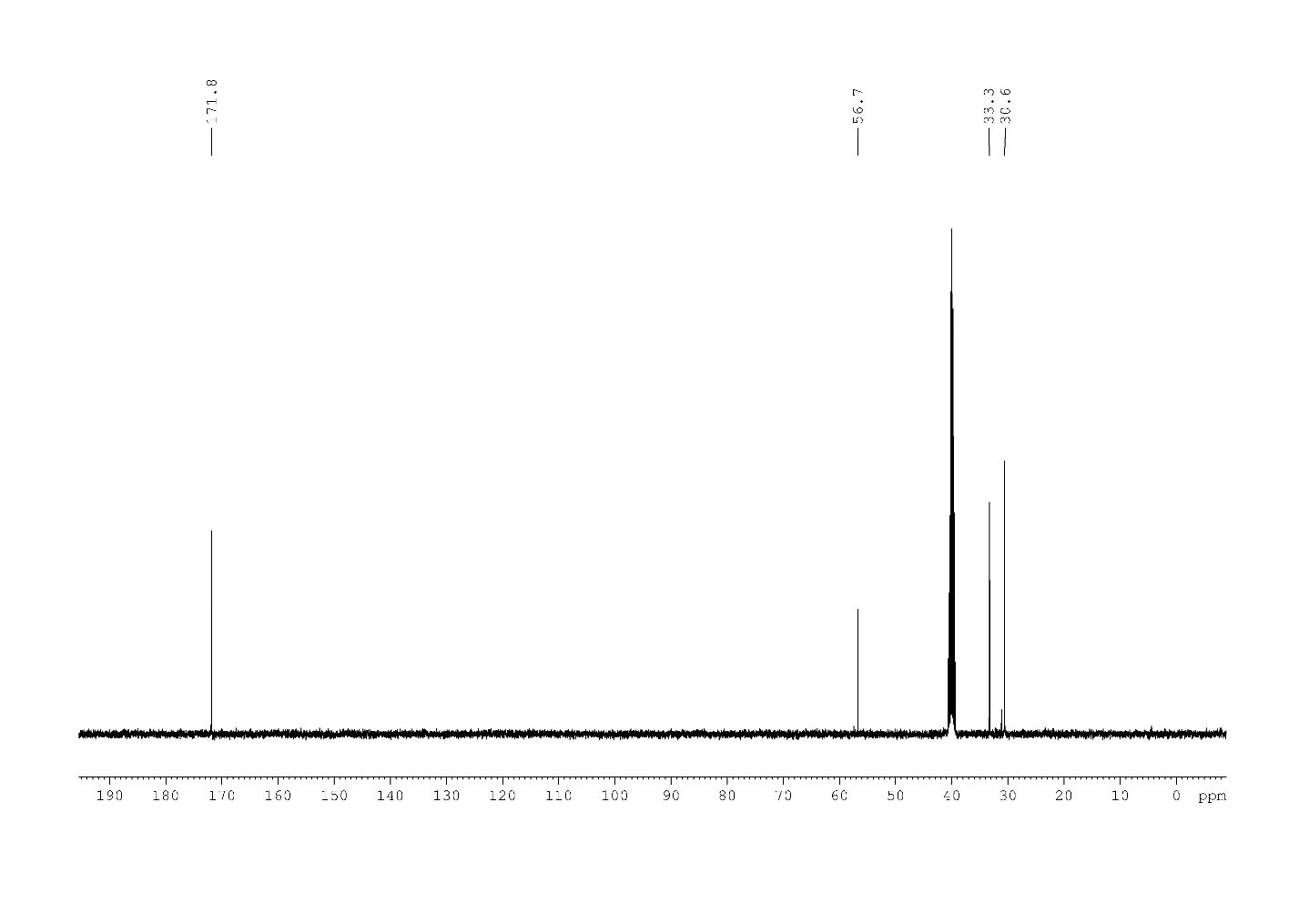


**
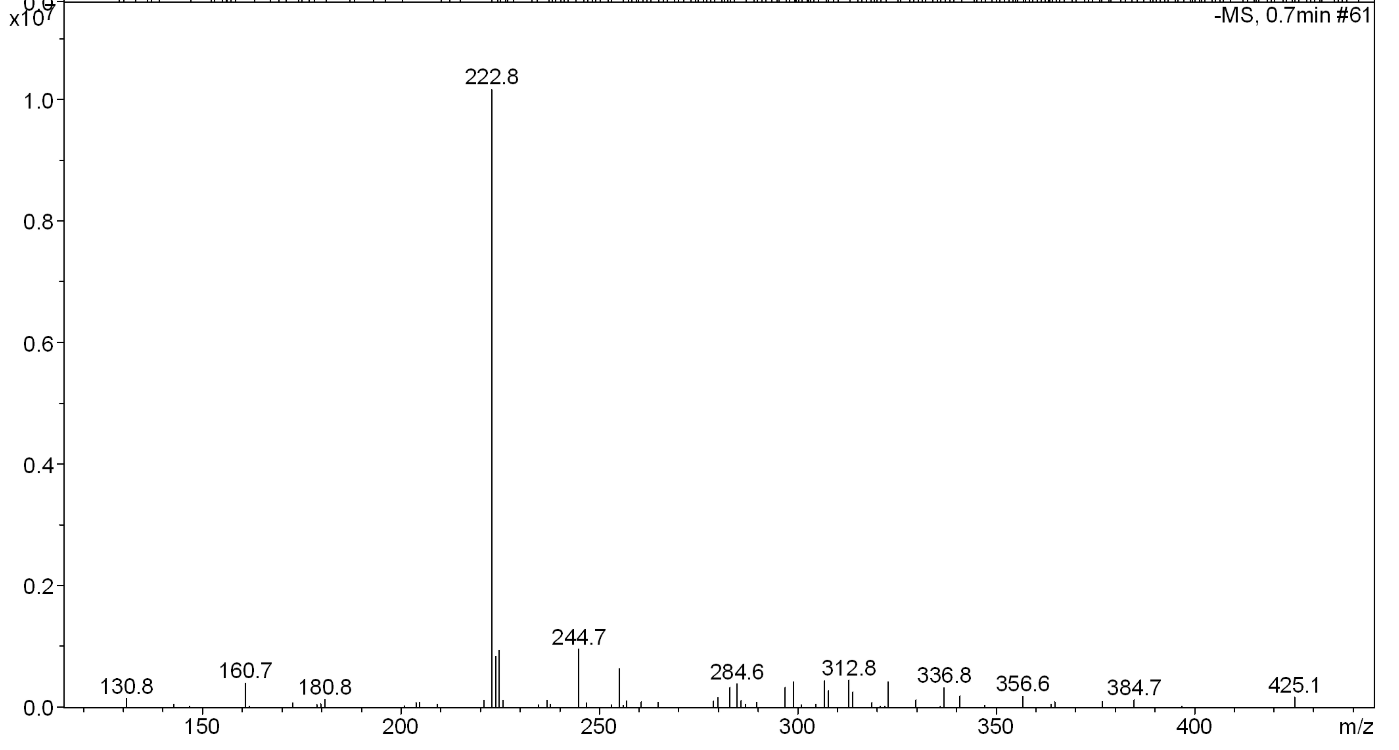
**

[M-H]^-^

**Fig. S7.** ¹H NMR spectrum (DMSO-*d*_6_), ¹³C NMR spectrum (DMSO-*d*_6_), and MS spectrum (ESI) of compound **7a**.


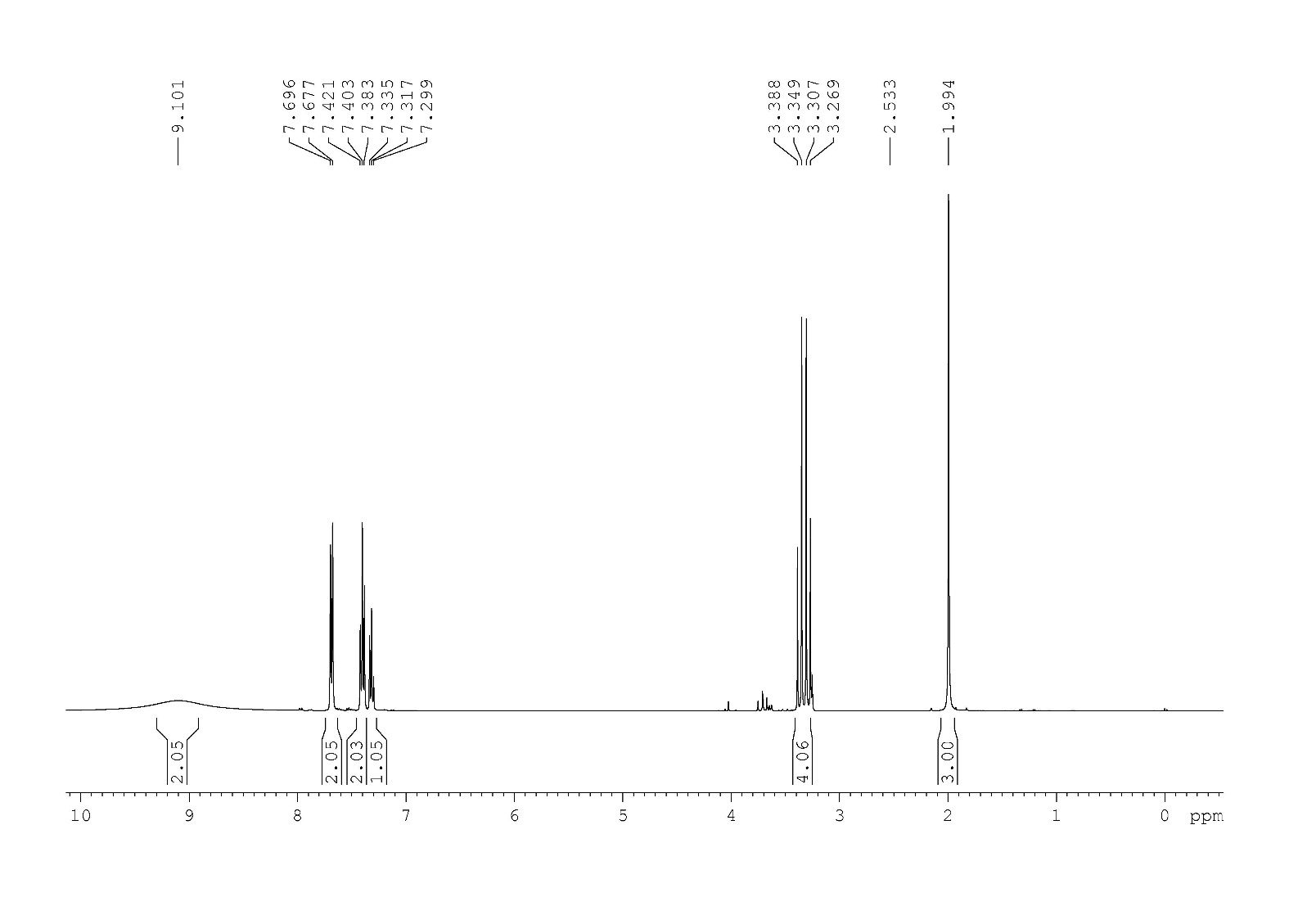


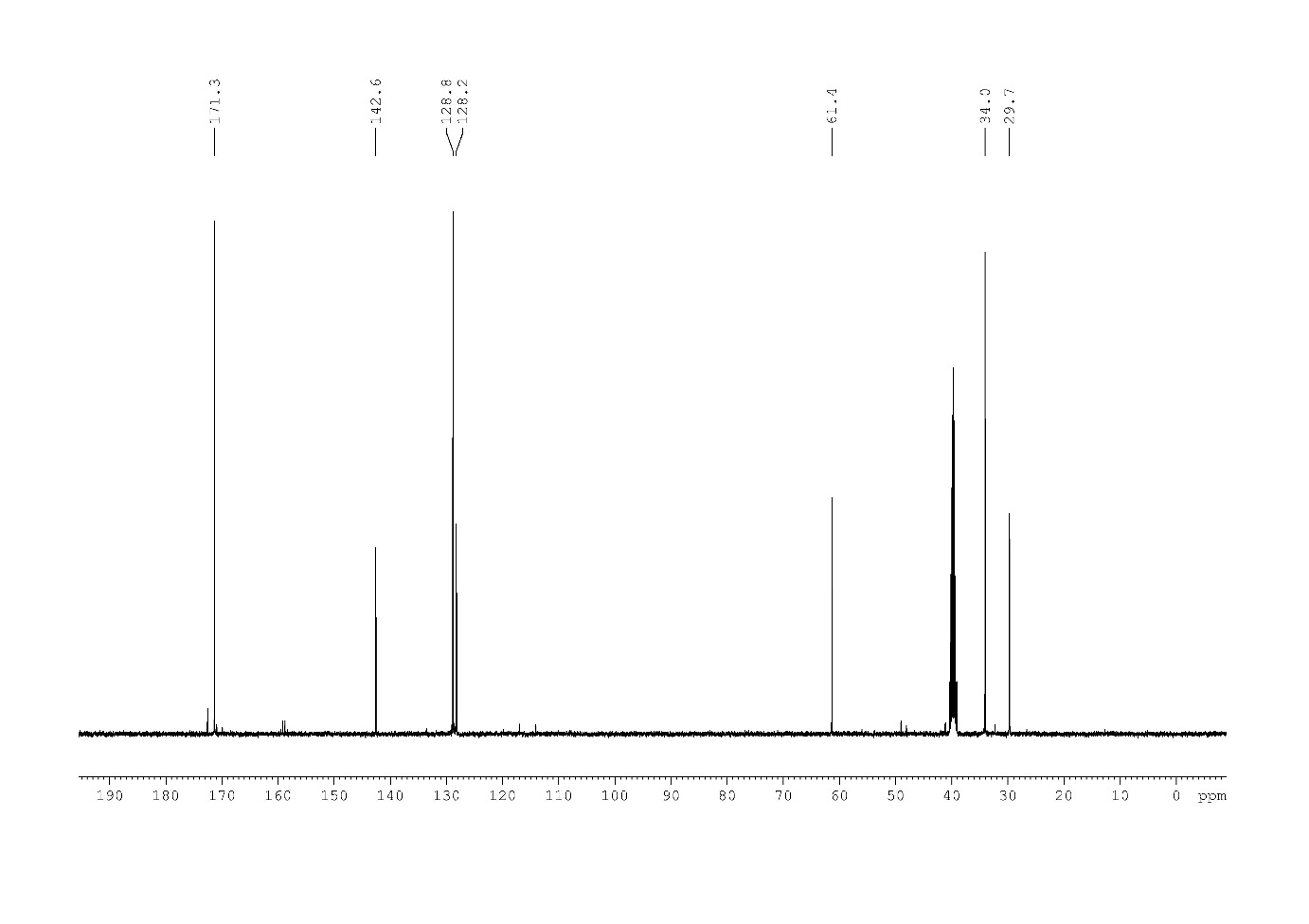


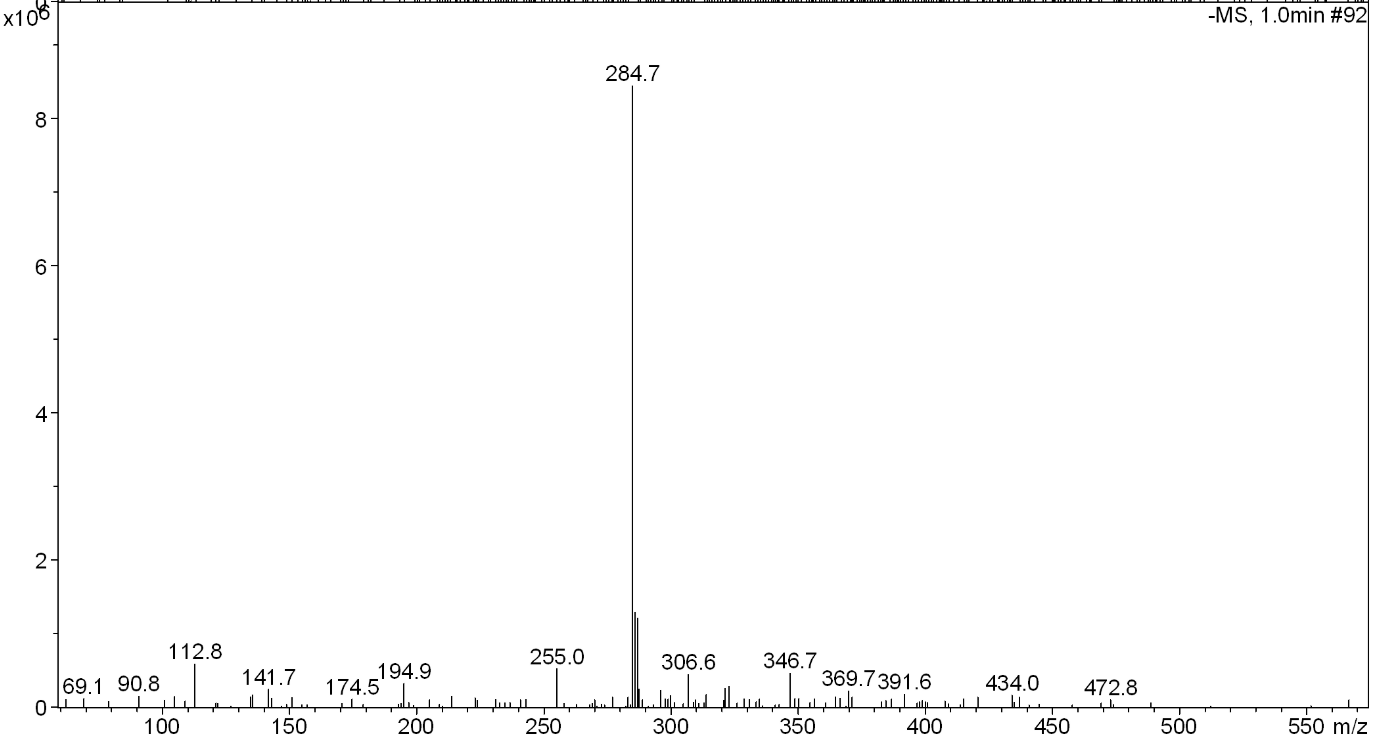


[M-H]^-^

**Fig. S8.** ¹H NMR spectrum (DMSO-*d*_6_), ¹³C NMR spectrum (DMSO-*d*_6_), and MS spectrum (ESI) of compound **7b**.


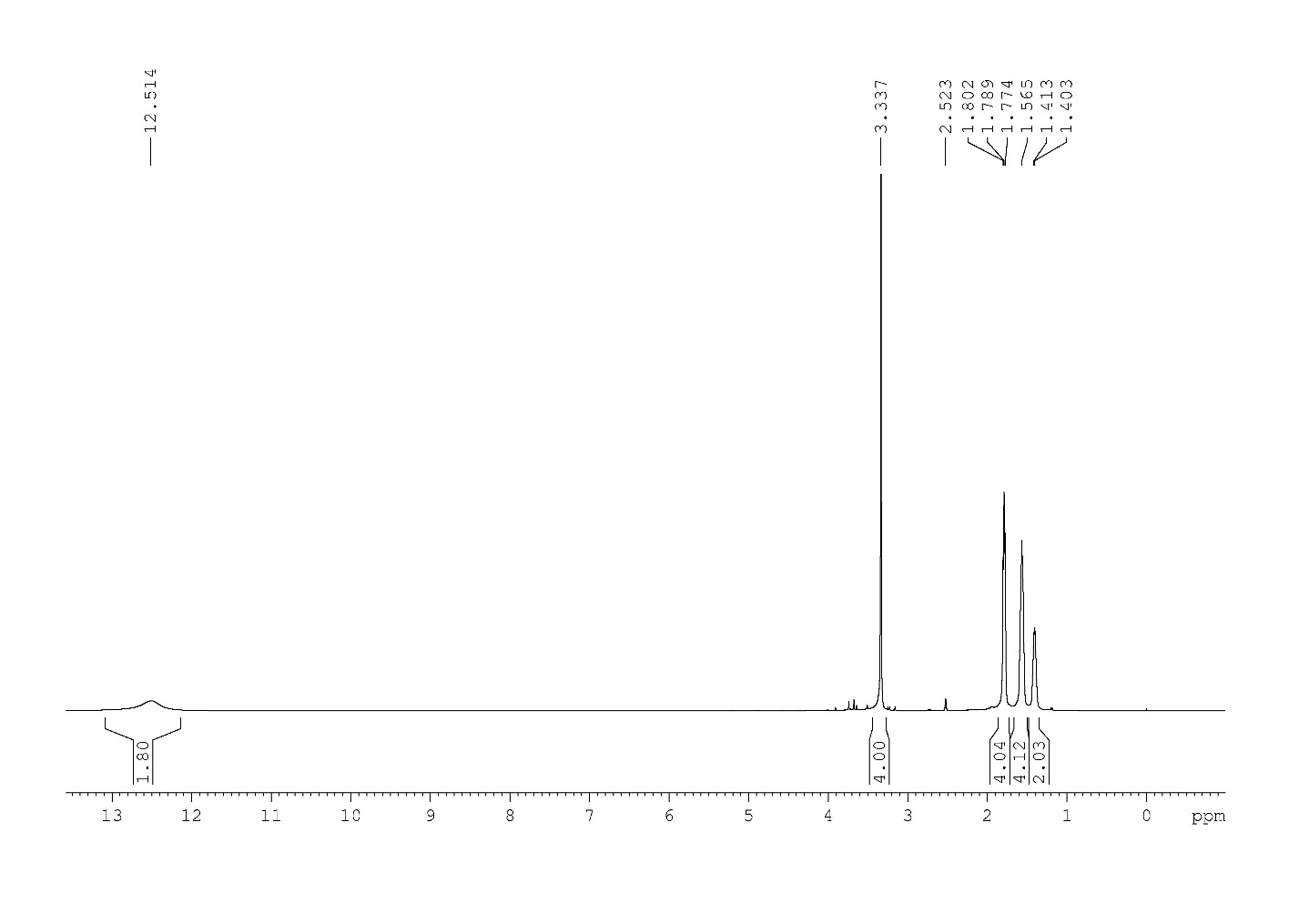


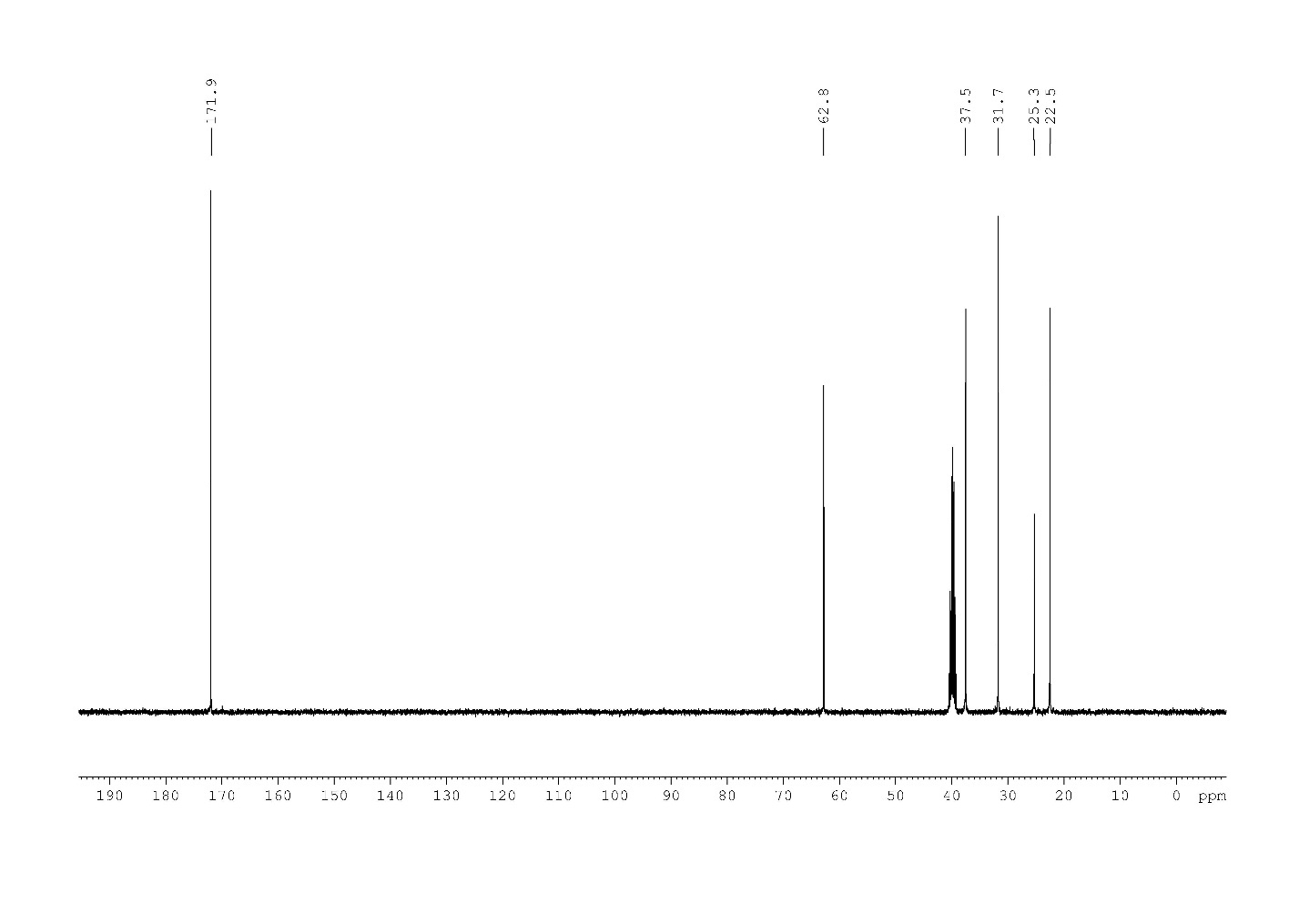


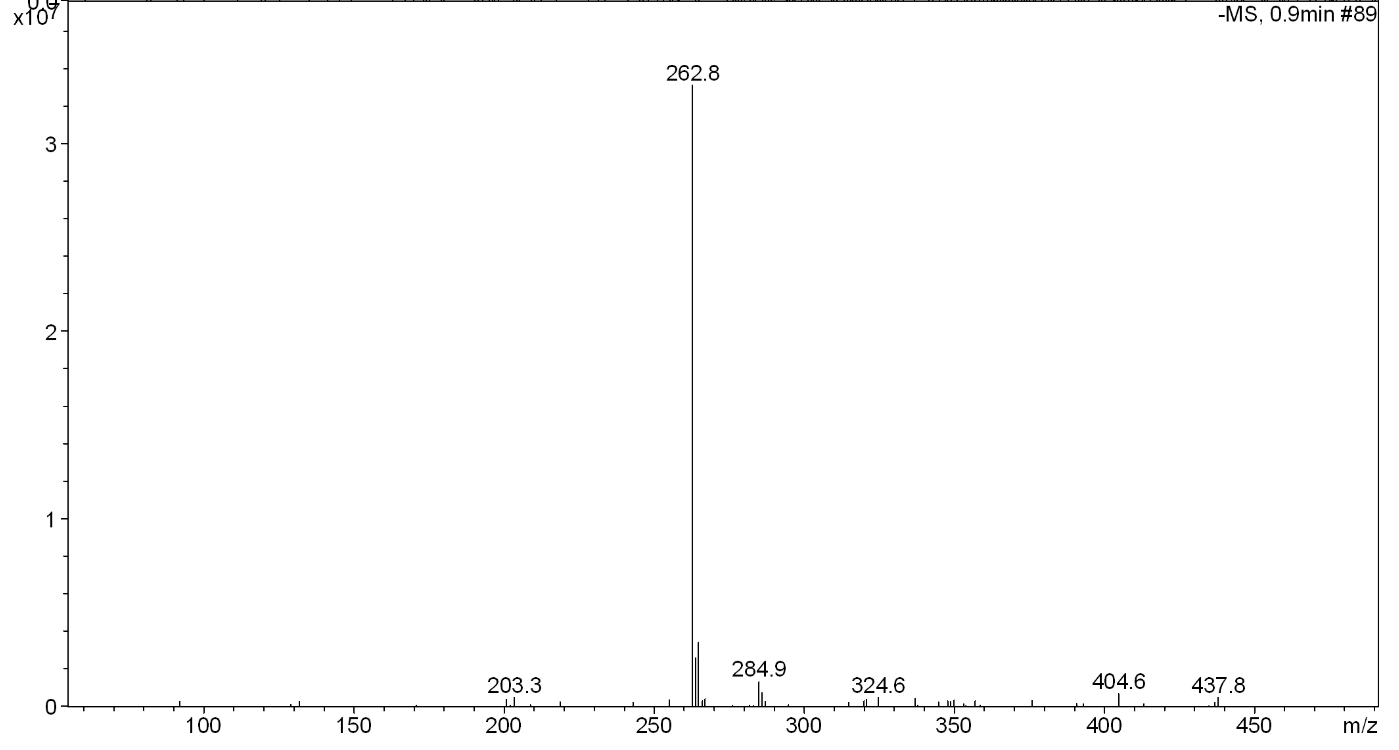


[M-H]^-^

**Fig. S9.** ¹H NMR spectrum (DMSO-*d*_6_), ¹³C NMR spectrum (DMSO-*d*_6_), and MS spectrum (ESI) of compound **7c**.


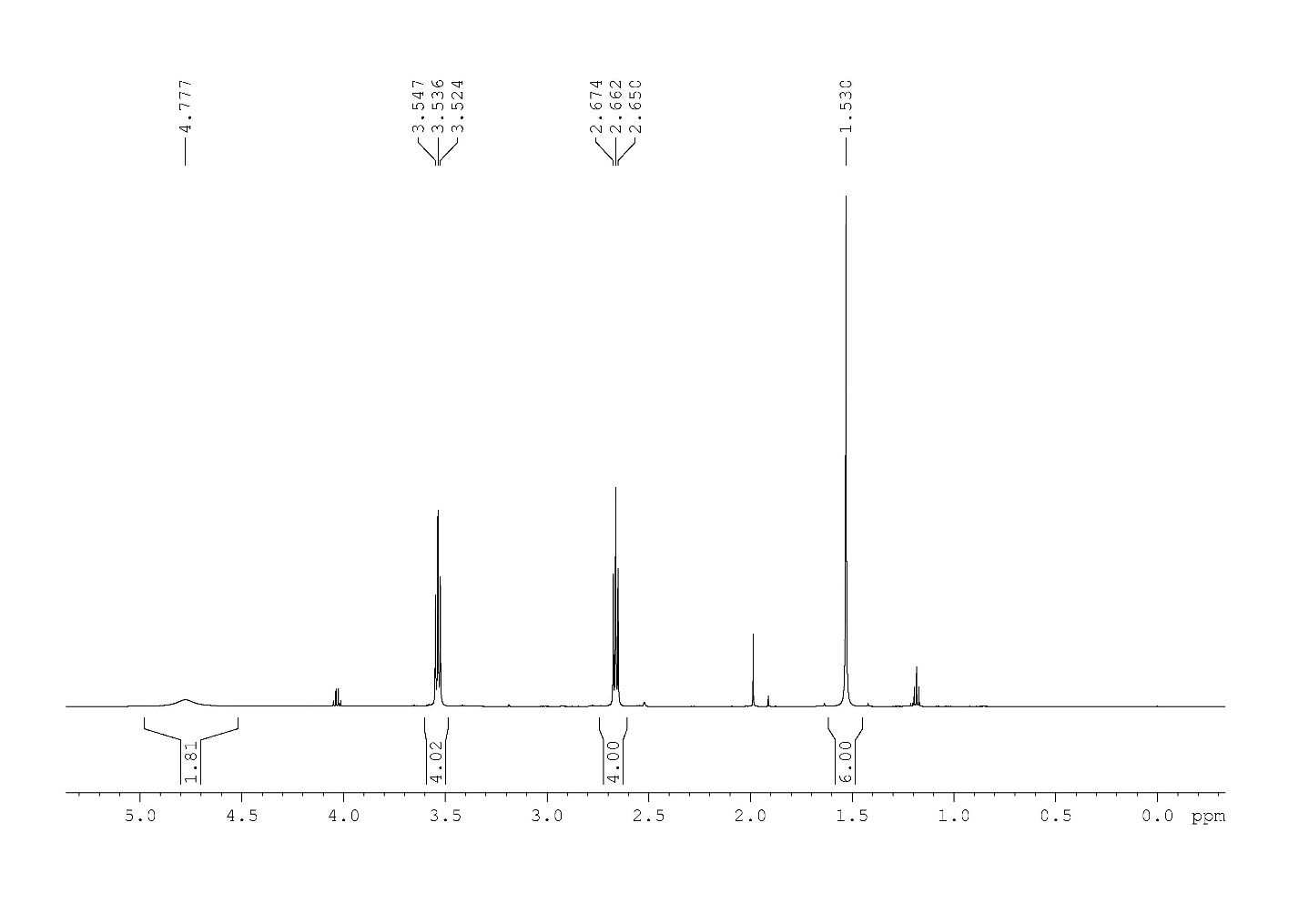


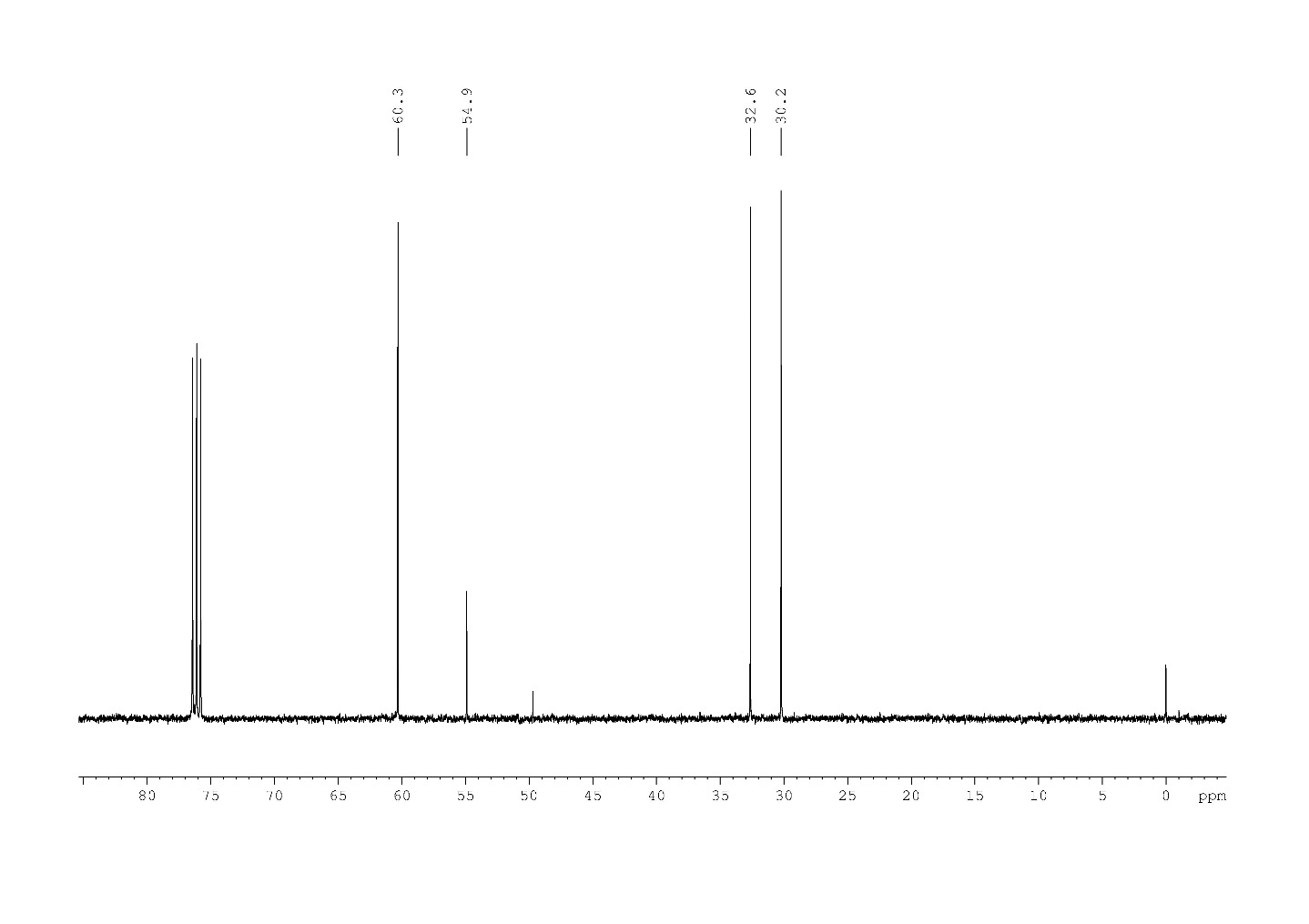


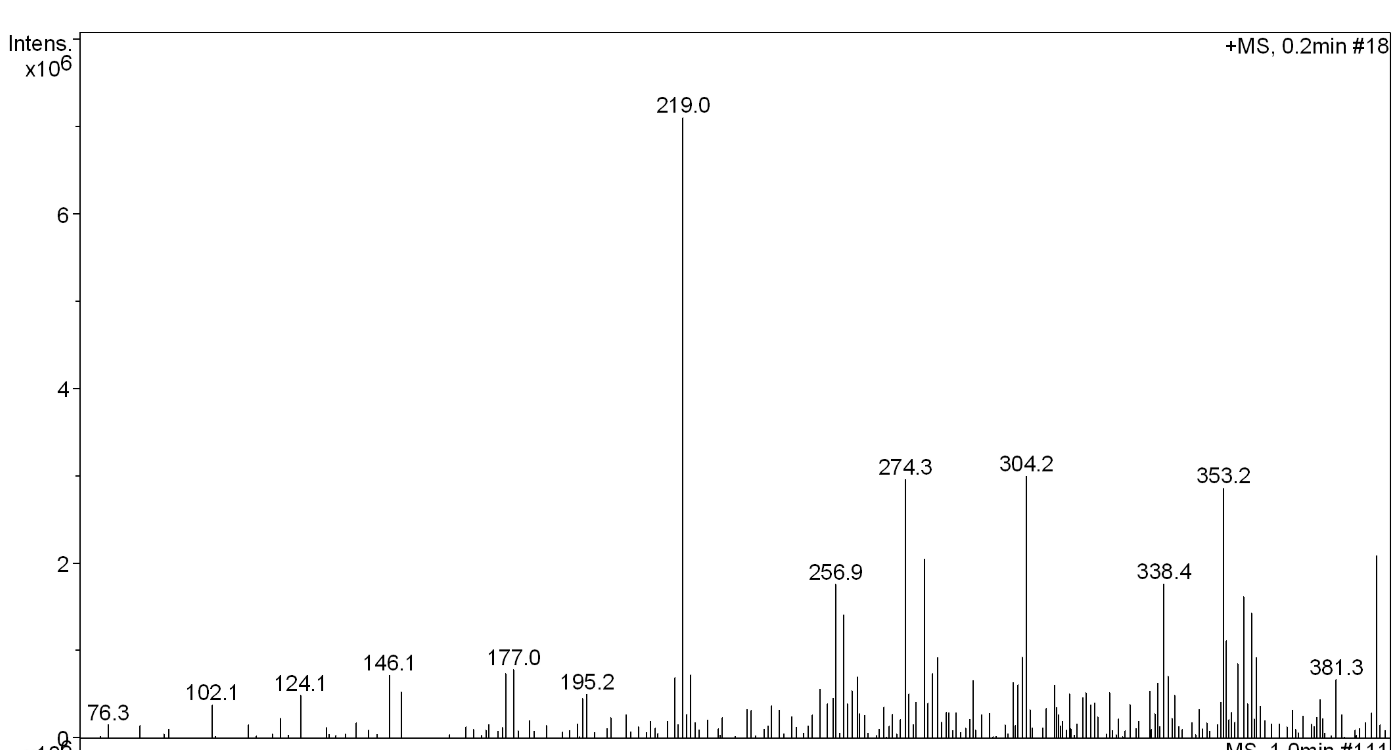


[M+Na]^+^

**Fig. S10.** ¹H NMR spectrum (CDCl_3_), ¹³C NMR spectrum (CDCl_3_), and MS spectrum (ESI) of compound **8a**.


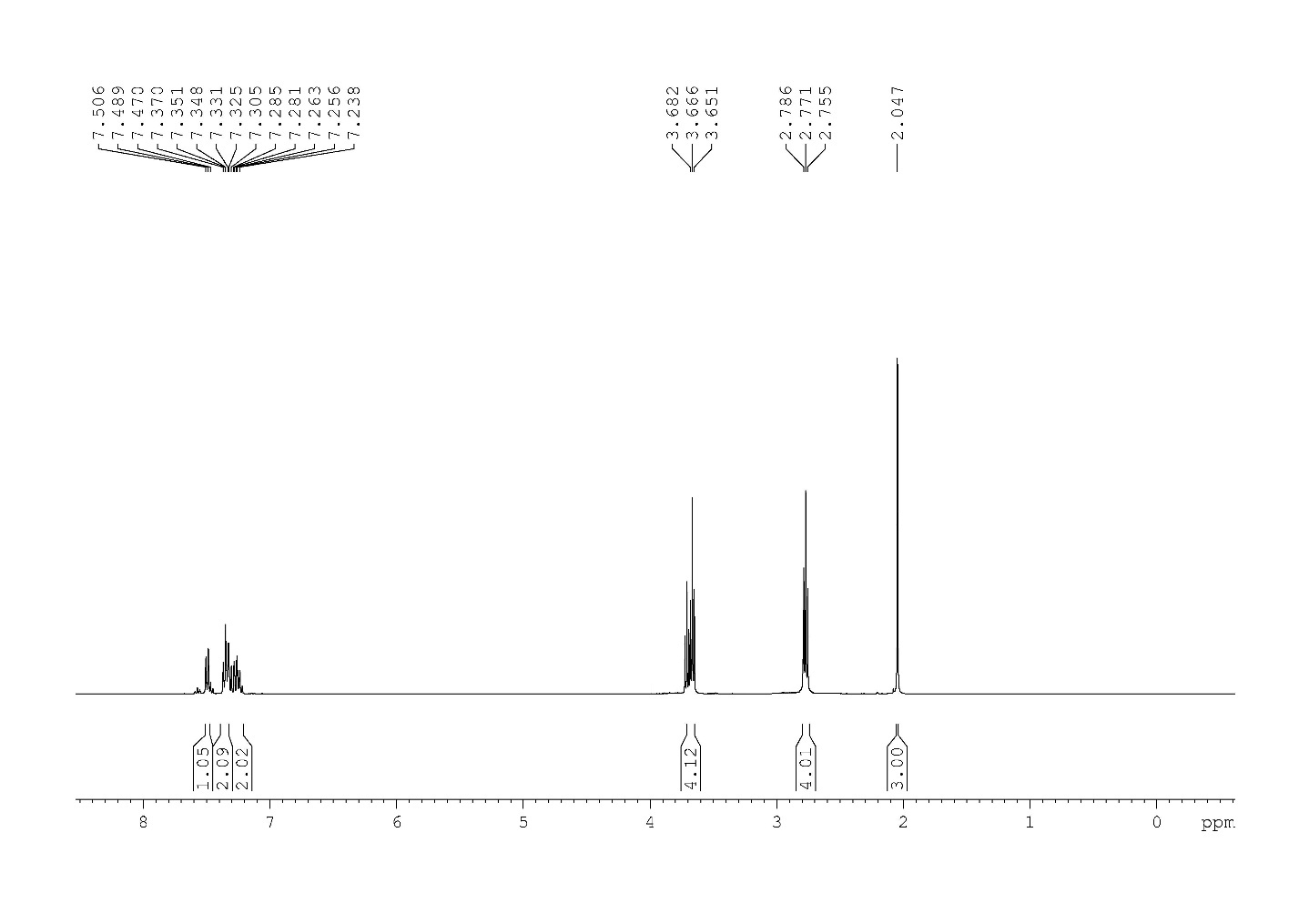


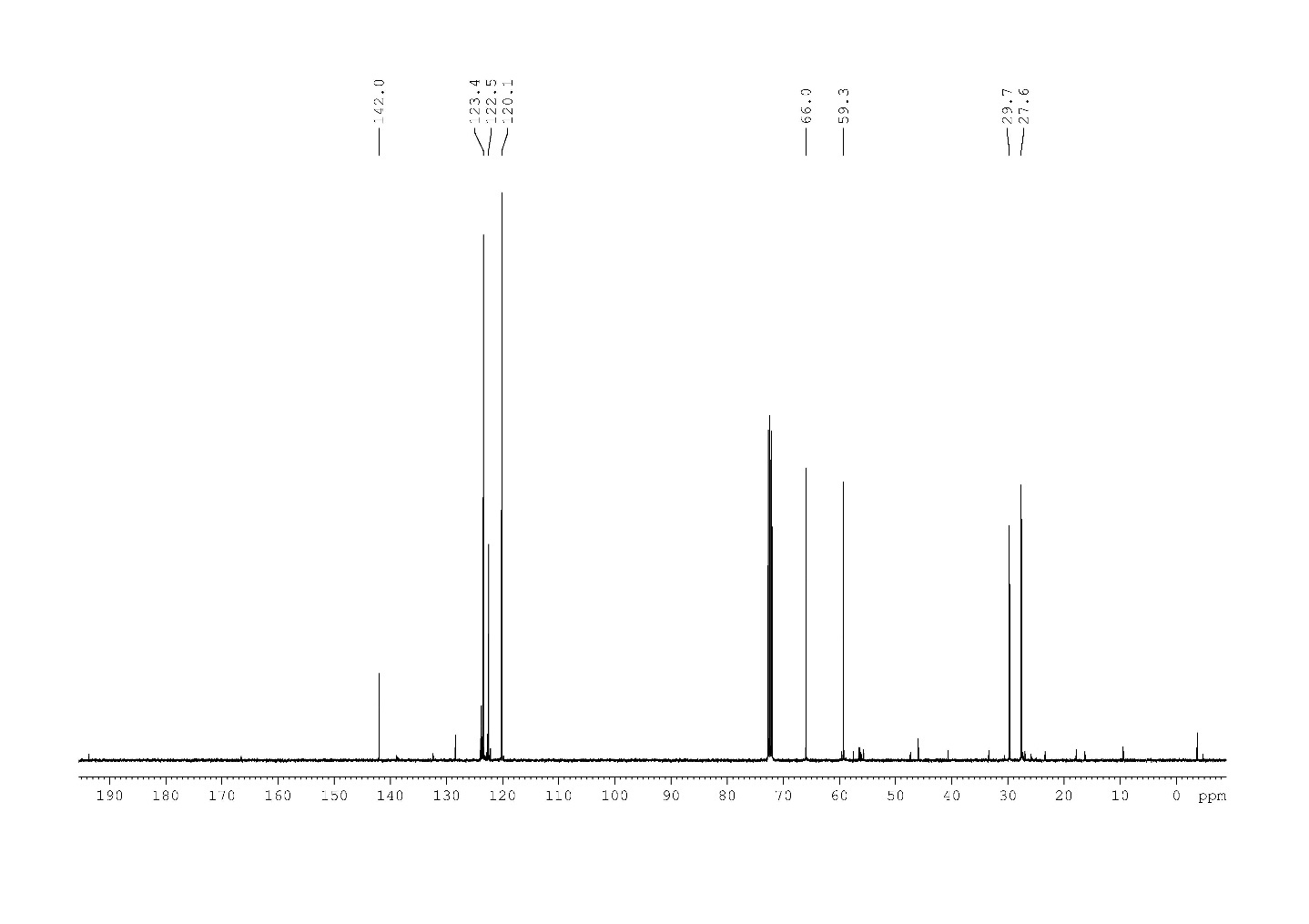


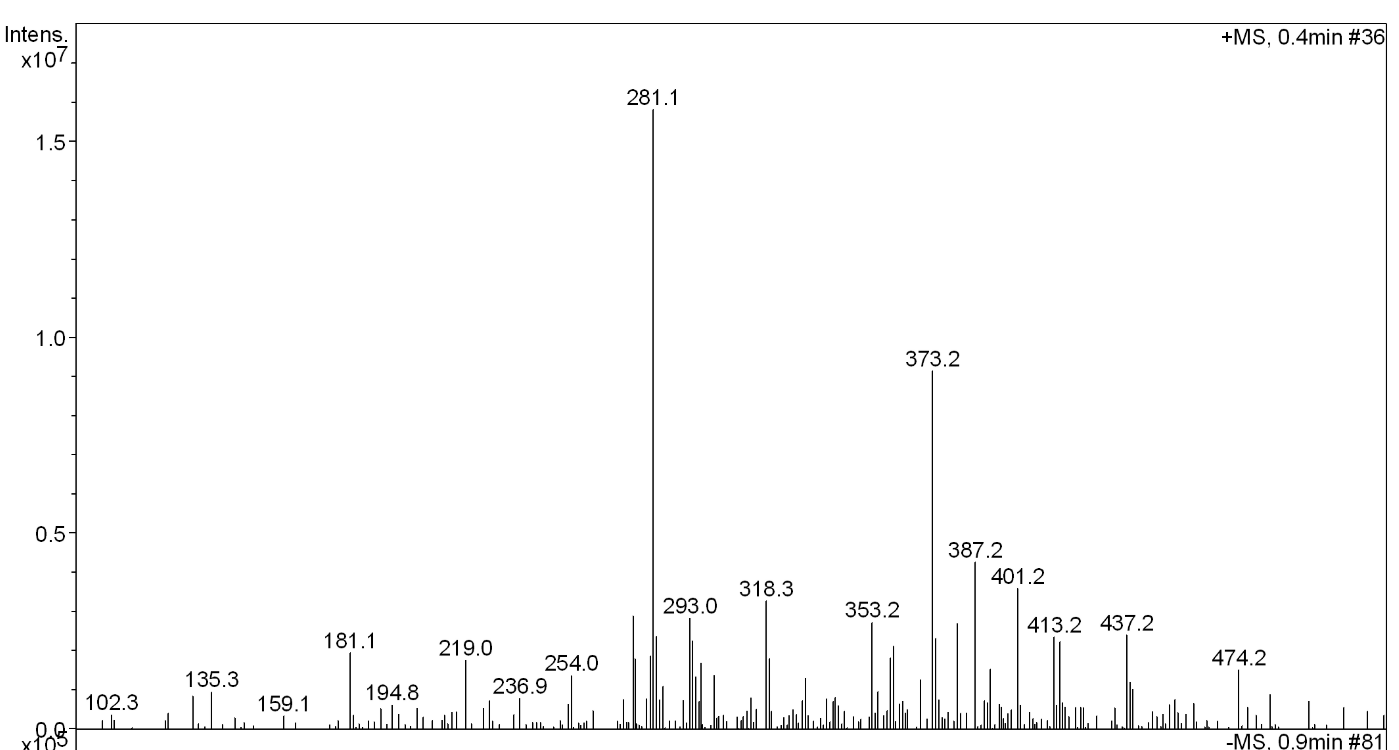


[M+Na]^+^

**Fig. S11.** ¹H NMR spectrum (CDCl_3_), ¹³C NMR spectrum (CDCl_3_), and MS spectrum (ESI) of compound **8b**.


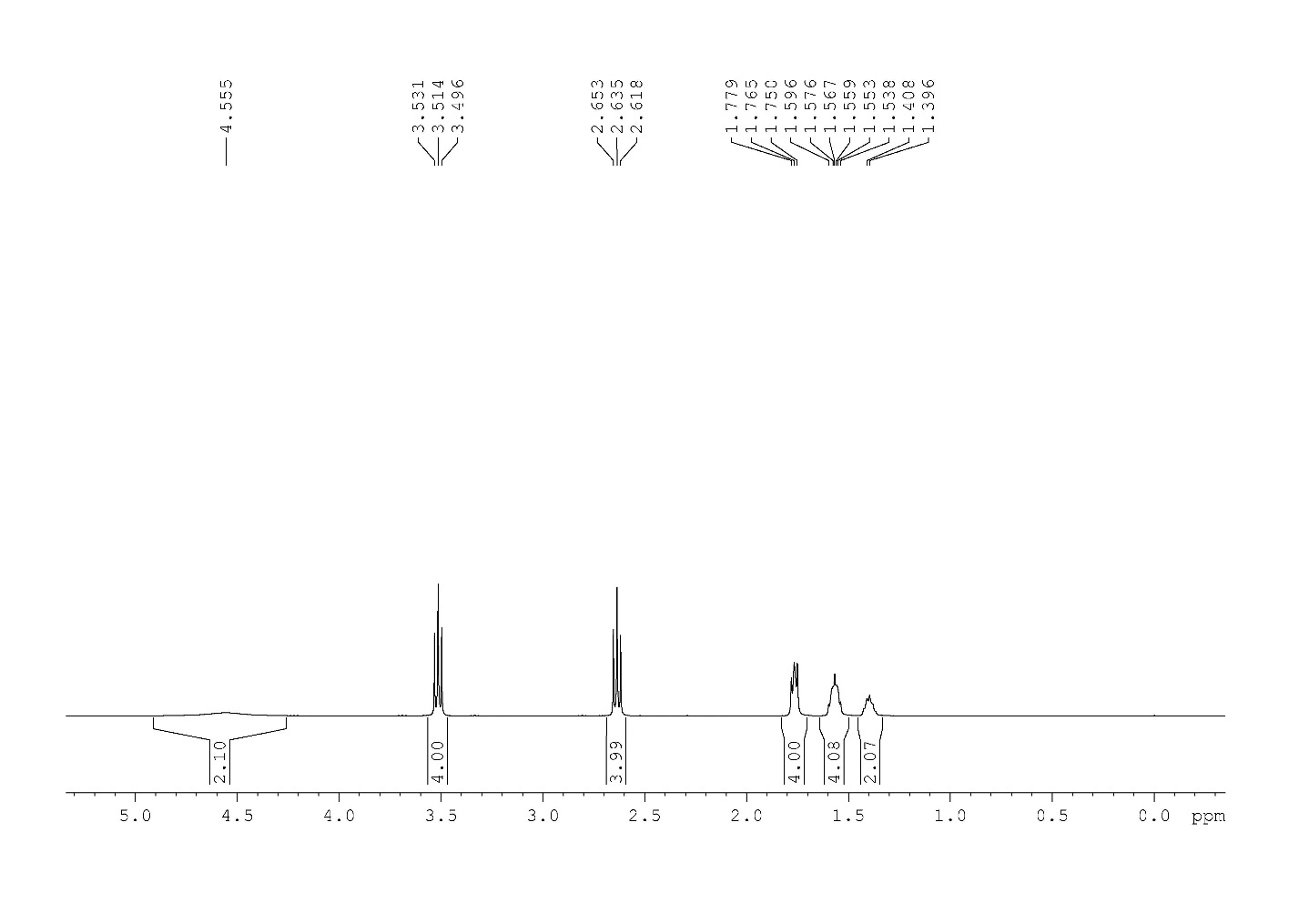


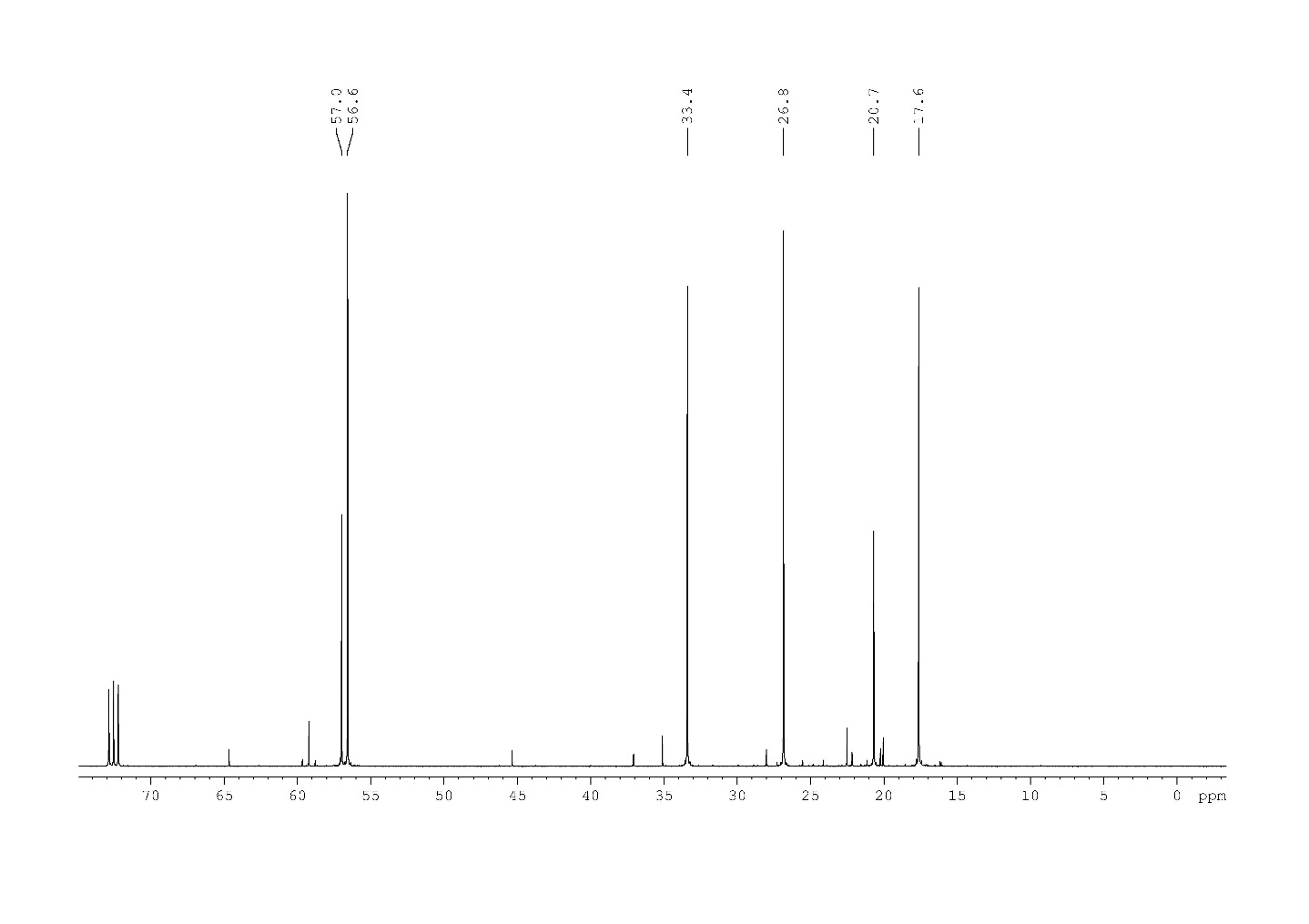


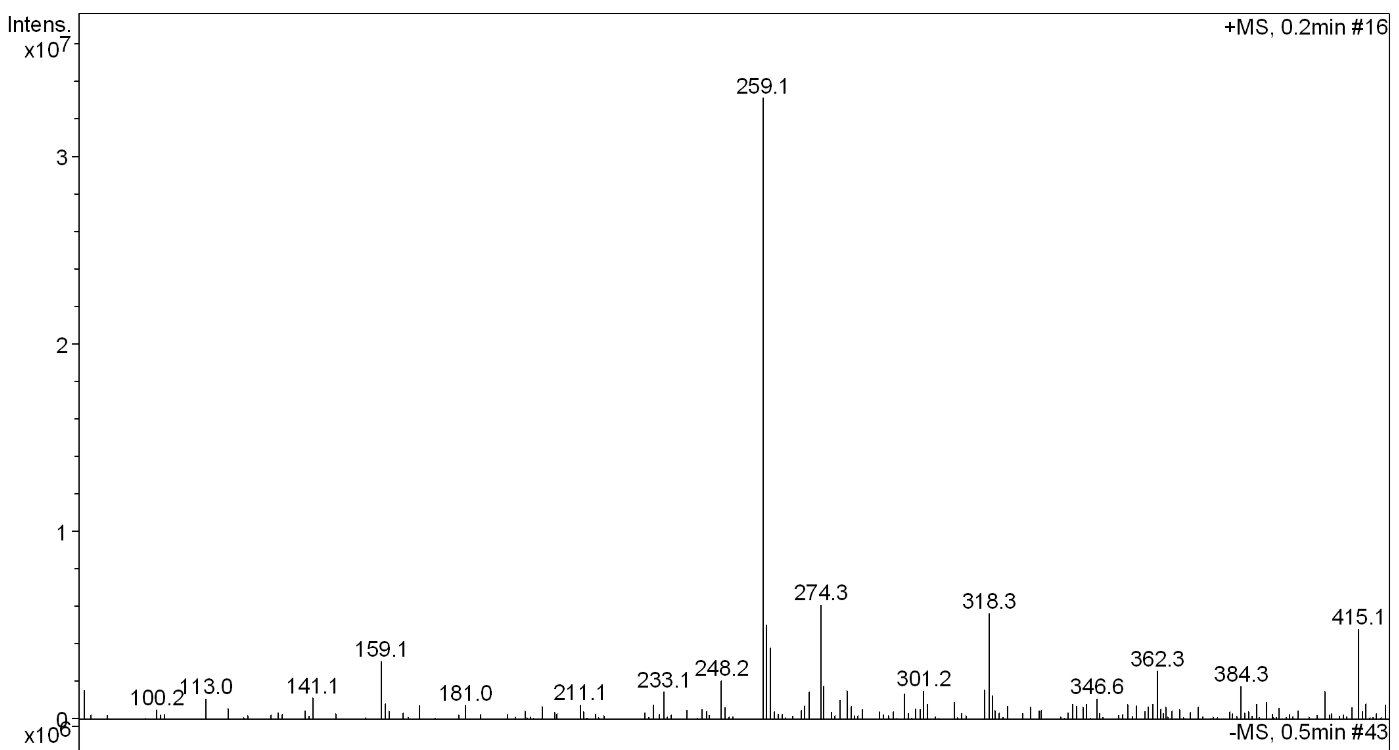


[M+Na]^+^

**Fig. S12.** ¹H NMR spectrum (CDCl_3_), ¹³C NMR spectrum (CDCl_3_), and MS spectrum (ESI) of compound **8c**.


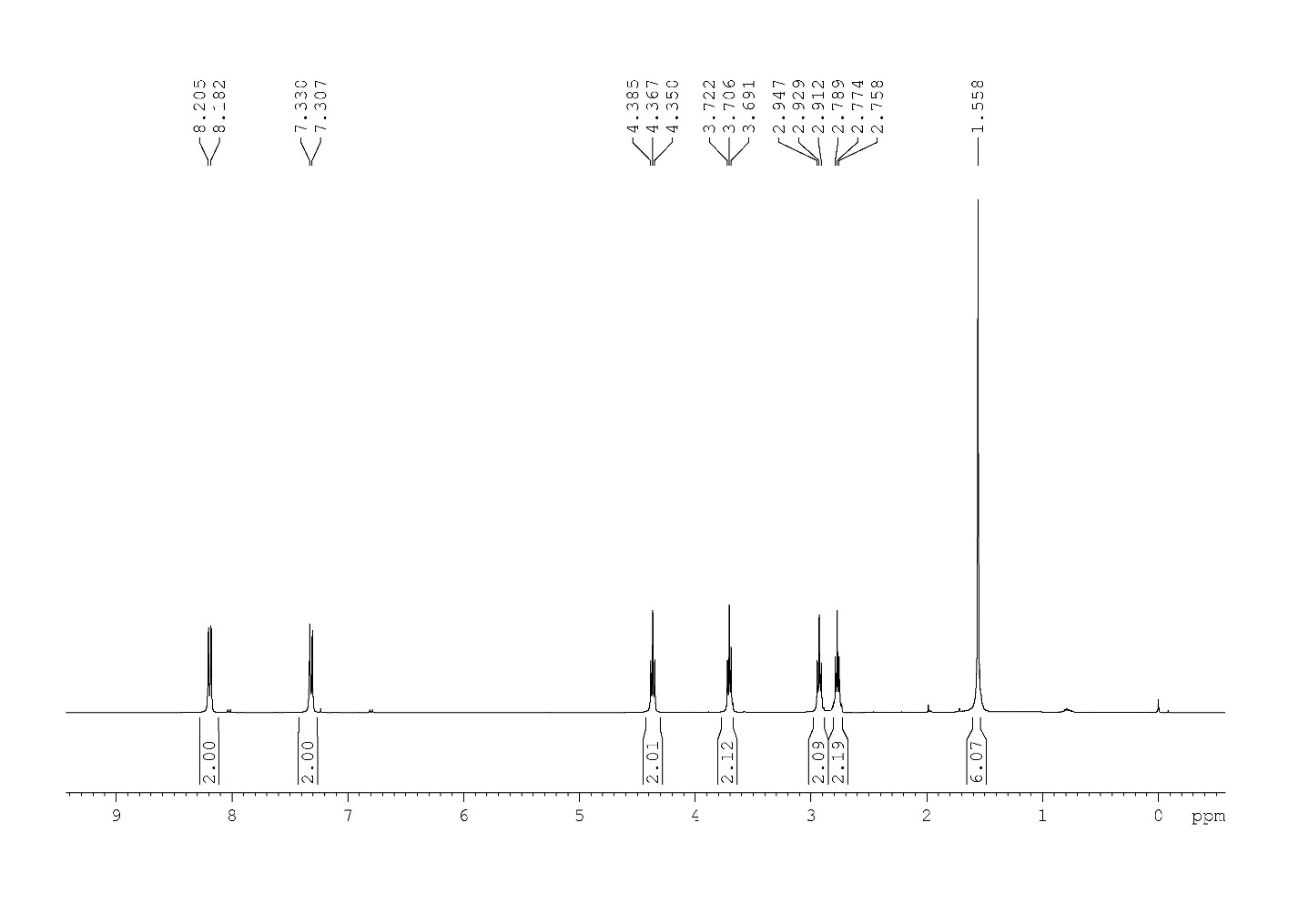


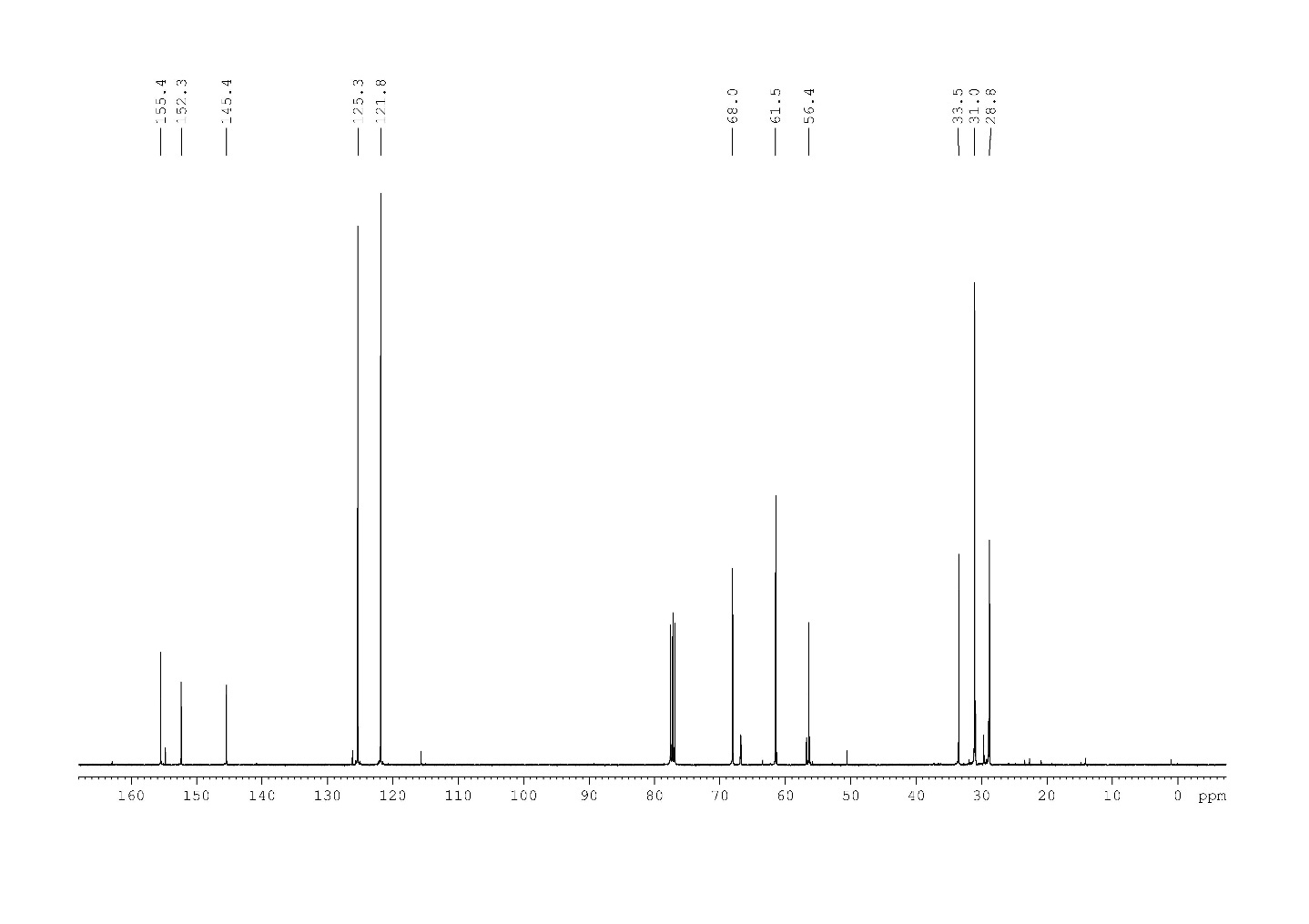


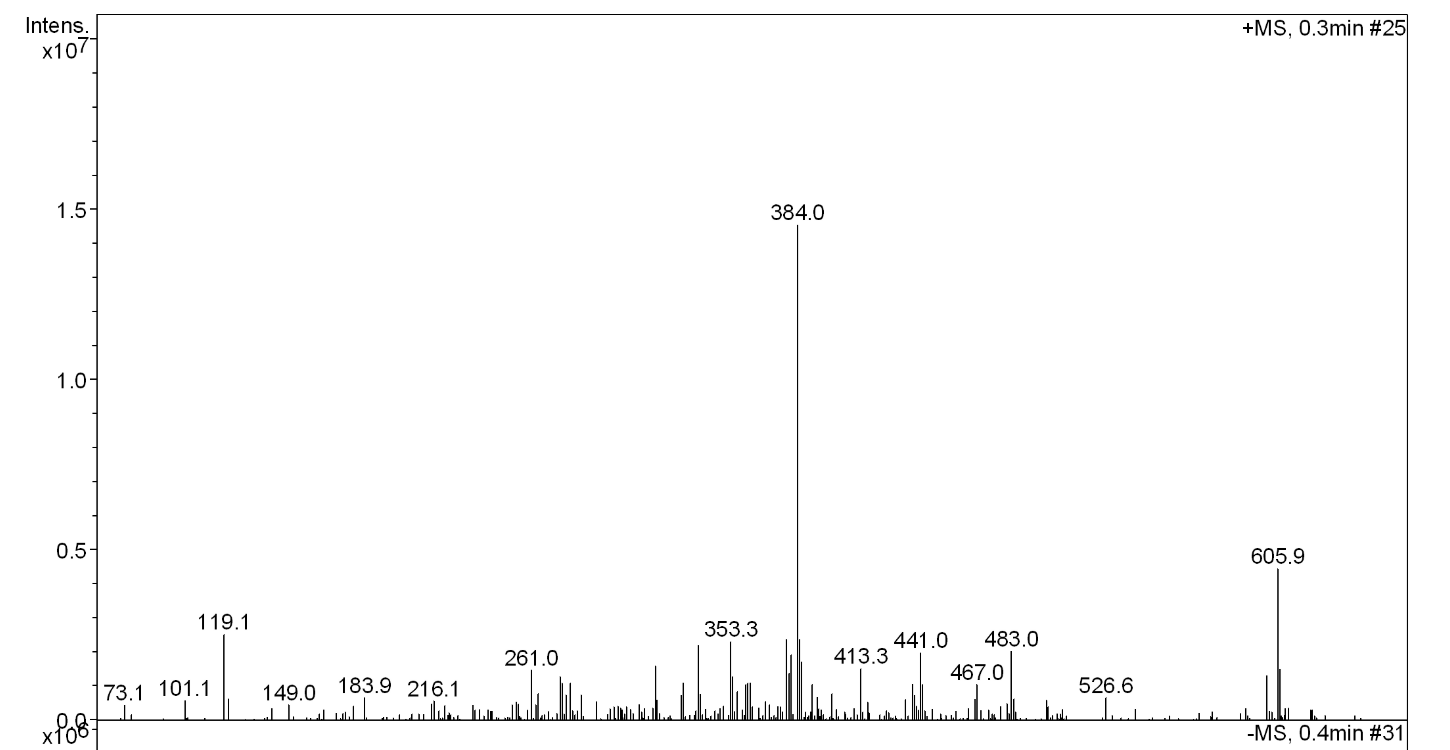


[M+Na]^+^

**Fig. S13.** ¹H NMR spectrum (CDCl_3_), ¹³C NMR spectrum (CDCl_3_), and MS spectrum (ESI) of compound **9a**.


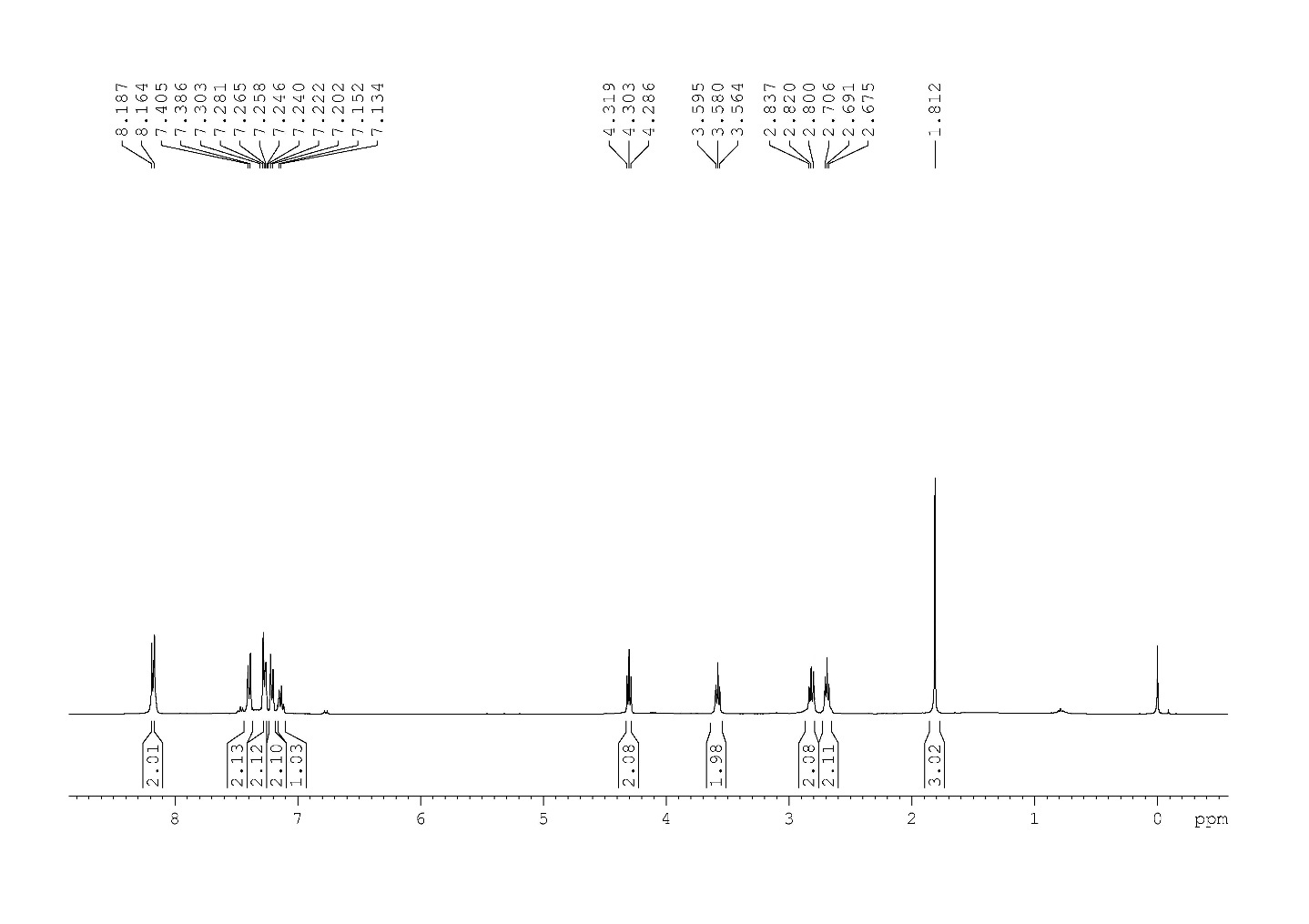


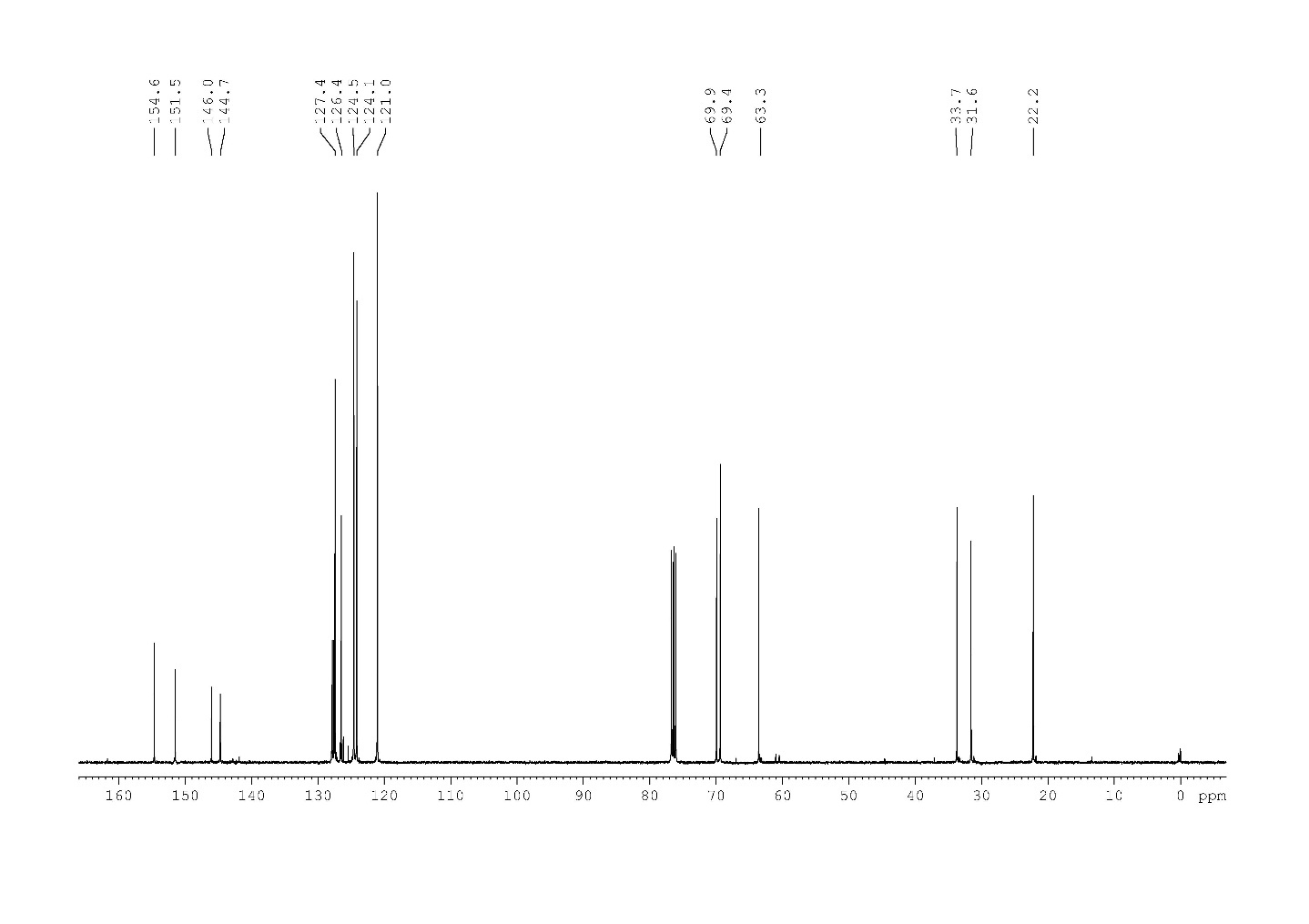


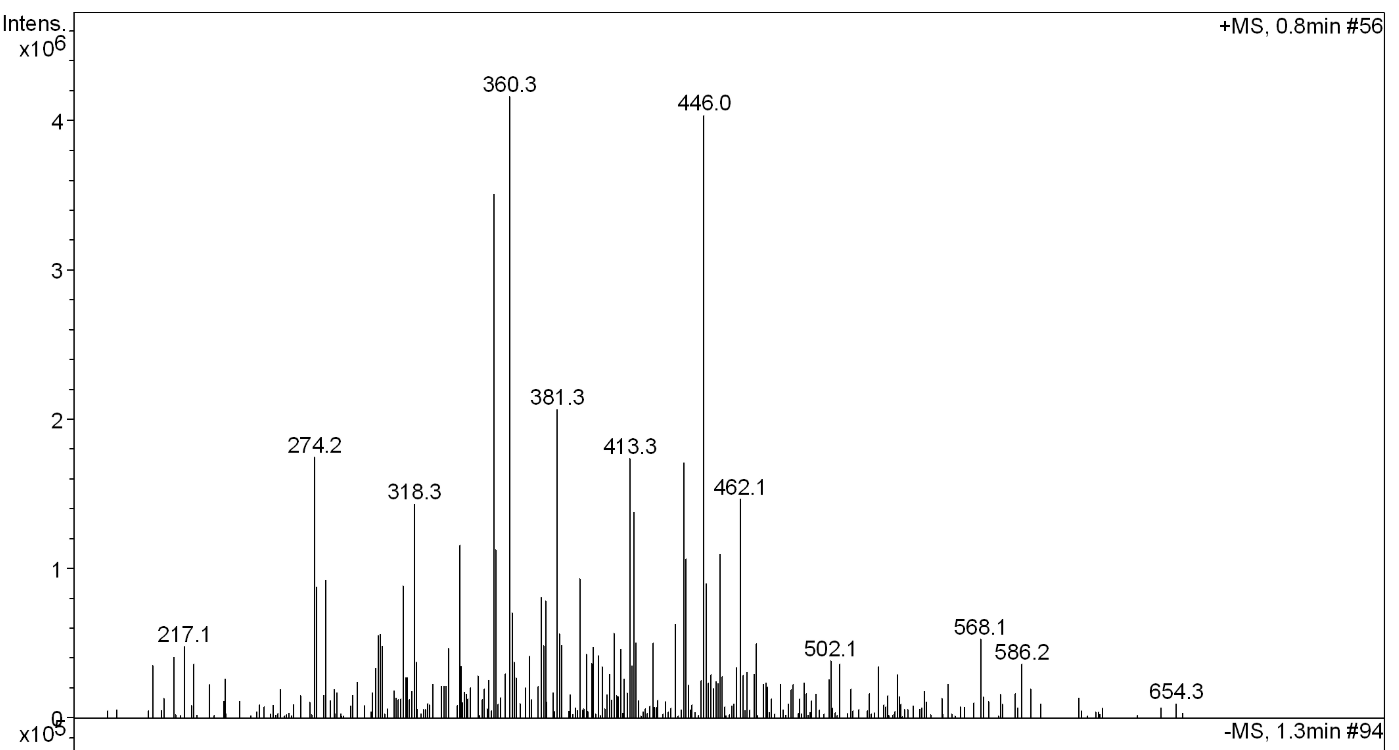


[M+Na]^+^

**Fig. S14.** ¹H NMR spectrum (CDCl_3_), ¹³C NMR spectrum (CDCl_3_), and MS spectrum (ESI) of compound **9b**.


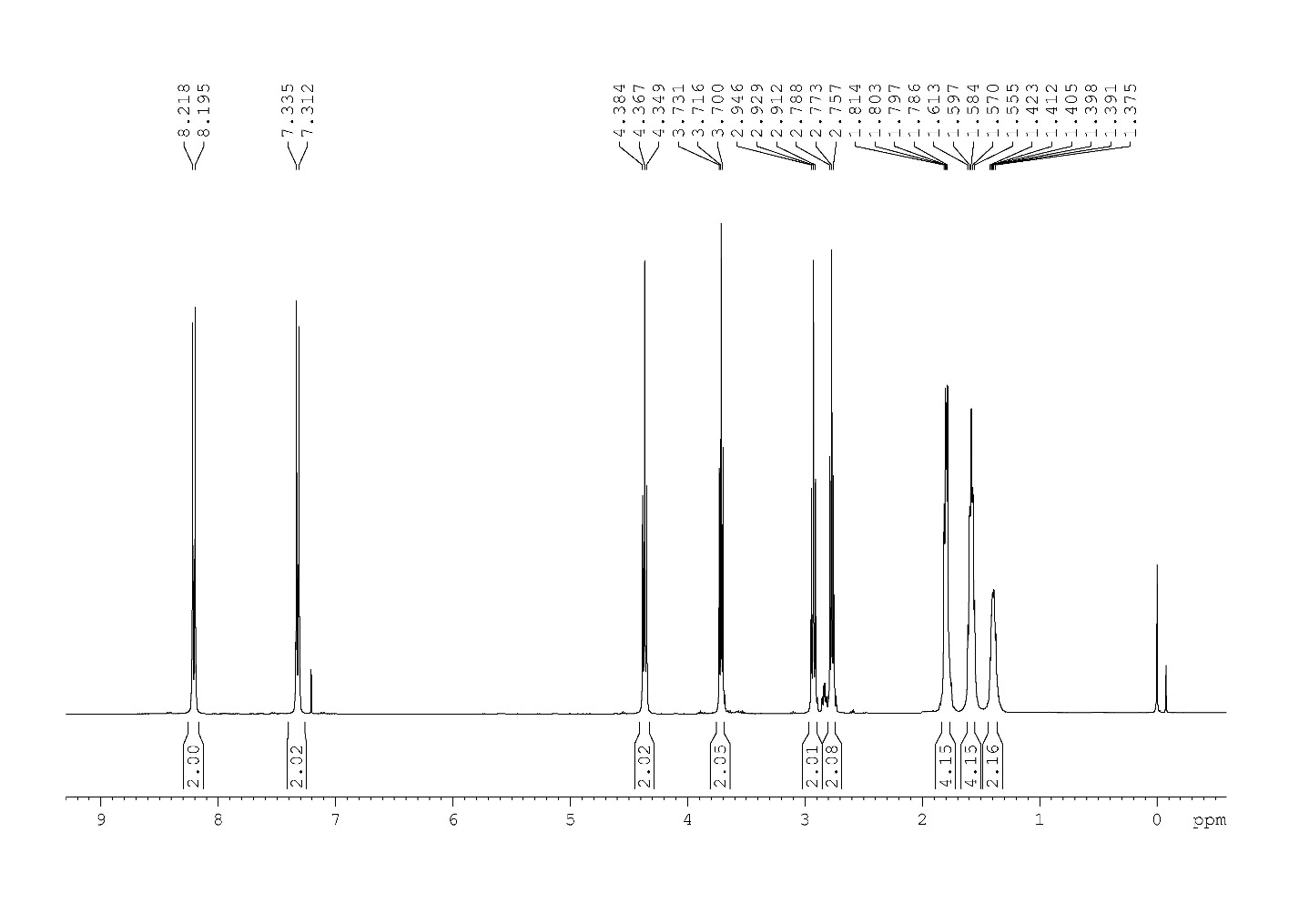


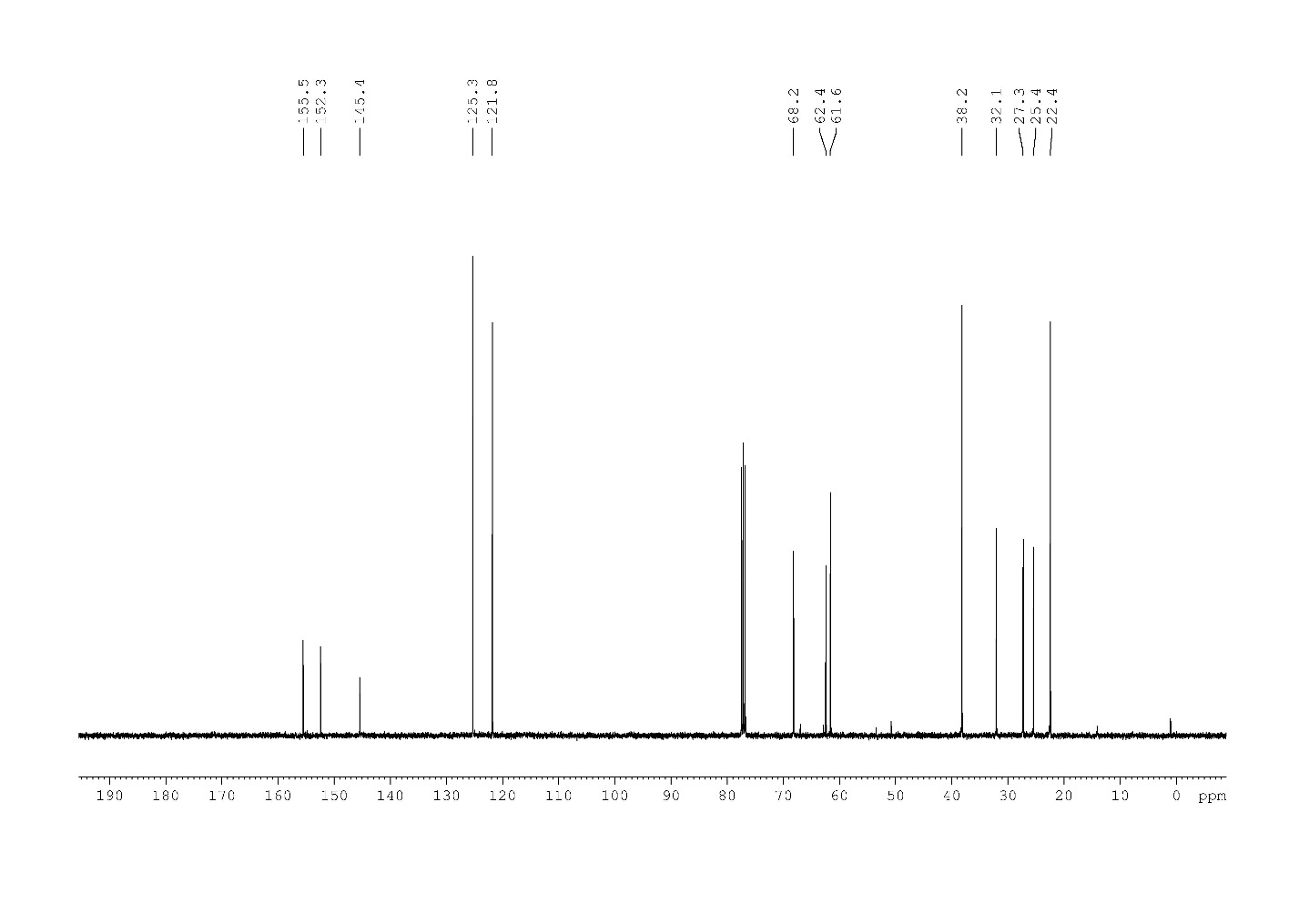


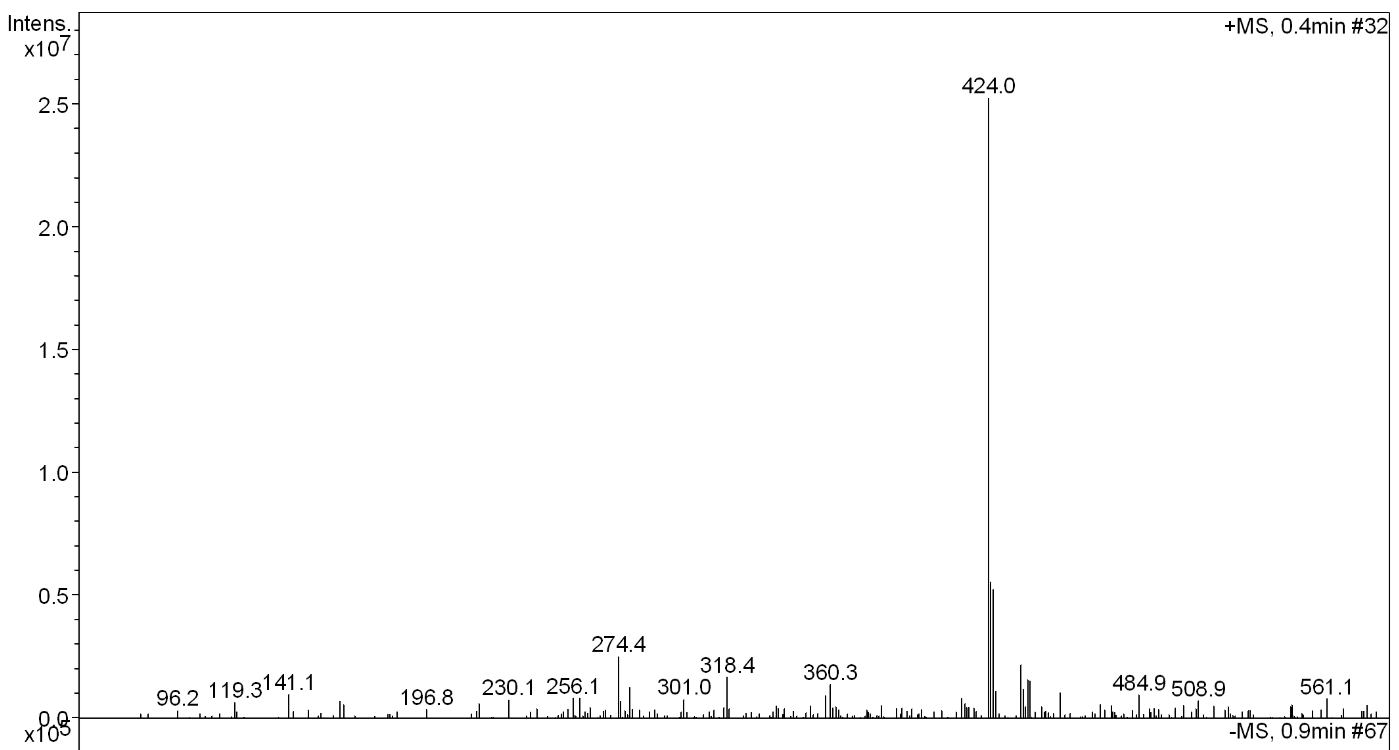


[M+Na]^+^

**Fig. S15.** ¹H NMR spectrum (CDCl_3_), ¹³C NMR spectrum (CDCl_3_), and MS spectrum (ESI) of compound **9c**.


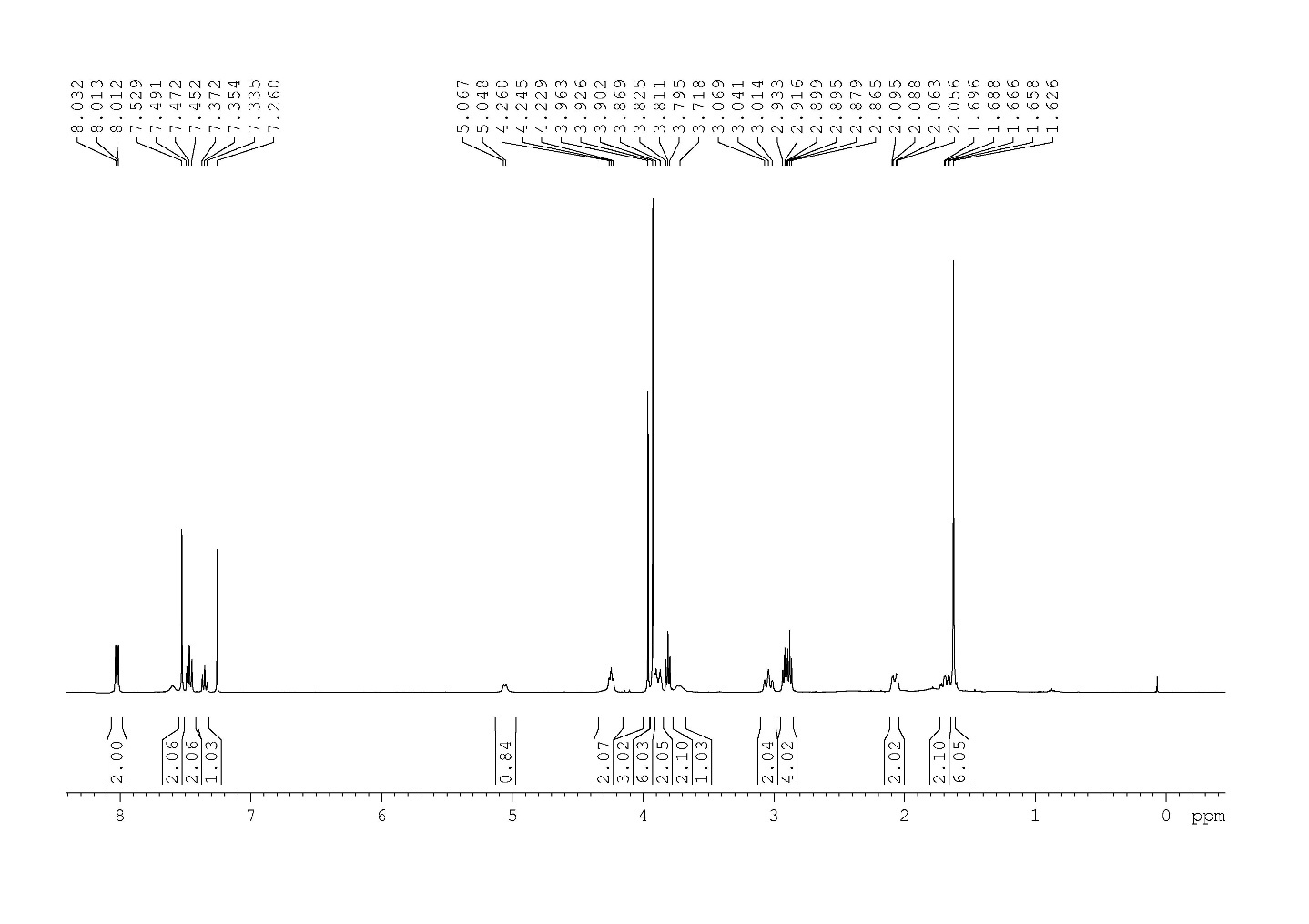


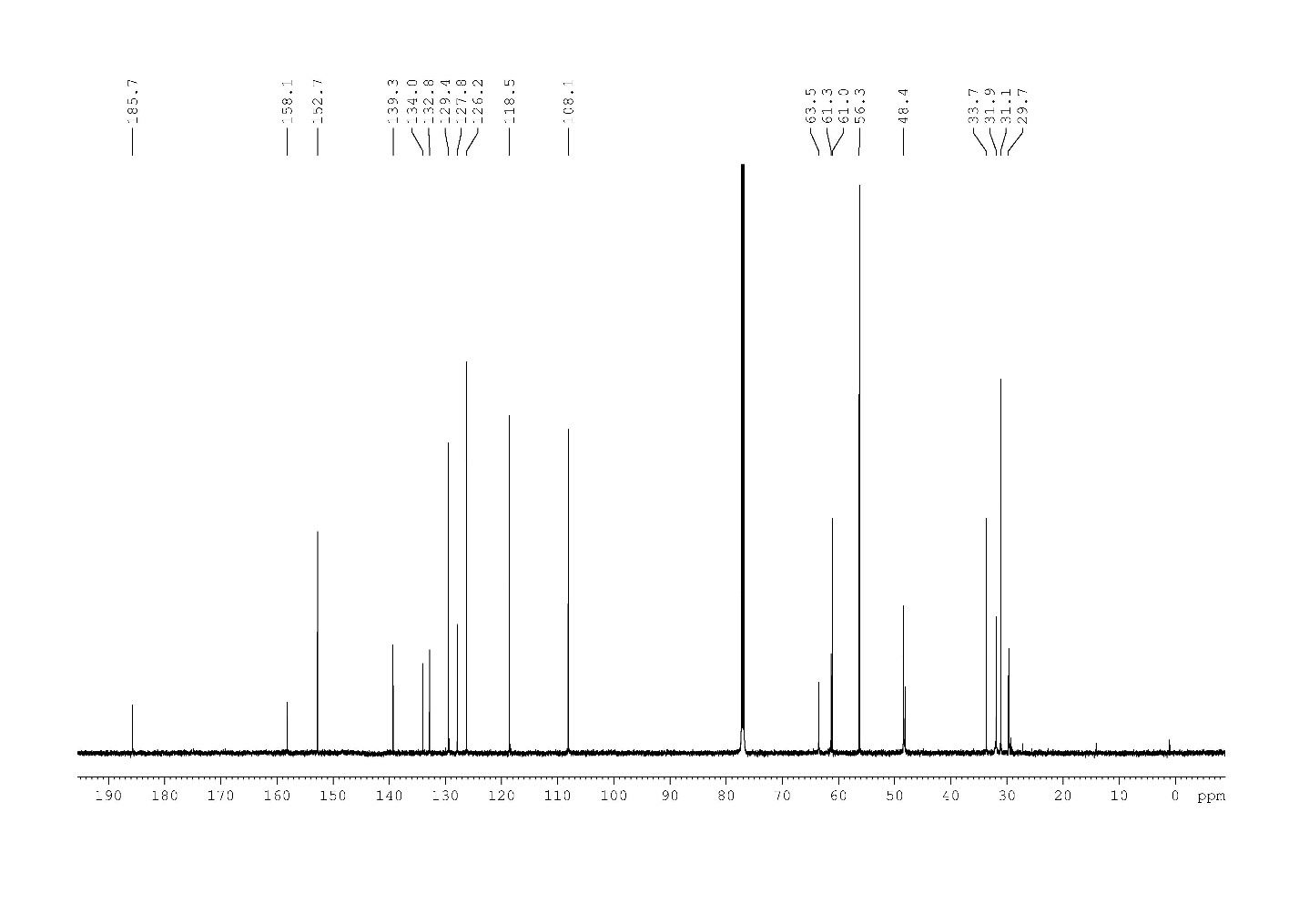


**
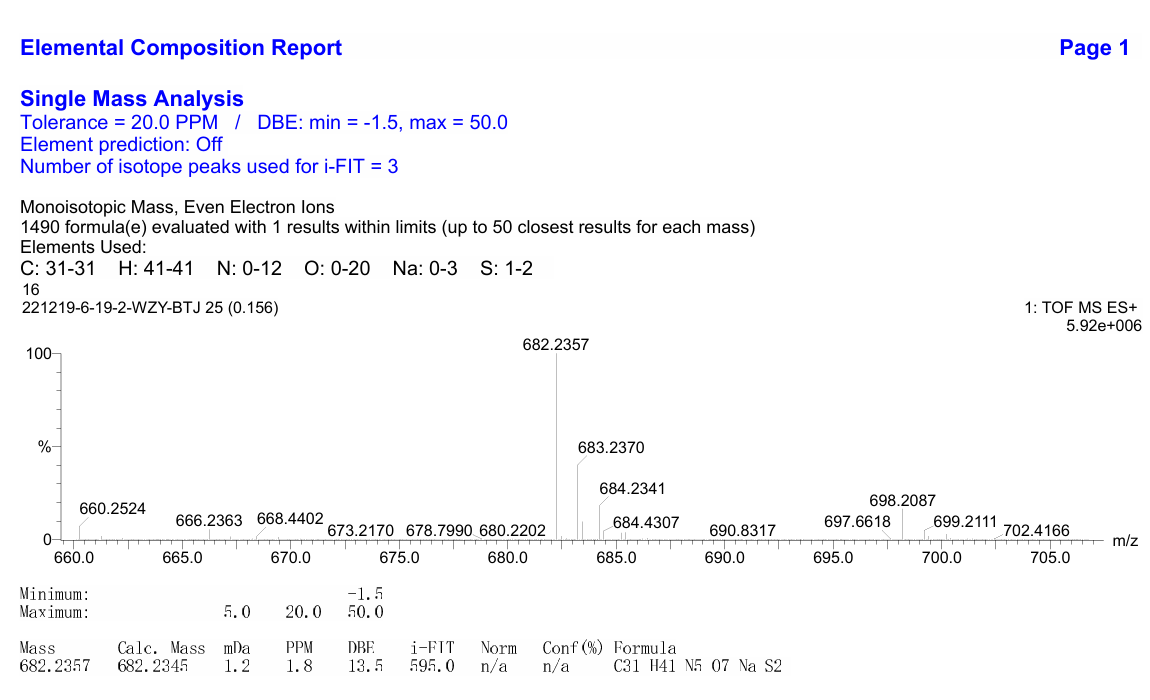
Fig. S16.** ¹H NMR spectrum (CDCl_3_), ¹³C NMR spectrum (CDCl_3_), and HRMS spectrum (ESI) of compound **10a**.

[M+Na]^+^


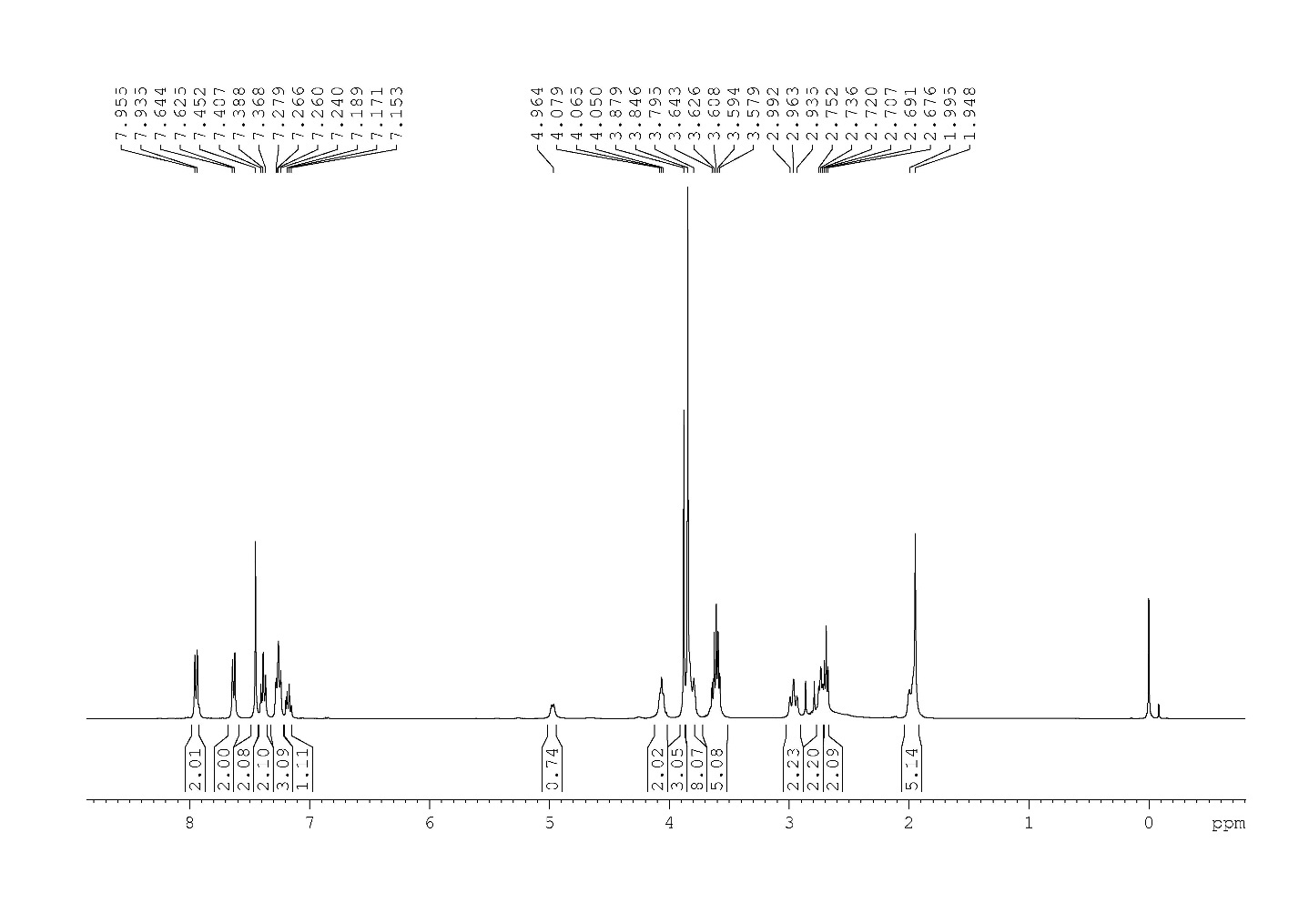


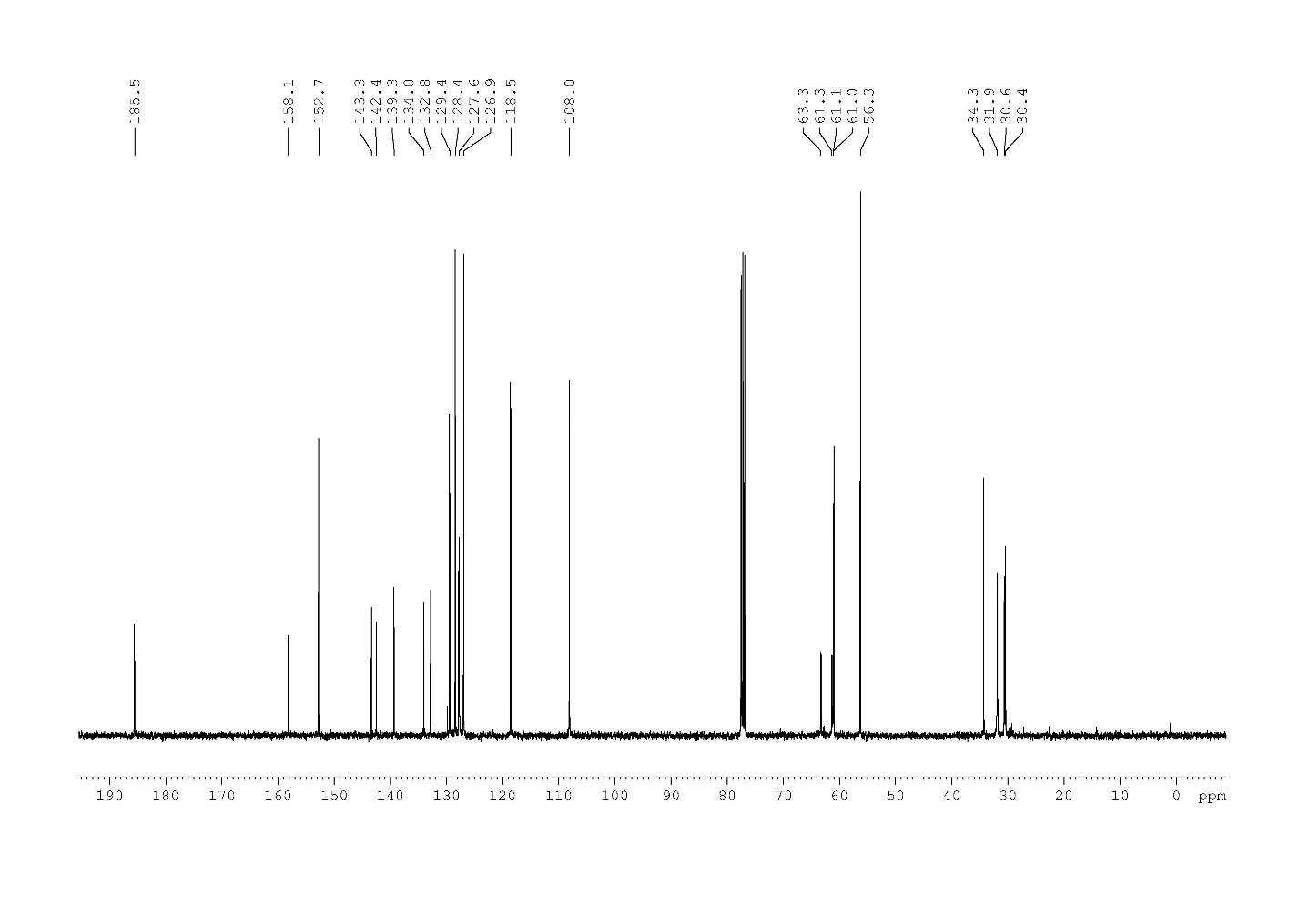


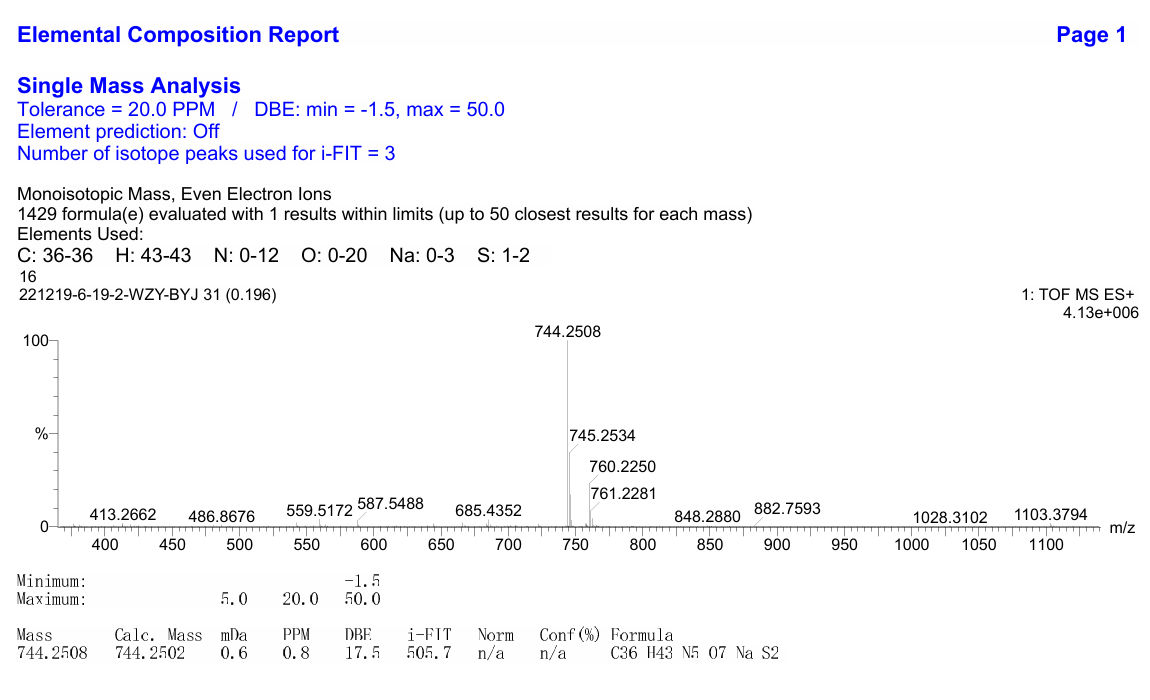


[M+Na]^+^

**Fig. S17.** ¹H NMR spectrum (CDCl_3_), ¹³C NMR spectrum (CDCl_3_), and HRMS spectrum (ESI) of compound **10b**.


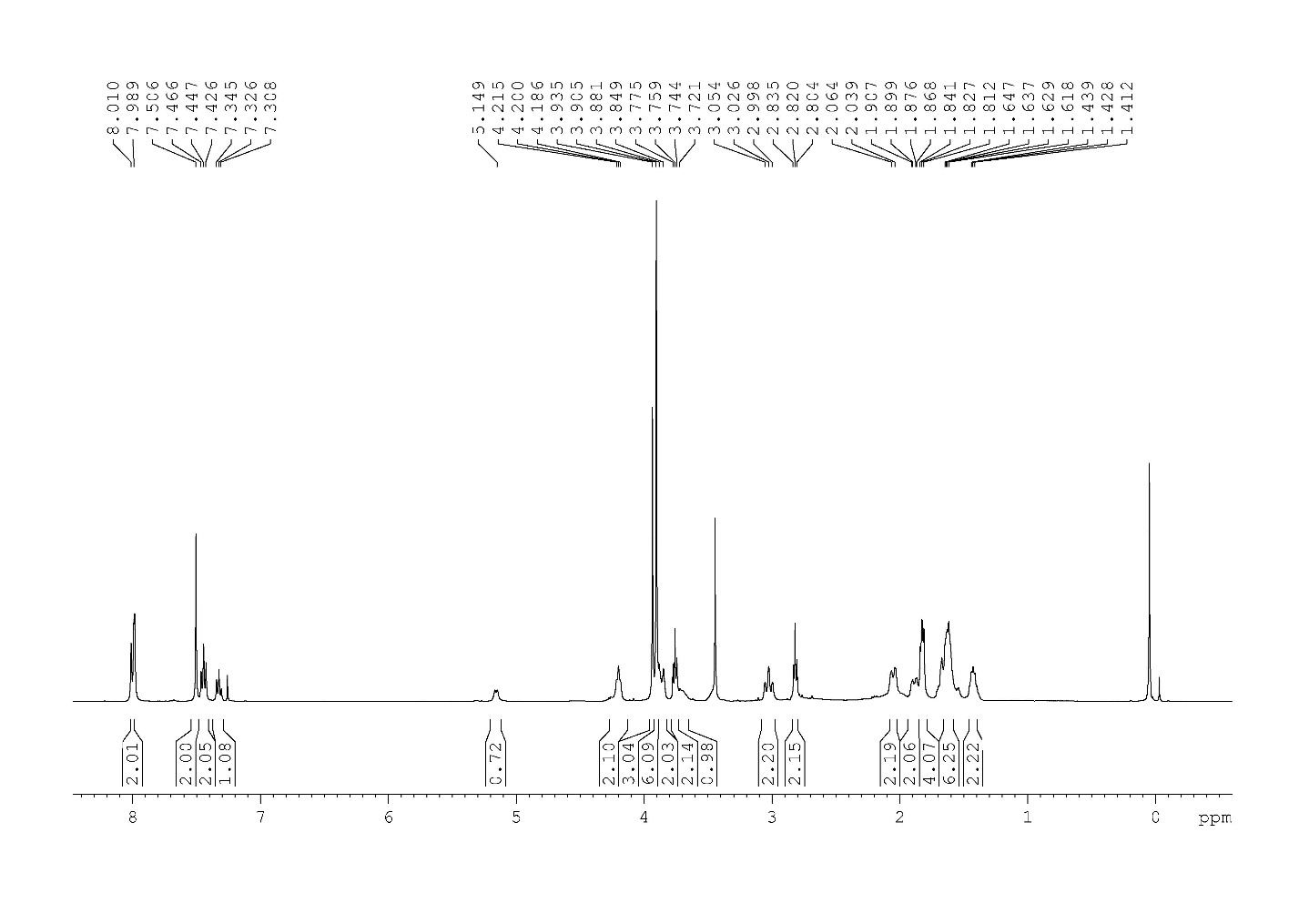


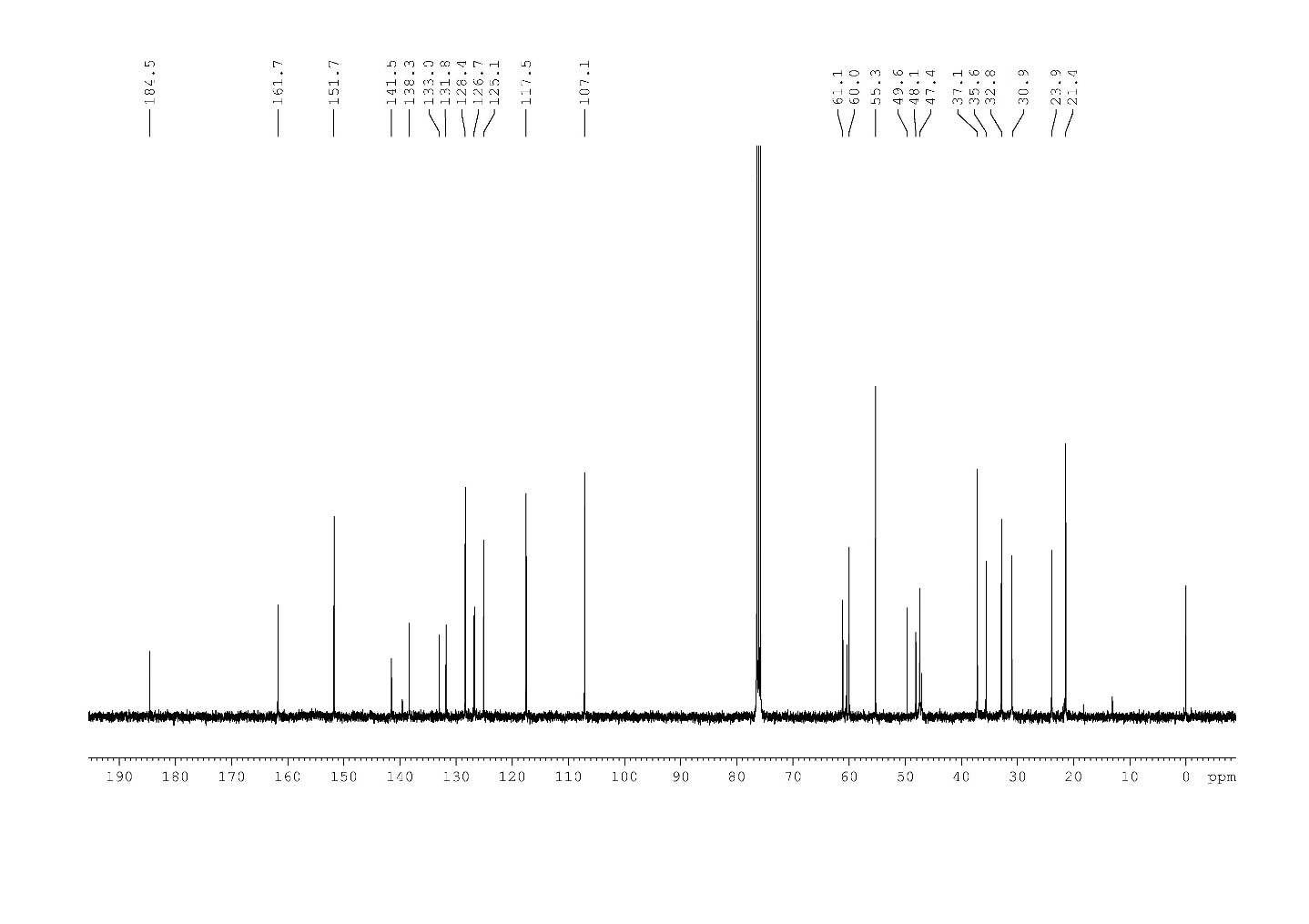


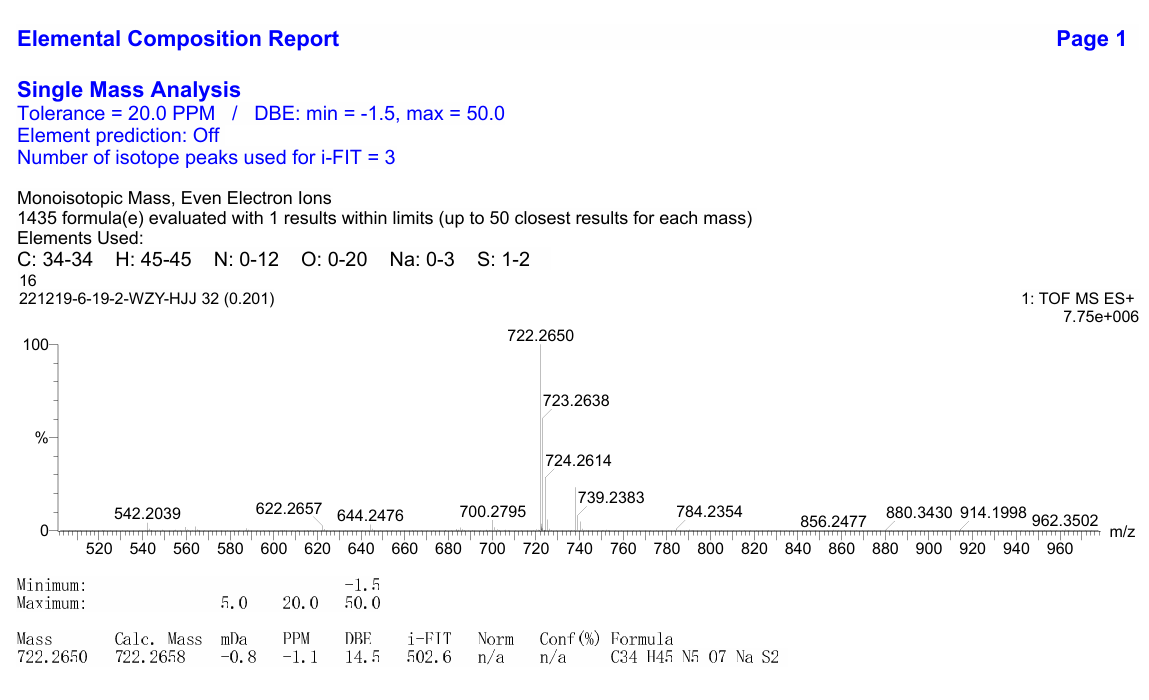


[M+Na]^+^

**Fig. S18.** ¹H NMR spectrum (CDCl_3_), ¹³C NMR spectrum (CDCl_3_), and HRMS spectrum (ESI) of compound **10c**.

**
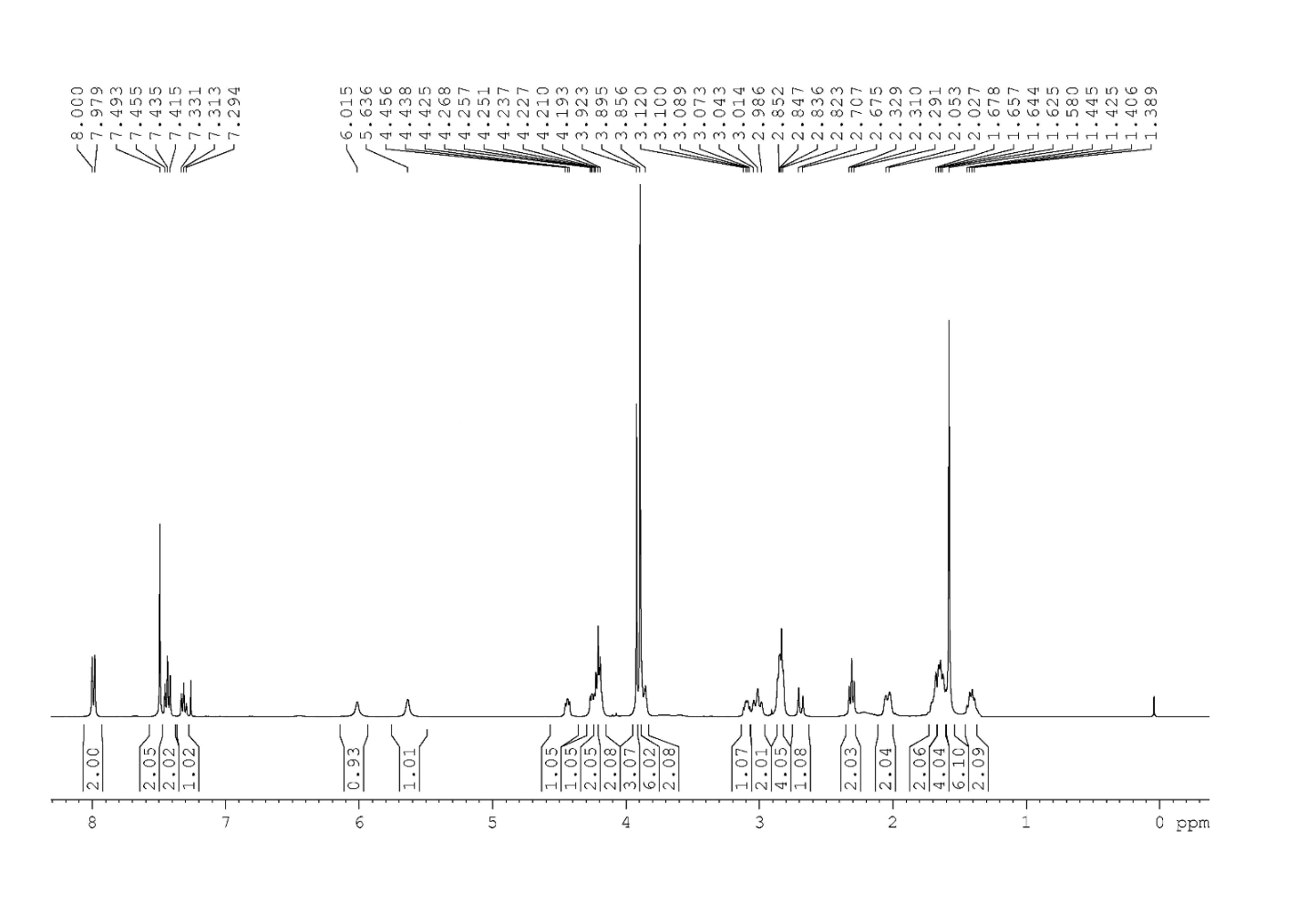

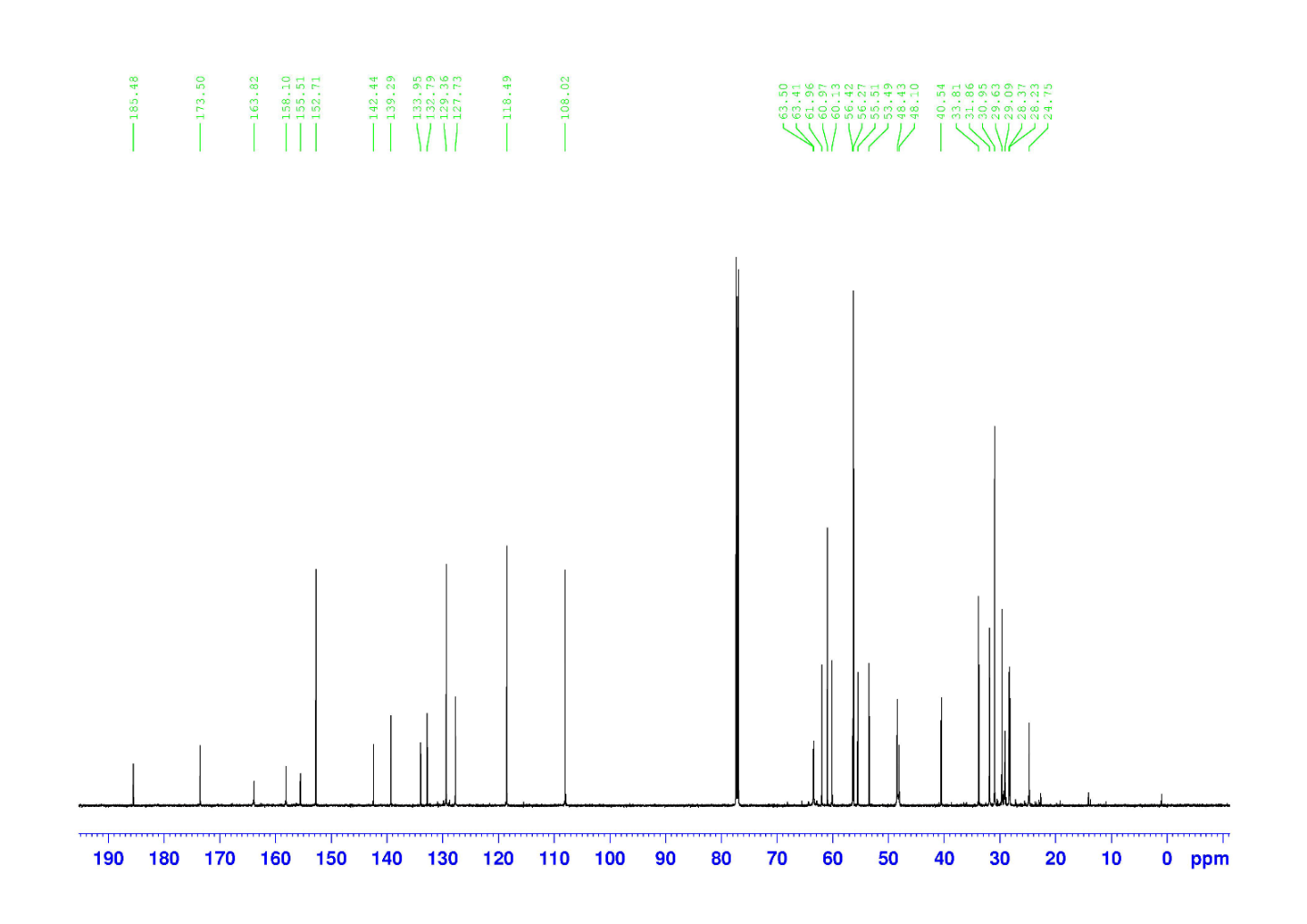
**

**
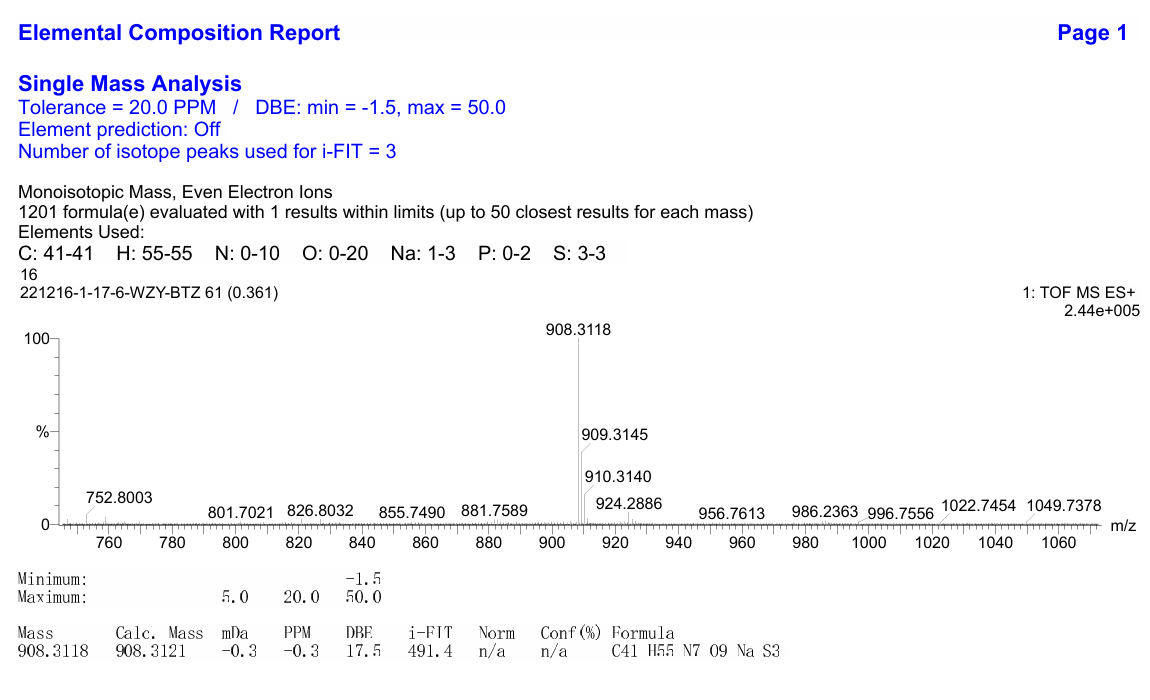
Fig. S19.** ¹H NMR spectrum (CDCl_3_), ¹³C NMR spectrum (CDCl_3_), and HRMS spectrum (ESI) of compound **BTW1**.

[M+Na]^+^

**
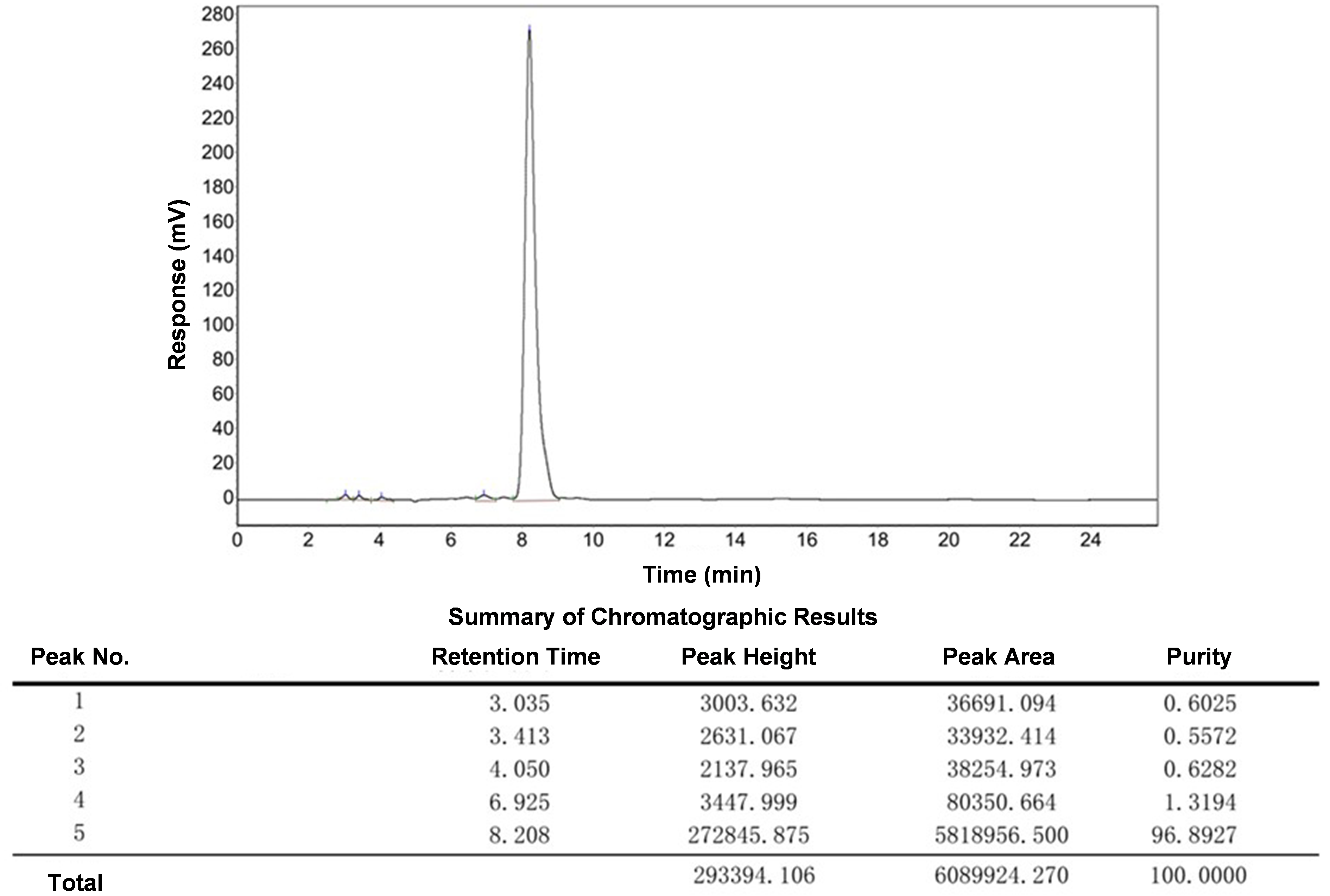
**

**Fig. S20.** HPLC chromatograms of **BTW1** for purity analysis (detection at 254 nm).

**
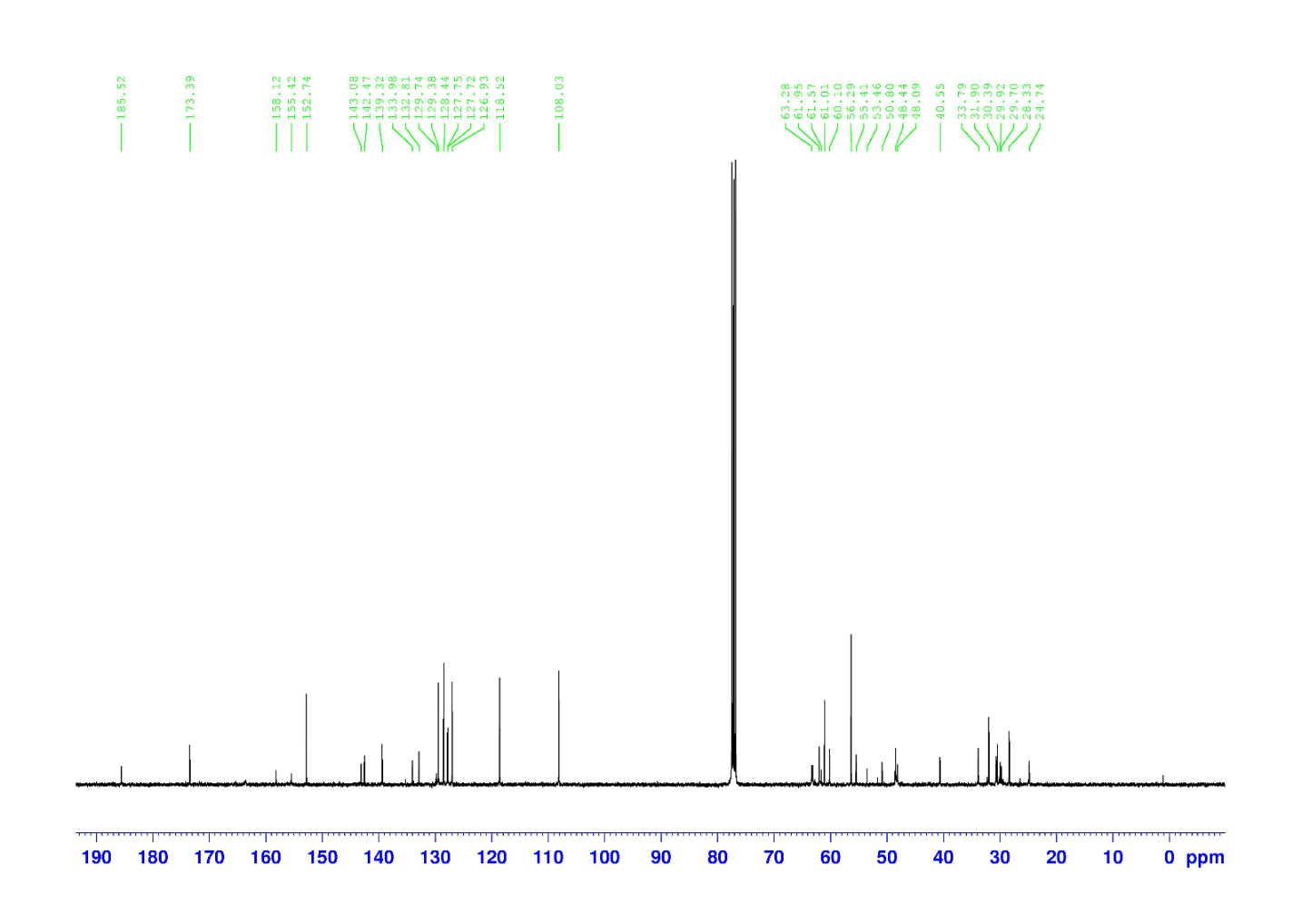

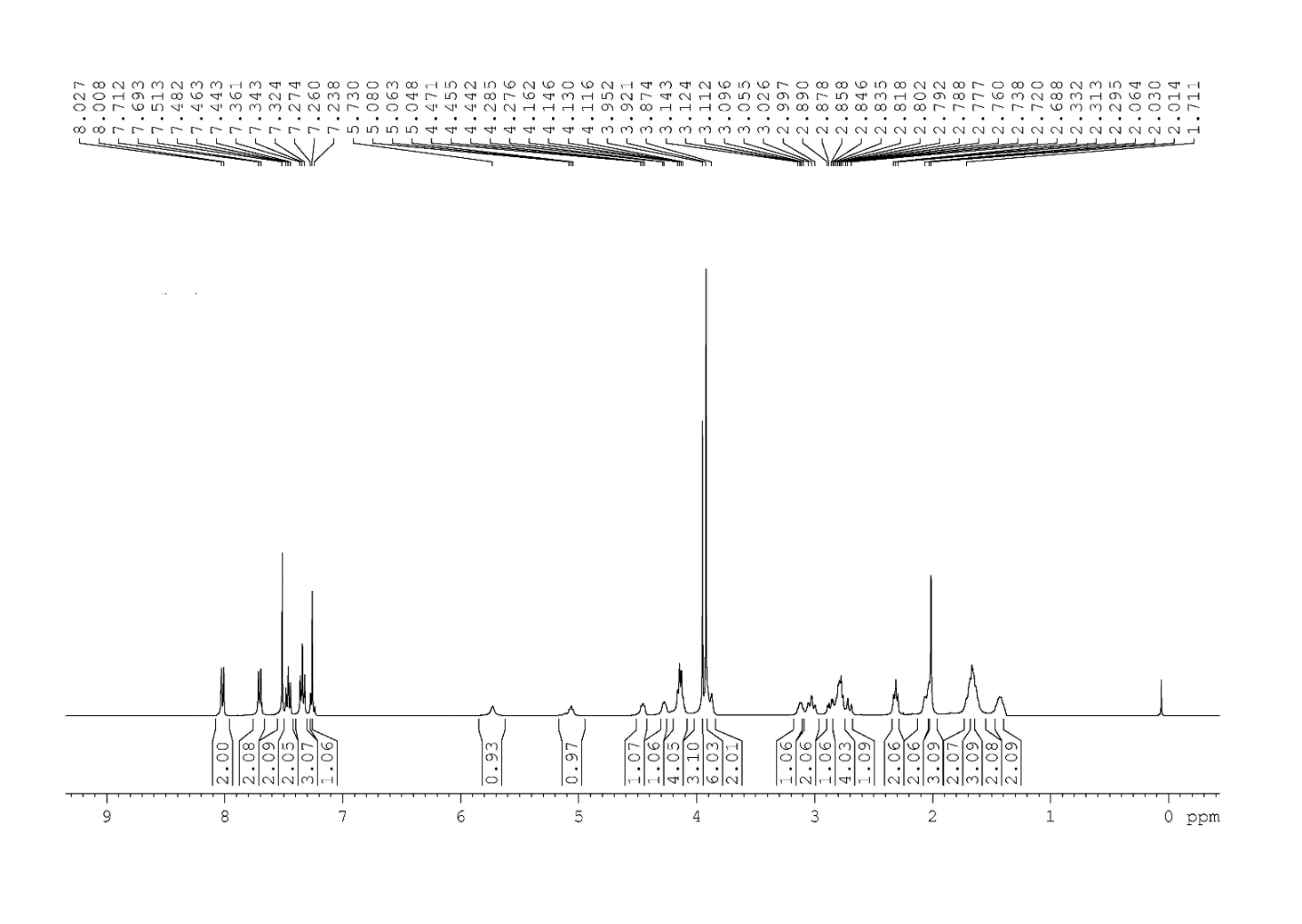
**

**
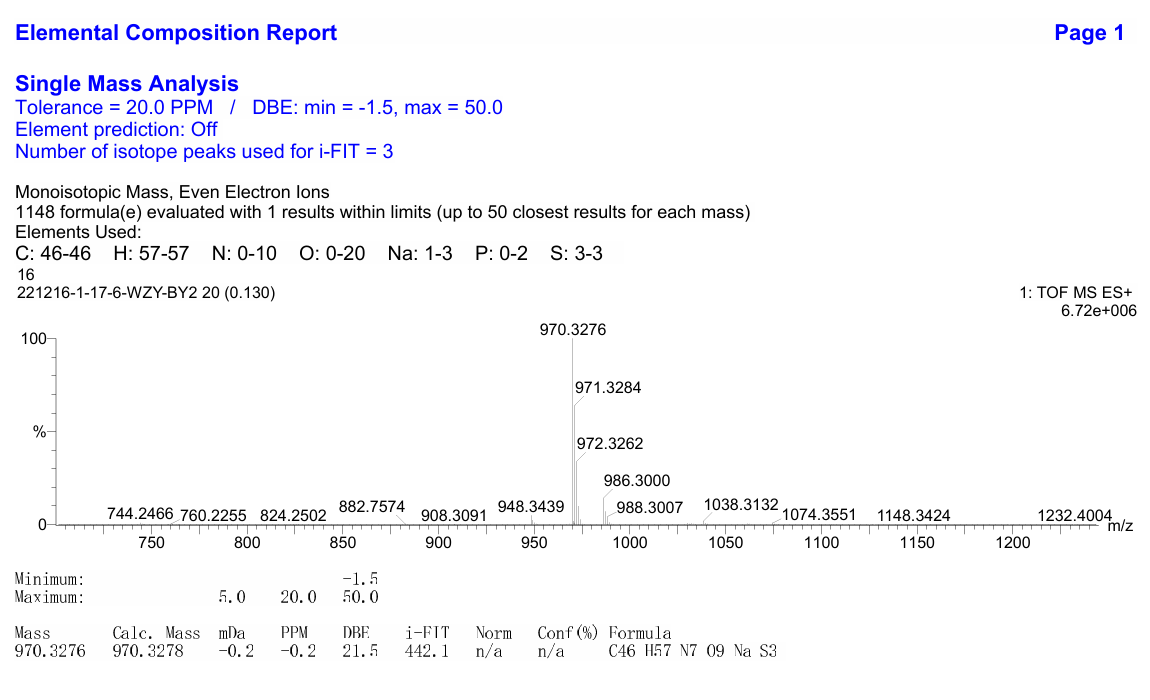
Fig. S21.** ¹H NMR spectrum (CDCl_3_), ¹³C NMR spectrum (CDCl_3_), and HRMS spectrum (ESI) of compound **BTW2**.

[M+Na]^+^

**
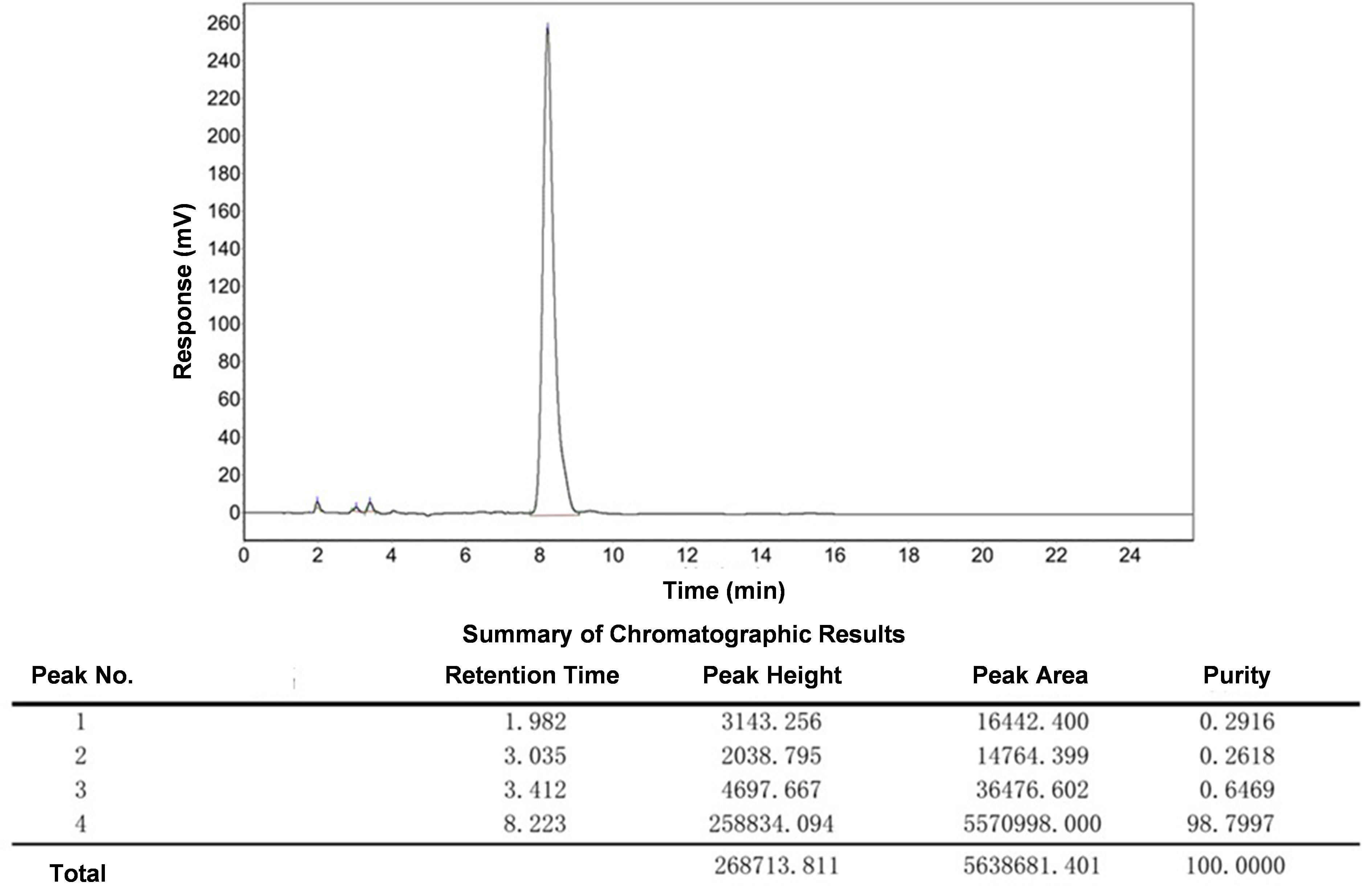
**

**Fig. S22.** HPLC chromatograms of **BTW2** for purity analysis (detection at 254 nm).

**
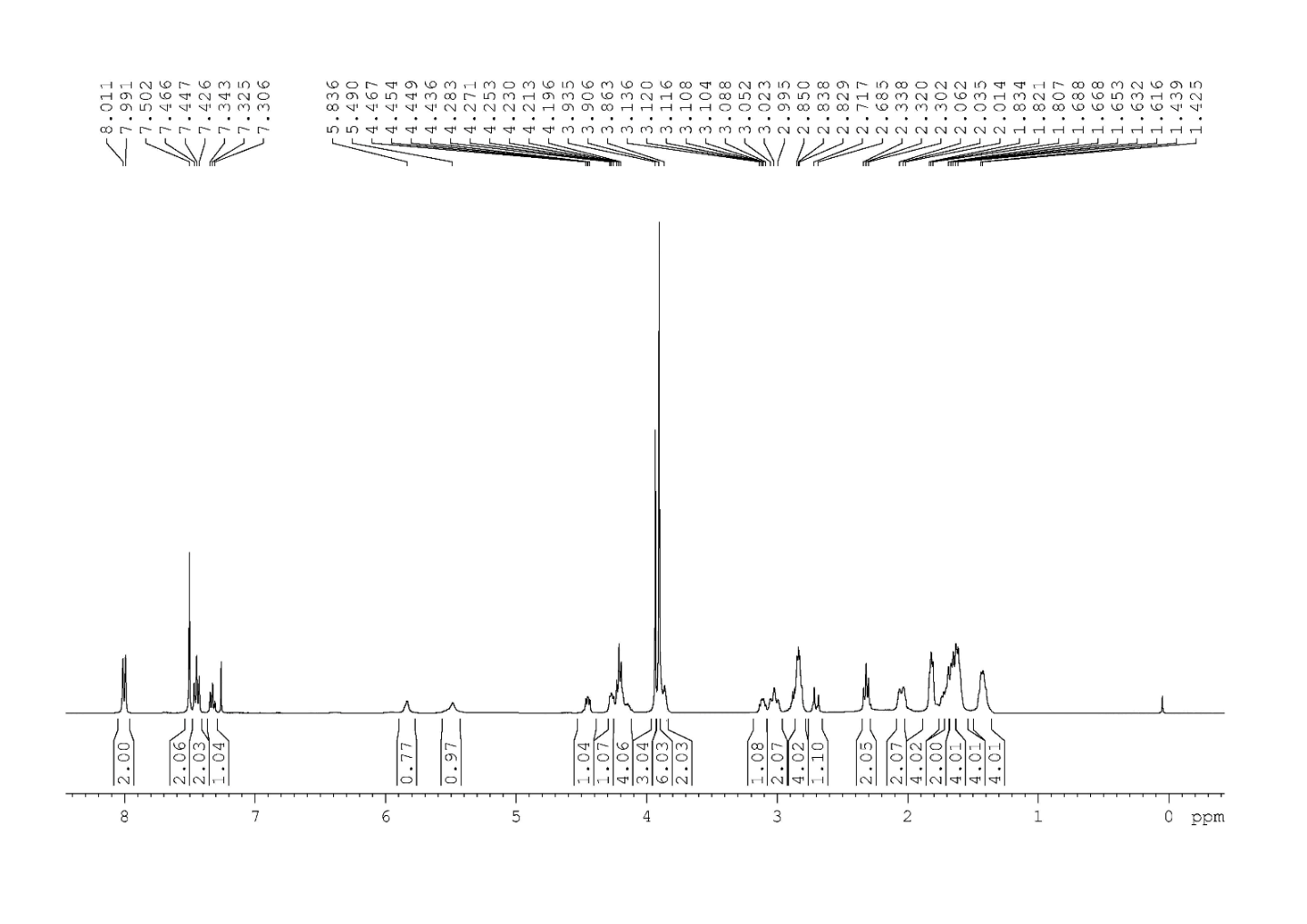
**
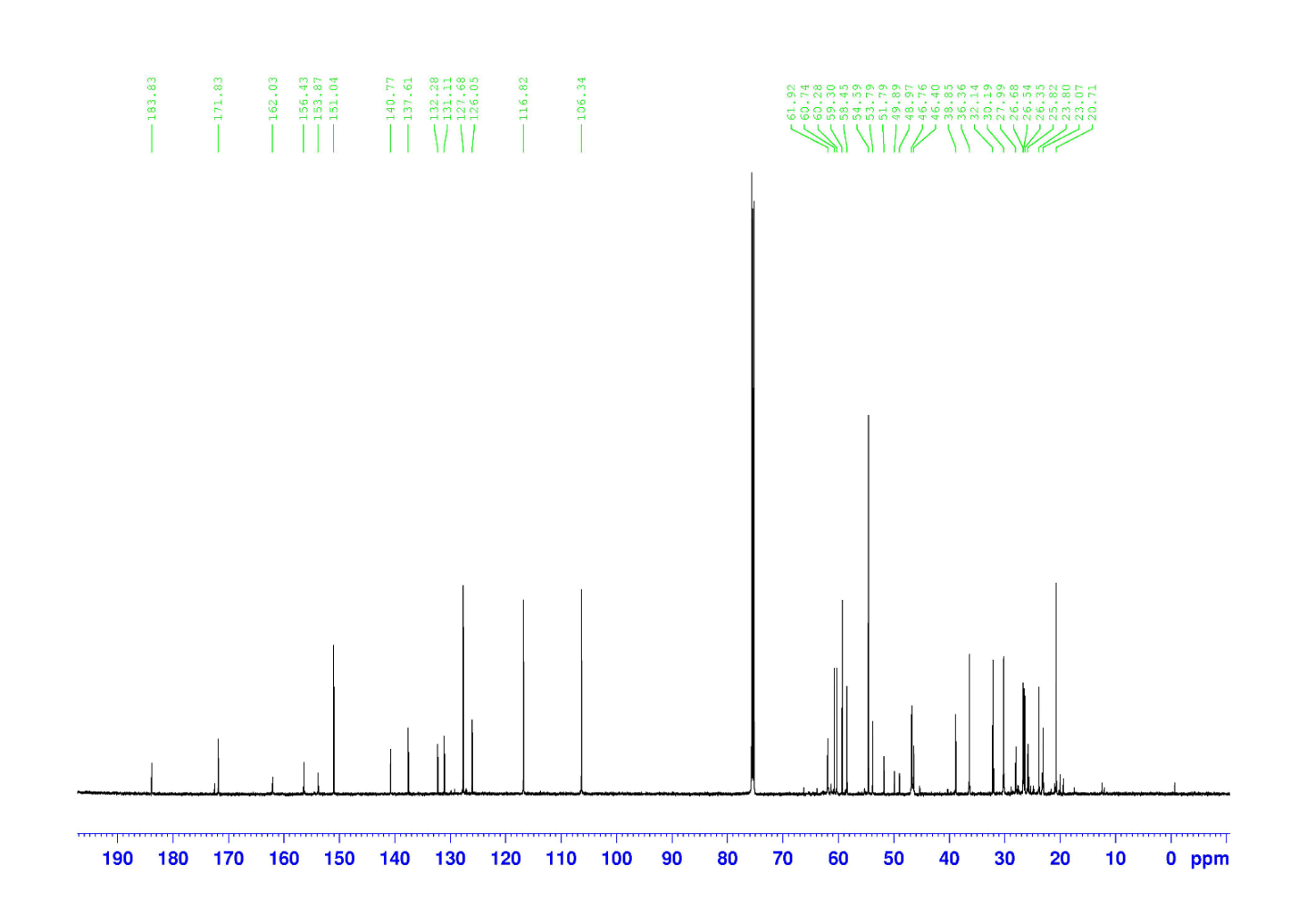


**
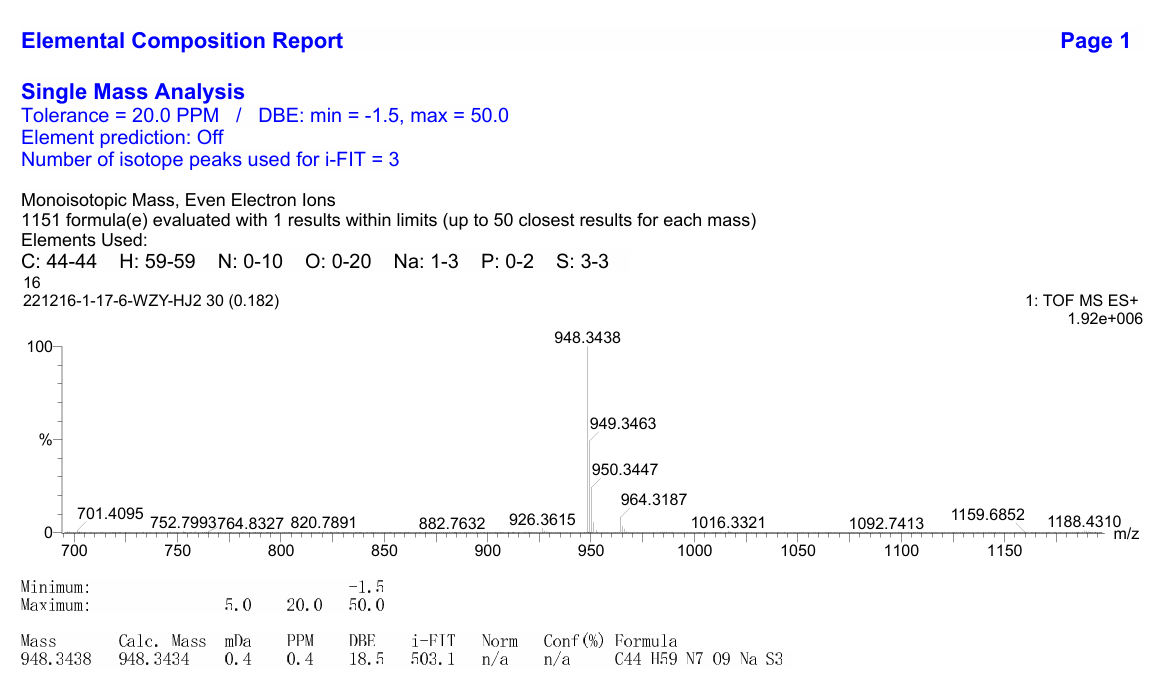
Fig. S23.** ¹H NMR spectrum (CDCl_3_), ¹³C NMR spectrum (CDCl_3_), and HRMS spectrum (ESI) of compound **BTW3**.

[M+Na]^+^

**
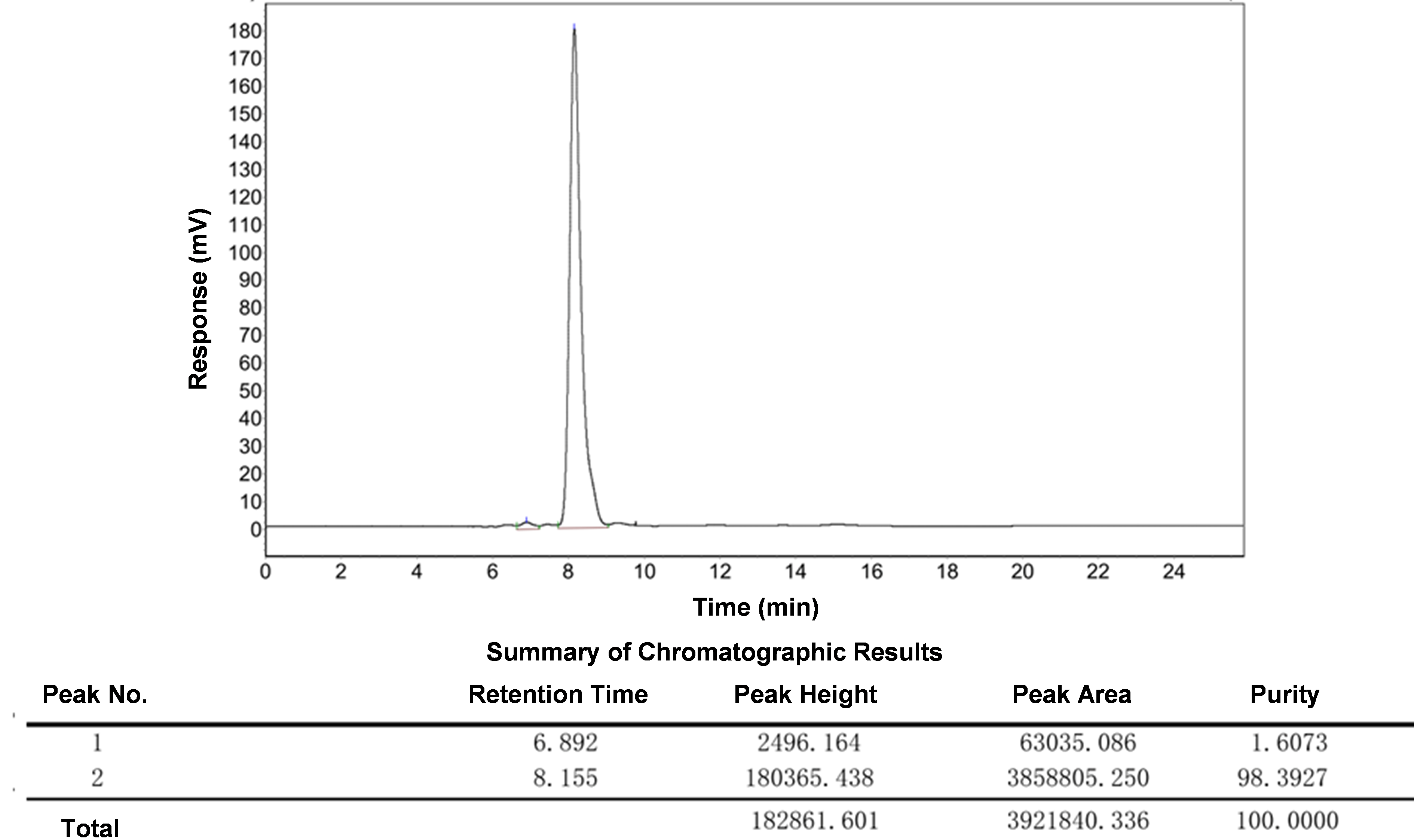
**

**Fig. S24.** HPLC chromatograms of **BTW3** for purity analysis (detection at 254 nm).

**
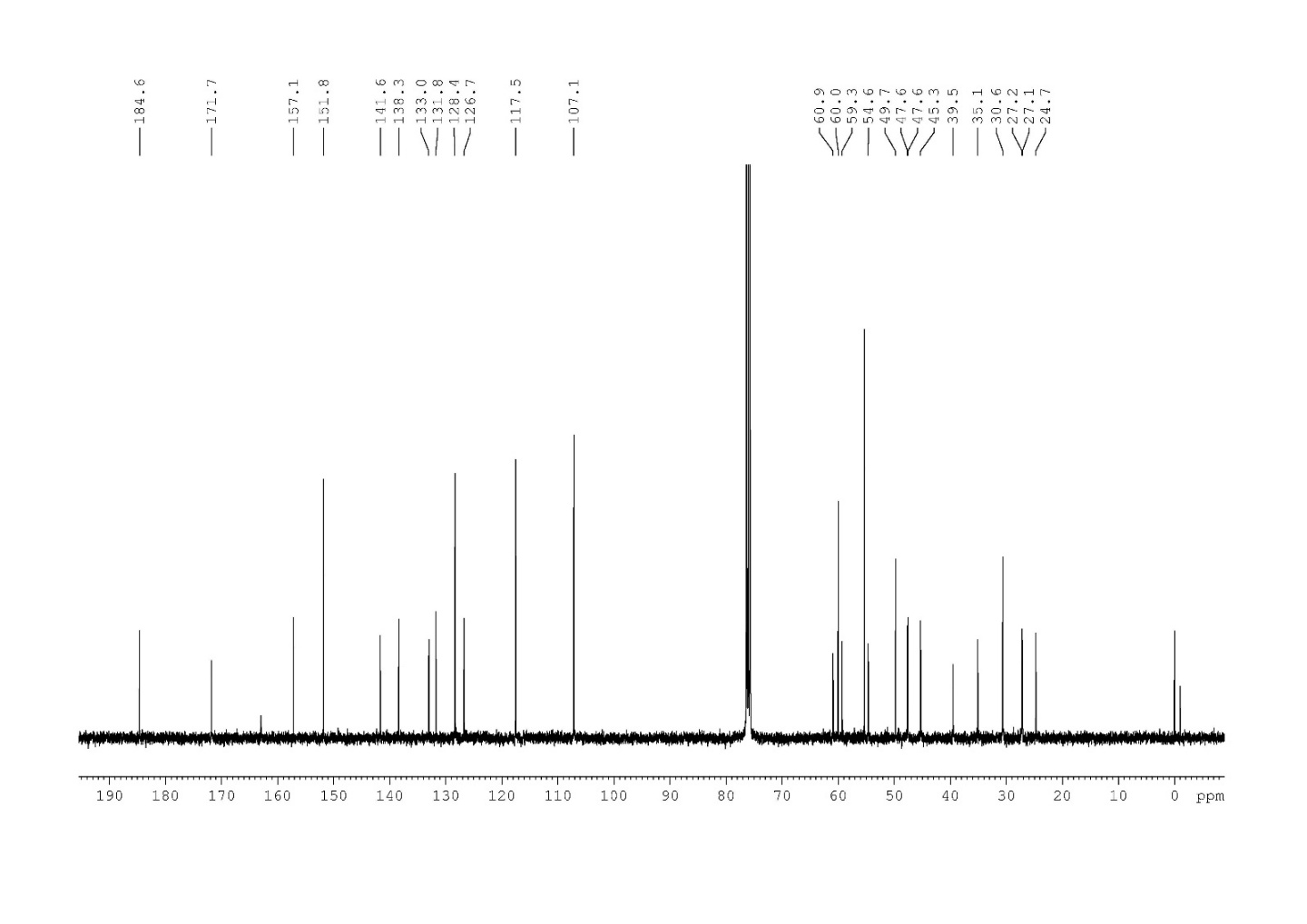

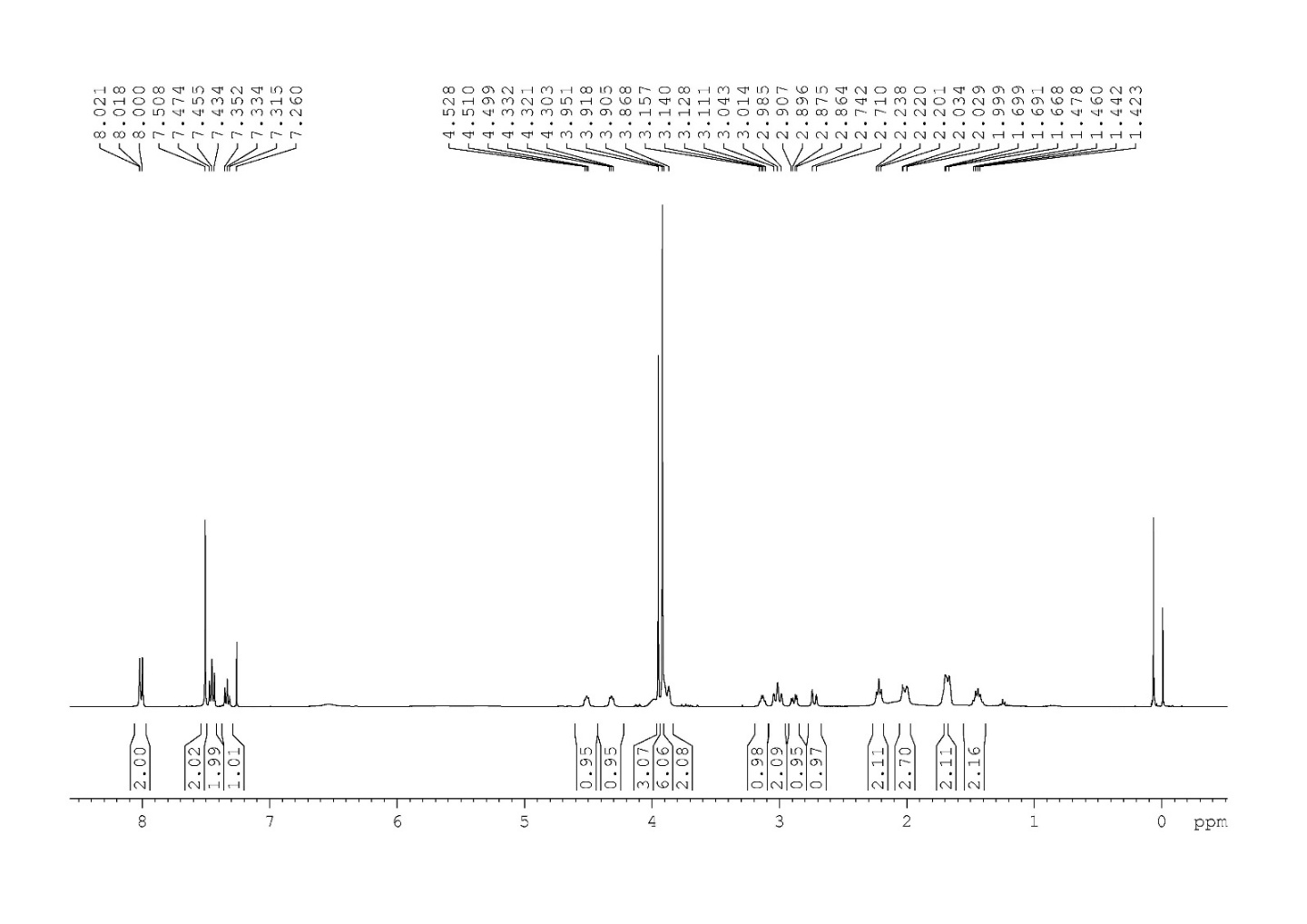
**

**
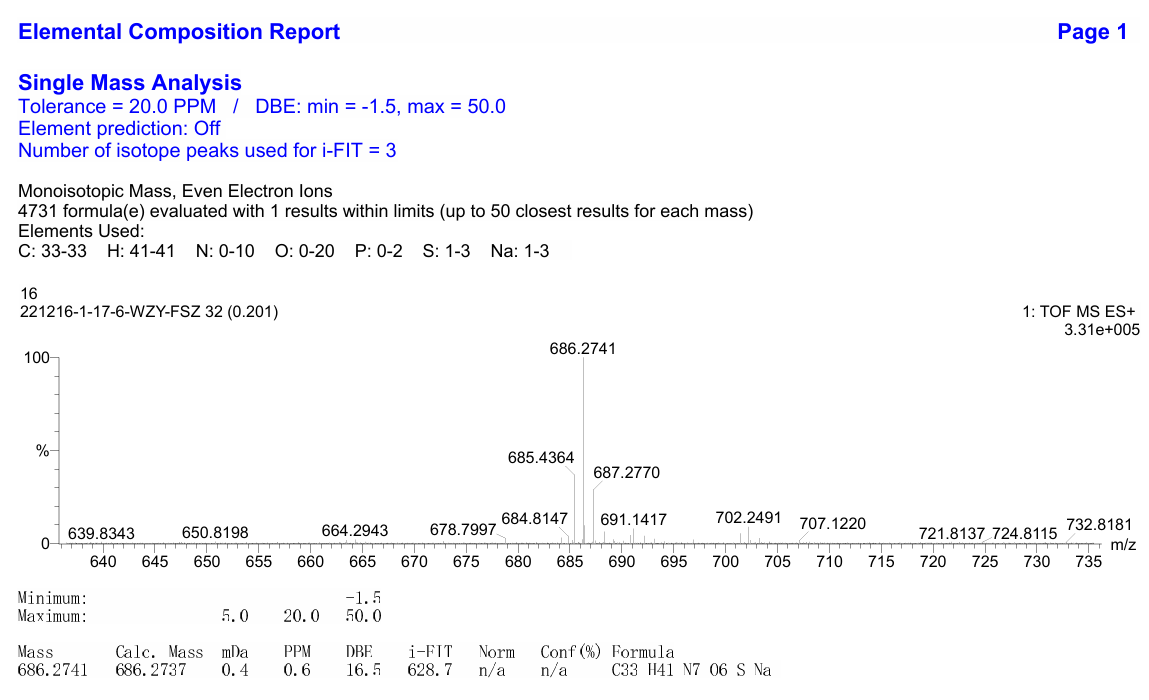
Fig. S25.** ¹H NMR spectrum (CDCl_3_), ¹³C NMR spectrum (CDCl_3_), and HRMS spectrum (ESI) of compound **BW**.

[M+Na]^+^

**
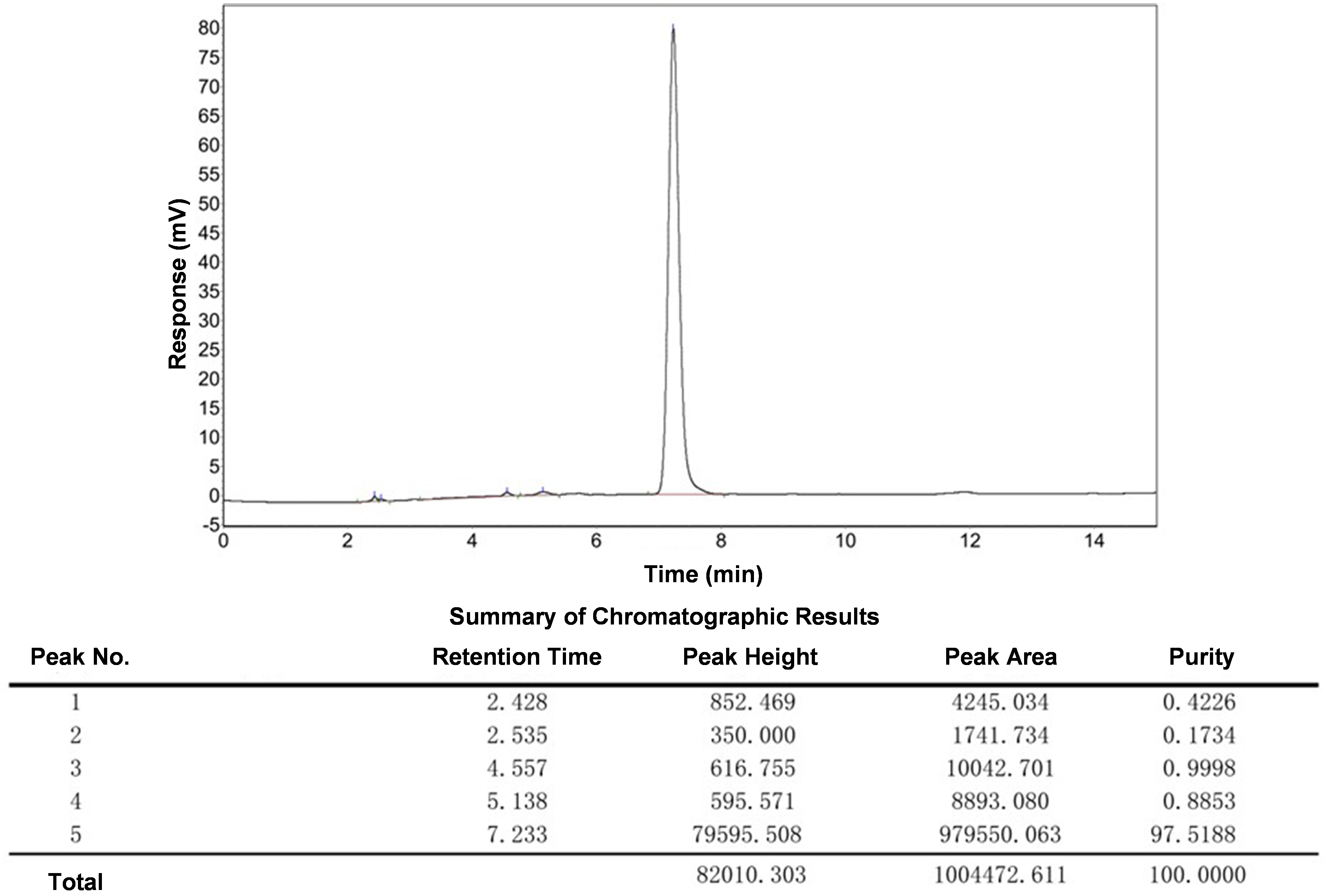
**

**Fig. S26.** HPLC chromatograms of **BW** for purity analysis (detection at 254 nm).

**Cytotoxicity of IR-808 in various human cell lines.**

**
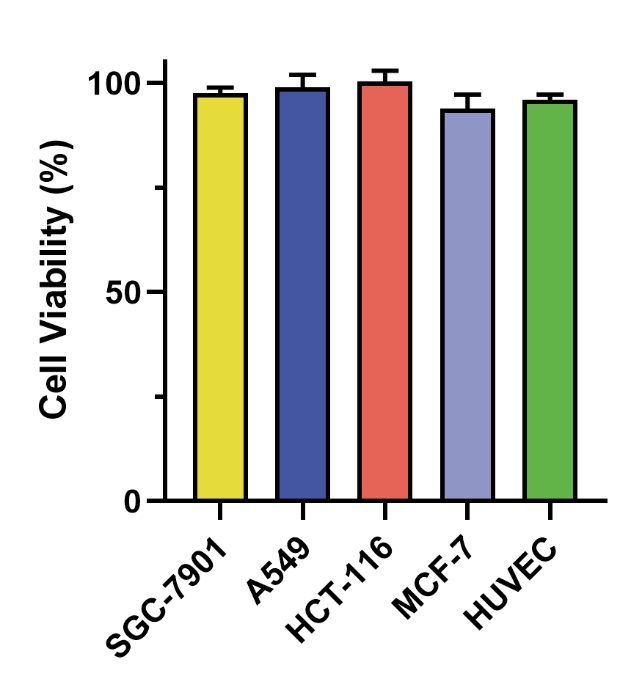
**

**Fig. S27.** Viability of five human cell lines after 72 h incubation with 10 μM IR-808 at 37 °C. Cell viability was determined by MTT assay (n = 3).

**Tubulin polymerization**

To assess whether the antiproliferative activity of W436 involved microtubule targeting, paclitaxel (a microtubule stabilizer) and colchicine (a microtubule destabilizer) were used as negative and positive controls, respectively, in an in vitro tubulin polymerization assay. As shown in Fig. S2, paclitaxel significantly promoted microtubule polymerization (indicated by increased fluorescence intensity), whereas both **W436** and colchicine effectively inhibited polymerization (indicated by reduced fluorescence intensity compared to the control). Notably, **W436** exhibited dose-dependent inhibition, suggesting that tubulin is likely its primary molecular target.


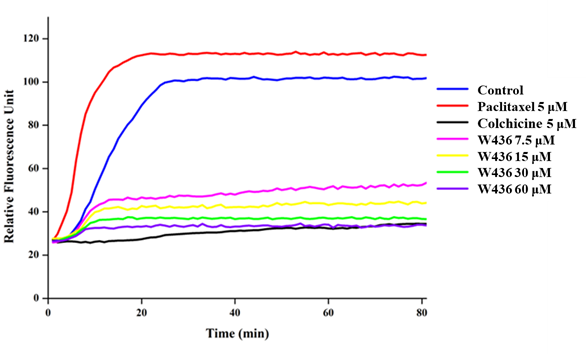


**Fig. S28.** Effects of compound **W436** on tubulin polymerization.

**Immunofluorescence studies**

To further investigate the impacts of **W436** on intracellular microtubules, we conducted immunofluorescence assay. MCF-7 cells were treated with **W436** and colchicine at their respective 2-fold IC_50_ concentrations, followed by staining with a tubulin antibody. The results depicted in **Fig. S3** reveal that treatment with compound **W436** led to alterations in cell morphology and disruption of the microtubule network, consistent with the effects observed with positive control drugs. These findings provide evidence that compound **W436** has the ability to destabilize microtubules.


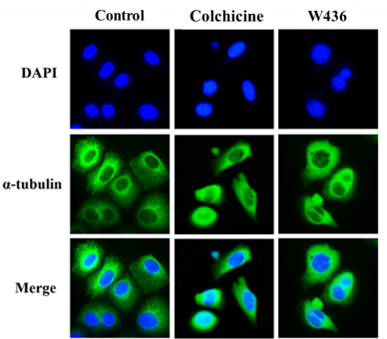


**Fig. S29.** Immunofluorescent staining of microtubule and nucleus in MCF-7 cells.

**Cell cycle analysis**

Given that microtubule inhibitors typically induce G₂/M phase arrest, we examined effects of **W436** on the cell cycle to validate its tubulin-targeting mechanism. MCF-7 cells treated with **W436** or colchicine (both at 2× IC₅₀ concentrations) were analyzed by flow cytometry. As shown in Fig. S4, both compounds induced time-dependent G₂/M arrest: after 12 h, the G₂/M population increased from 17.2% to 83.6% (**W436**) and from 22.1% to 77.3% (colchicine). Concurrently, the SubG₁ fraction (indicative of apoptosis) progressively increased. These results demonstrate that W436 effectively arrests cells at G₂/M phase and initiates apoptosis.


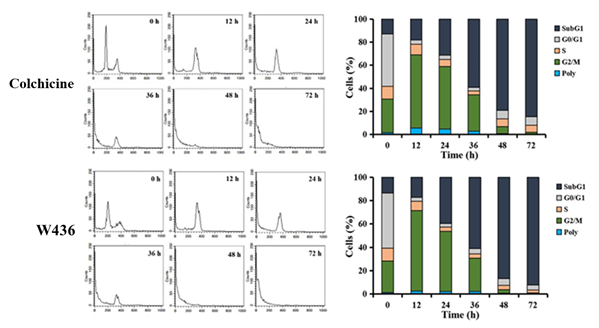


**Fig. S30.** Cell cycle distribution of MCF-7 cells.

**Molecular docking of W436 at the colchicine-binding site of tubulin.**

**
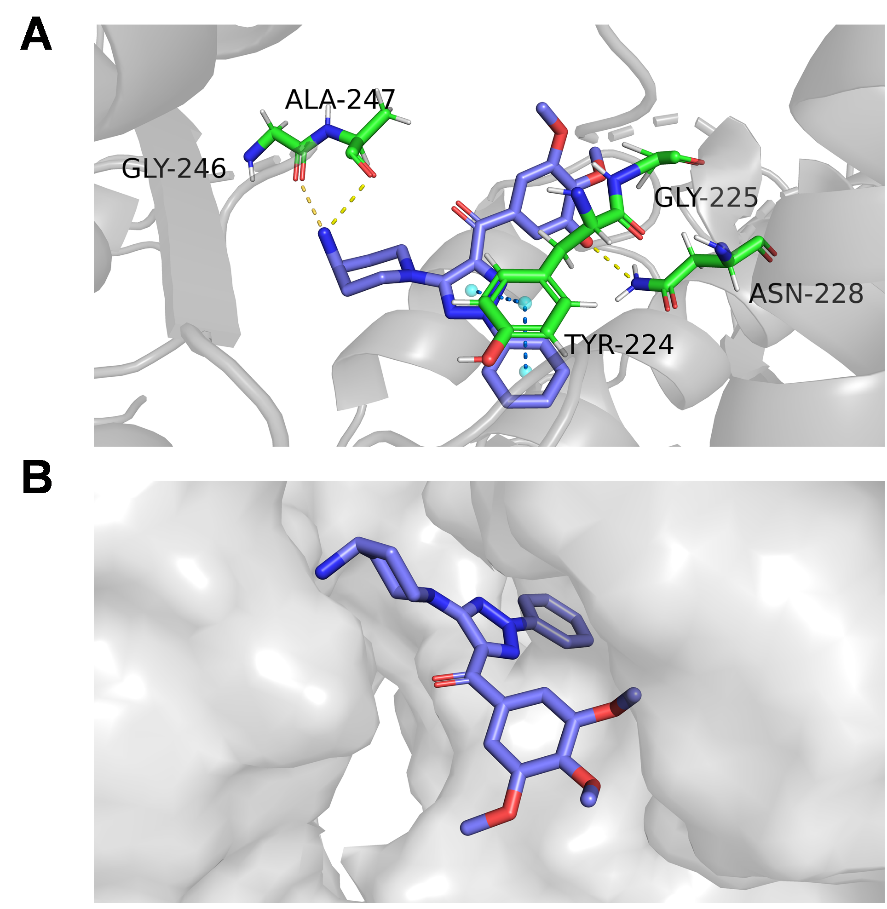
**

**Fig. S31.** (A) Binding modes for **W436** (blue) at the colchicine site. The yellow lines represent hydrogen bonds; the blue lines represent π-π stacking. (B) Surface representation of tubulin (gray) highlighting the spatial localization of **W436** (blue) within the colchicine-binding pocket.

**Stability of BTW prodrugs in cell culture medium**

**
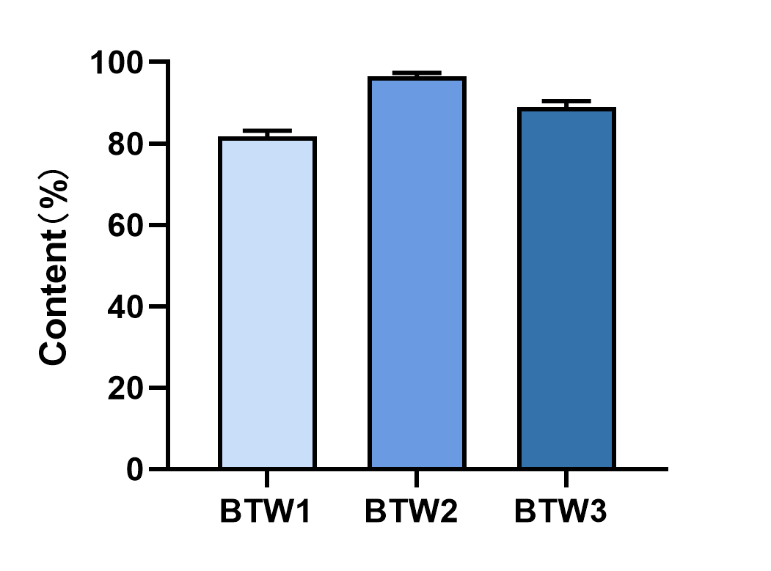
**

**Fig. S32.** Chemical stability evaluation of **BTW1**~**3** in cell culture medium maintained at 37°C for 72 hours.

**Size and zeta potential** **of BTW2 NPs**

**
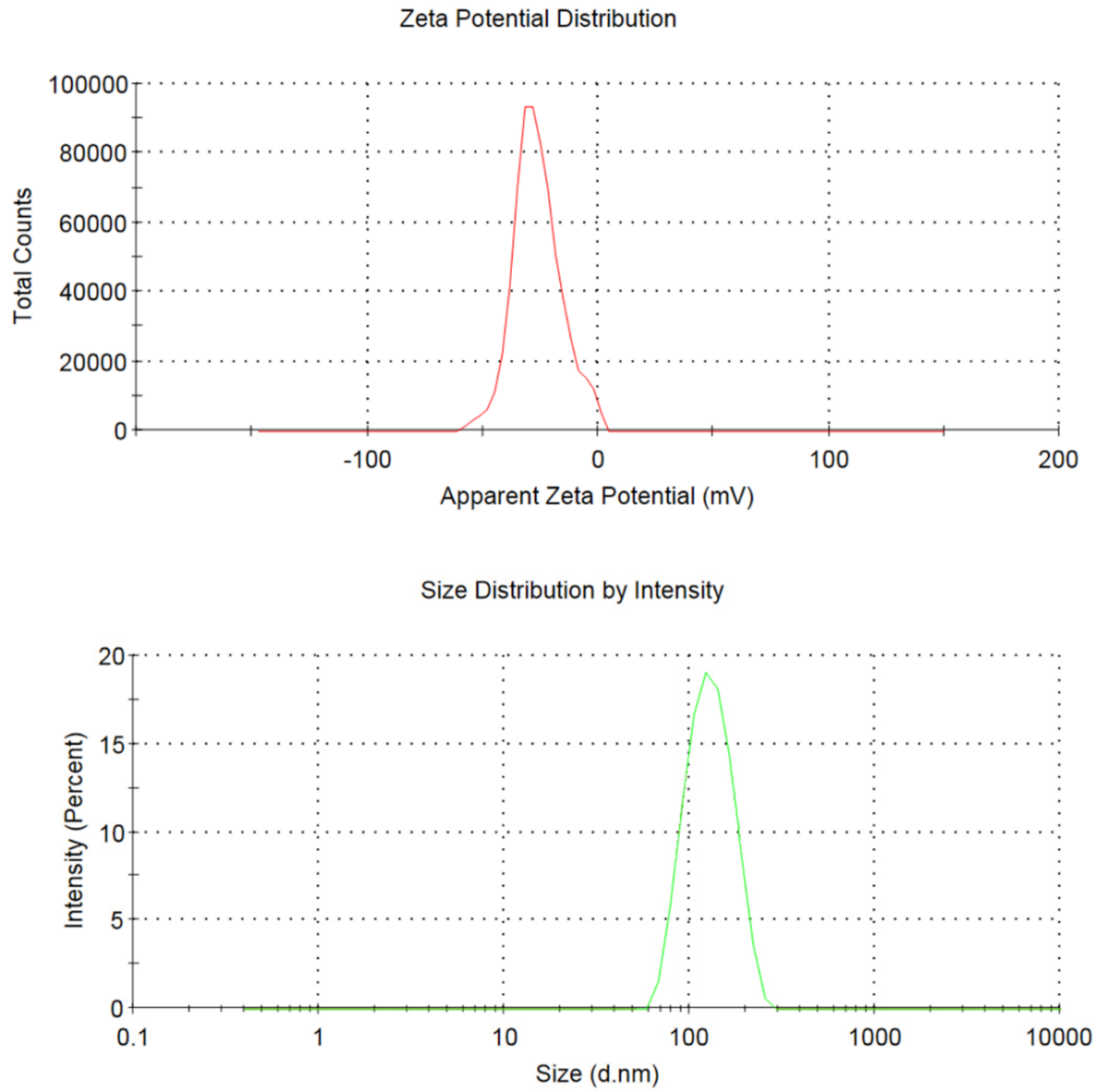
**

**Fig. S33.** Hydrodynamic sizes and Zeta potential of **BTW2** NPs.

**Stability studies of BTW2 NPs**

**
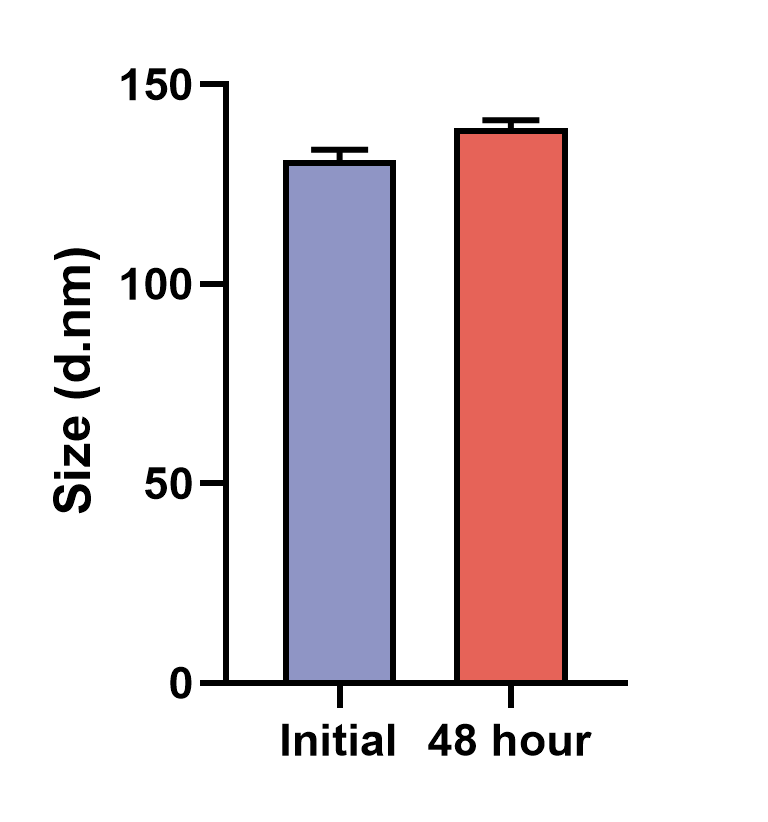

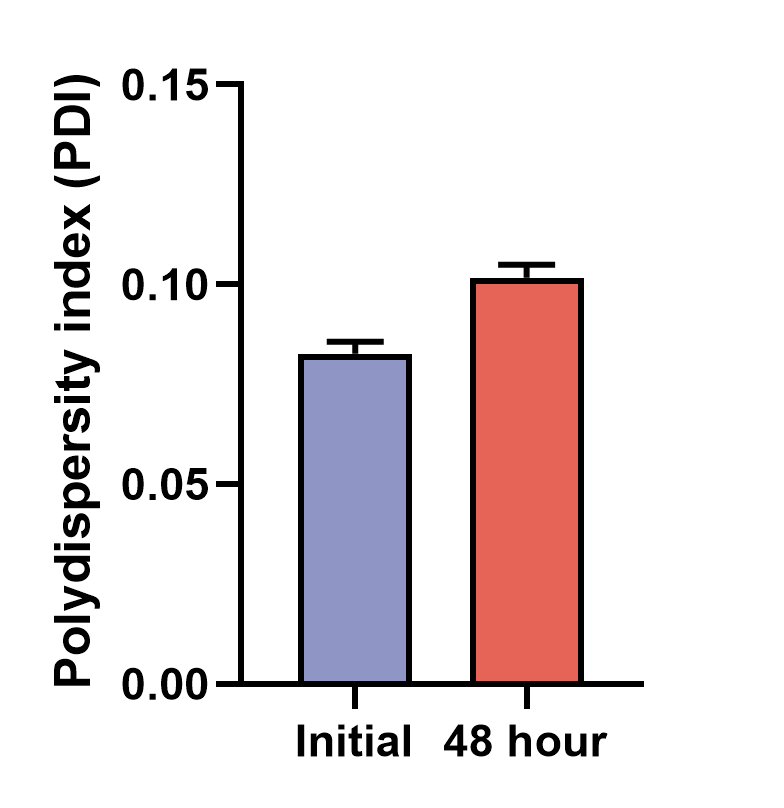
**

**Fig. S34.** Size and PDI variation of **BTW2** NPs after 48-h incubation in serum at 37 °C.

**
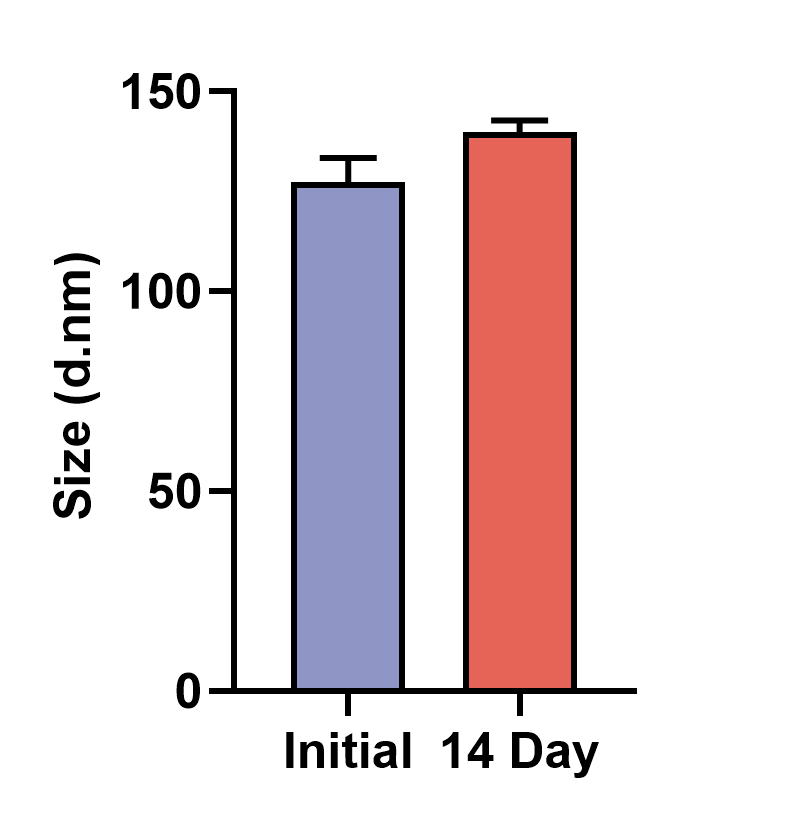

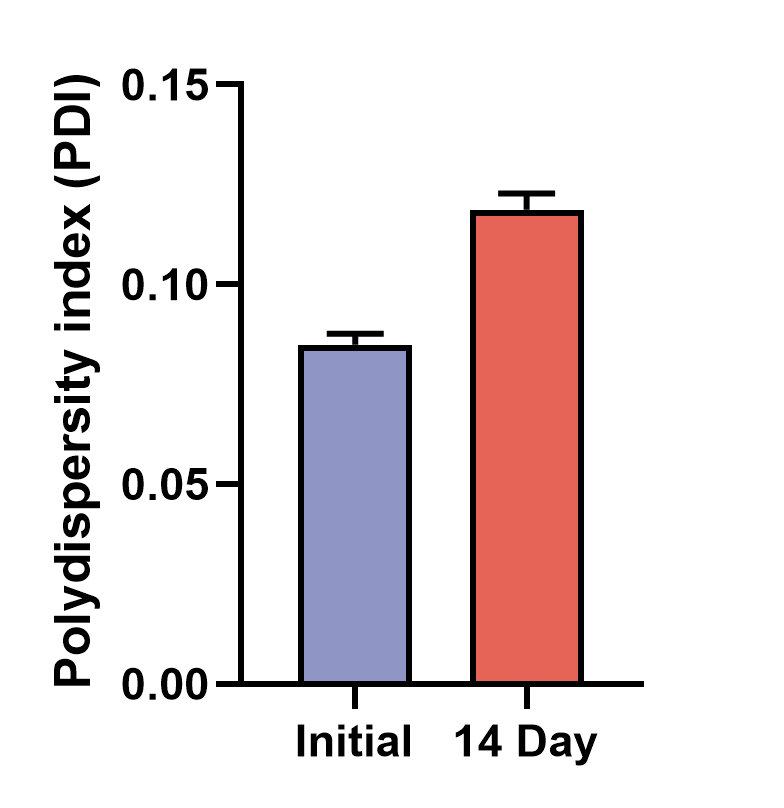
**

**Fig. S35.** Size and PDI variation of **BTW2** NPs after two-week storage at 4 °C.

**Fluorescence intensity in xenograft tumors and major organs at 48 h**


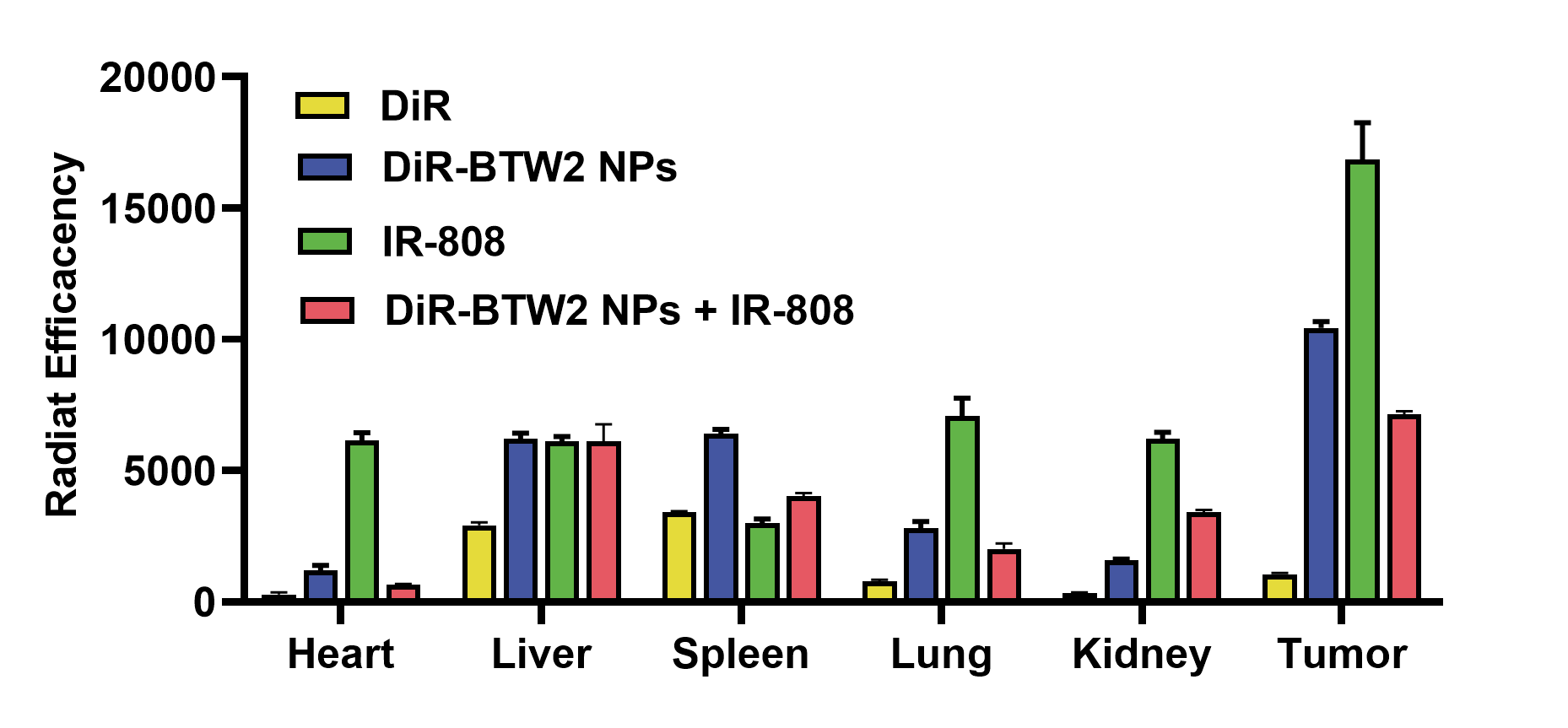


**Fig. S36.** quantitative results of xenograft tumor and major organs collected at 48 hours. (n = 3).

**In vivo and in vitro concordance evaluation of signal attenuation**

Assay performed in PBS/0.1 DMSO at 37°C using the IVIS Spectrum system (500 ms exposure). Fluorescence attenuation observed in DIR channel (Ex 748 nm/Em 780 nm) may result from competitive Förster Resonance Energy Transfer (FRET) and inner filter effects between IR-808 (acceptor) and DIR (donor) due to spectral overlap.

**
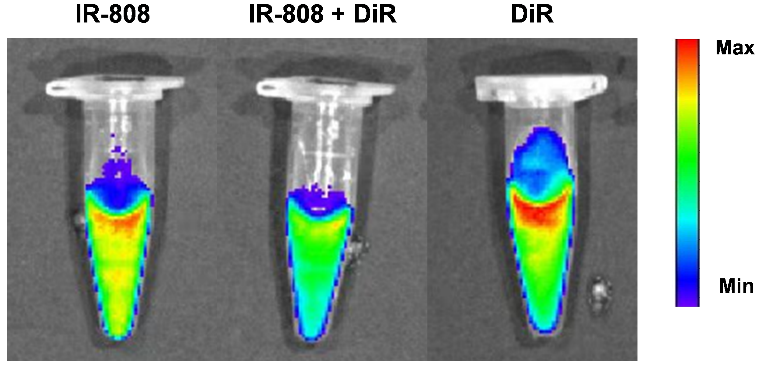
**

**Fig. S37**. NIR fluorescence imaging of IR-808 (0.1 μM), DiR (0.1 μM), and their equimolar mixture under 748 nm excitation.

**Table S1.** IC_50_ values of target compounds against five cancer cell lines and HUVECs.

| Compound | IC_50_ (μM) ± SD^[a]^ | | | | | | |
| --- | --- | --- | --- | --- | --- | --- | --- |
|  | SGC-7901 | A549 | HCT-166 | MCF-7 | 4T1 | HUVEC | selectivity index (HUVEC / MCF-7) |
| **W436** | 0.142 ± 0.016 | 0.130 ± 0.007 | 0.167 ± 0.025 | 0.099 ± 0.008 | 0.111 ± 0.012 | 0.269 ± 0.034 | 2.72 |
| **BTW1** | 0.961 ± 0.013 | 0.714 ± 0.024 | 1.98 ± 0.04 | 0.532 ± 0.014 | 0.513 ± 0.018 | 5.40 ± 0.15 | 10.15 |
| **BTW2** | 2.33 ± 0.12 | 1.49 ± 0.05 | 2.65 ± 0.09 | 1.18 ± 0.08 | 1.36 ± 0.03 | 20.79 ± 2.48 | 17.62 |
| **BTW3** | 1.22 ± 0.09 | 0.921 ± 0.042 | 2.06 ± 0.13 | 0.755 ± 0.042 | 1.10 ± 0.04 | 7.94 ± 0.27 | 10.51 |
| **BW** | > 30 | > 30 | > 30 | > 30 | > 30 | > 30 | - |
| **Colchicine^b^** | 0.098 ± 0.009 | 0.039 ± 0.010 | 0.019 ± 0.002 | 0.014 ± 0.001 | 0.024 ± 0.001 | 0.025 ± 0.008 | 1.79 |

[a] IC_50_: 50% inhibitive concentration after 72 h of drug exposure, as determined by using MTT assays.

[b] Colchicine was used as positive controls.

| Compound | Percent Remaining (%) | | | | | T_1/2_  (minute) | C_lint_  (mL/min/kg) |
| --- | --- | --- | --- | --- | --- | --- | --- |
|  | 0 min | 5 min | 15 min | 30 min | 45 min |  |  |
| **BTW2** | 100.00 | 96.51 | 87.07 | 74.01 | 64.20 | 68.89 | 79.22 |
| Ketanserin^[a]^ | 100.00 | 84.78 | 60.30 | 34.64 | 22.13 | 20.40 | 276.54 |

**Table S2.** Metabolic stability of **BTW2** in incubation with mouse liver microsomes.

[a] Historic data of ketanserin: T_1/2_ (Mouse) = 21.04 ± 5.23 min. In this study, half-lives of Ketanserin were consistent with historical results.
